# A Spatially Guided Machine-Learning Method to Classify and Quantify Glomerular Patterns of Injury in Histology Images

**DOI:** 10.3390/jimaging9100220

**Published:** 2023-10-11

**Authors:** Justinas Besusparis, Mindaugas Morkunas, Arvydas Laurinavicius

**Affiliations:** 1 Faculty of Medicine, Vilnius University, M.K.Ciurlionio 21, LT-03101 Vilnius, Lithuania; mindaugas.morkunas@santa.lt (M.M.); arvydas.laurinavicius@vpc.lt (A.L.); 2 National Center of Pathology, Affiliate of Vilnius University Hospital Santaros Clinics, P. Baublio 5, LT-08406 Vilnius, Lithuania

**Keywords:** convolutional neural network, artificial intelligence, kidney image analysis, digital pathology, glomerular injury pattern assessment

## Abstract

Introduction The diagnosis of glomerular diseases is primarily based on visual assessment of histologic patterns. Semi-quantitative scoring of active and chronic lesions is often required to assess individual characteristics of the disease. Reproducibility of the visual scoring systems remains debatable, while digital and machine-learning technologies present opportunities to detect, classify and quantify glomerular lesions, also considering their inter- and intraglomerular heterogeneity. Materials and methods: We performed a cross-validated comparison of three modifications of a convolutional neural network (CNN)-based approach for recognition and intraglomerular quantification of nine main glomerular patterns of injury. Reference values provided by two nephropathologists were used for validation. For each glomerular image, visual attention heatmaps were generated with a probability of class attribution for further intraglomerular quantification. The quality of classifier-produced heatmaps was evaluated by intersection over union metrics (IoU) between predicted and ground truth localization heatmaps. Results: A proposed spatially guided modification of the CNN classifier achieved the highest glomerular pattern classification accuracies, with area under curve (AUC) values up to 0.981. With regards to heatmap overlap area and intraglomerular pattern quantification, the spatially guided classifier achieved a significantly higher generalized mean IoU value compared to single-multiclass and multiple-binary classifiers. Conclusions: We propose a spatially guided CNN classifier that in our experiments reveals the potential to achieve high accuracy for the localization of intraglomerular patterns.

## 1. Introduction

The diagnosis of glomerular diseases is based on the visual assessment of histological patterns of injury, commonly represented as categories in classifications of glomerulonephritis (GN) [1,2,3,4,5,6]. A broad spectrum of histological glomerular lesions was represented with 47 different definitions by Haas et al. [7]. In addition to deciding on a predominant glomerular pattern of injury in the tissue sample, pathologists have to take into account many details that may have focal and segmental distributions and disclose important diagnostic and/or prognostic features. The task becomes further complicated by the occurrence of mixed patterns of injury and potential variance of the findings in consecutive tissue sections. This can be regarded as a phenomenon of intra- and interglomerular heterogeneity, which obscures the accuracy and precision of the assessment.

The increasing need to diagnose renal pathologies to guide therapy decisions has led to the implementation of pathology scoring schemes for different types of GN, in particular, lupus nephritis, ANCA GN and IgA nephropathy [8,9]. For example, lupus nephritis is categorized into class II, III or IV based on the presence and spread of global and segmental endocapillary lesions, while chronicity is represented by global/segmental glomerulosclerosis, often in the same glomeruli. This semi-quantitative assessment then converges into broader categories of disease activity and chronicity [1,10]. In the case of lupus nephritis, the categories are associated with clinical consequences: class II representing a rather indolent renal disease and classes III and IV being related to increasingly aggressive courses of the disease [11].

GN scoring systems have been used by renal pathologists worldwide but with medium to low reproducibility [12,13]. A recent systemic review by Dasari et al., indicated poor inter-pathologist agreement on assessing the activity index of lupus nephritis, raising doubts about accurate representation of the activity level of the disease for patient management [14]. Furthermore, a published consensus of the definitions of glomerular injury patterns only moderately improved interobserver agreement in identification of glomerular lesions (from 65.2% to 74.8%) [15].

Recent progress in digital image analysis and machine-learning applications has opened new prospects for automated renal pathology assays for both segmentation and quantification tasks [16,17,18,19,20,21,22,23,24,25,26,27,28,29,30,31,32,33,34,35]. Several studies have shown deep-learning algorithms for automated recognition and segmentation of kidney histology compartments. The first convolutional neural network application for multiclass segmentation, published by Hermsen et al., shows a high segmentation performance of glomeruli, interstitium and tubuli and indicates significant relations between the quantification classes of CNN and visually scored components of the Banff classification system [16]. The main histological structures of kidney tissue (glomeruli, proximal/distal tubules, peritubular capillaries and arteries) were also successfully segmented and validated by Jayapandian et al., across multiple stains and pathology laboratories [29]. Although segmentation alone is of limited clinical use, it enables further compartment-specific analyses of kidney histology [36].

CNN applications for glomerular quantification and classification tasks have been advancing over recent years. Gallego et al., proposed a framework based on the U-Net model that can reliably segment and classify normal and sclerosed glomeruli in whole slide images with the F1-scores of 94.5% and 76.8%, respectively [37]. Ginley et al., defined a validated set of digital characteristics that quantify the structural progression of diabetic nephropathy. The digital classification agreed with a classification provided by an experienced pathologist with moderate Cohen’s kappa 0.55 [95% confidence interval 0.50–0.60] [38]. Zeng et al., proposed a CNN-aided quantitative analysis of glomerular pathology feature classification in IgA nephropathy with a total Cohen’s kappa of 0.912 [95% confidence interval 0.892–0.932] [39]. Weis et al., tested various CNN methods and proposed a CNN-based approach to simultaneously assess various glomerular lesions with convincingly good classification results (Cohen’s kappa values 0.838–0.938) [33]. Yang et al., explored the possibilities of an integrated classification model to determine various patterns of glomerular disease in whole slide images with AUC values ranging from 0.687 to 0.947 for scorable glomerular lesions [31]. In general, current classification systems for glomerular disease require at least six injury patterns (mesangioproliferative, endocapillary, membranoproliferative, crescentic, segmental glomerulosclerosis and global glomerulosclerosis) to be recognized and quantified. However, only one study aimed to apply a CNN-based approach to focus on classifying all these patterns in a single model and achieved classification accuracies with a ranging κ coefficient from 0.28 to 0.50 [40]. To comprehensively assess glomerular histopathology and to provide better context for segmental lesions, other “less-specific” patterns (e.g., normal, membranous and hypertrophy) are required.

To the best of our knowledge, until now, no CNN-based approach has been reported that is focused on intraglomerular classification and quantification of essential injury patterns for grading systems to be tested against manually predefined regions. Previous experiments achieved acceptable accuracies for glomerular injury classification with a gradient-weighted class activation mapping technique (Grad-CAM) to visualize the performance of the classifier [31,33,40]. This technique is a post hoc neural network attention method that is not utilized for classifier training. Regarding the segmentation of glomerular histological patterns, it can show an accurate feature recognition or sometimes might present false negative intraglomerular segmentation results [31,33,40]. However, Grad-CAM heatmaps contain valuable spatial information that could potentially be utilized to train a CNN-based classifier.

In this study, we present a cross-validated comparison of different modifications of CNN-based models for glomerular pattern classification and demonstrate that model performance could be improved by spatially focused guidance. For accurate recognition and quantification of intraglomerular patterns, we propose a method of a spatially guided multiclass CNN classifier that is validated by comparing classifier-produced attention heatmaps to manual annotations provided by nephropathologists.

## 2. Material and Methods

### 2.1. Patient Specimens, Digital Image Acquisition and Image Preprocessing

The study is based on a retrospective collection of 695 routine renal biopsies performed and tested at the National Center of Pathology (Vilnius, Lithuania) from 2016 to 2021. The cohort was balanced by a final pathology diagnosis that contained 100 cases of IgA nephropathy, 99 cases of membranoproliferative glomerulonephritis, 100 cases of crescentic glomerulonephritis, 100 cases of membranous nephropathy, 96 cases of minimal change disease, 35 cases of secondary focal segmental glomerulosclerosis and 92 biopsies of endocapillary glomerulonephritis. Formalin-fixed paraffin-embedded routine renal biopsies were cut in 3 μm-thick sections and stained by modified Picrosirius Red stain. Digital whole slide images (WSI) were recorded using a ScanScope XT Slide Scanner (Leica Aperio Technologies, Vista, CA, USA) under 20× objective magnification and 0.5-μm resolution and subsequently subjected to digital image analysis by using HALO^TM^ software (version 3.5.3577.140 and HALO AI 3.5.3577; Indica Labs, Corrales, New Mexico, United States) for glomerular segmentation. Based on manual annotations, the HALO AI DenseNet classifier was trained to recognize and segment glomeruli containing all types of injury patterns. HALO classifier prediction masks were used to create a collection of glomeruli cropped from original biopsy WSI into 1024 × 1024 pixel-sized images. A total of 27,156 glomerular images were extracted and preprocessed by replacing surrounding renal cortex tissue with a black background.

### 2.2. Ethics Declarations

All tissue samples originated from the Lithuanian National Center of Pathology, and the study was performed with the permission of the Vilnius Regional Biomedical Research Ethics Committee No. 2019/6-1148-637. Informed consent was waived by the Vilnius Regional Biomedical Research Ethics Committee, and all methods were performed in accordance with relevant guidelines and regulations.

### 2.3. Defining Glomerular Injury Patterns and Datasets for Classification

**Figure 1:**
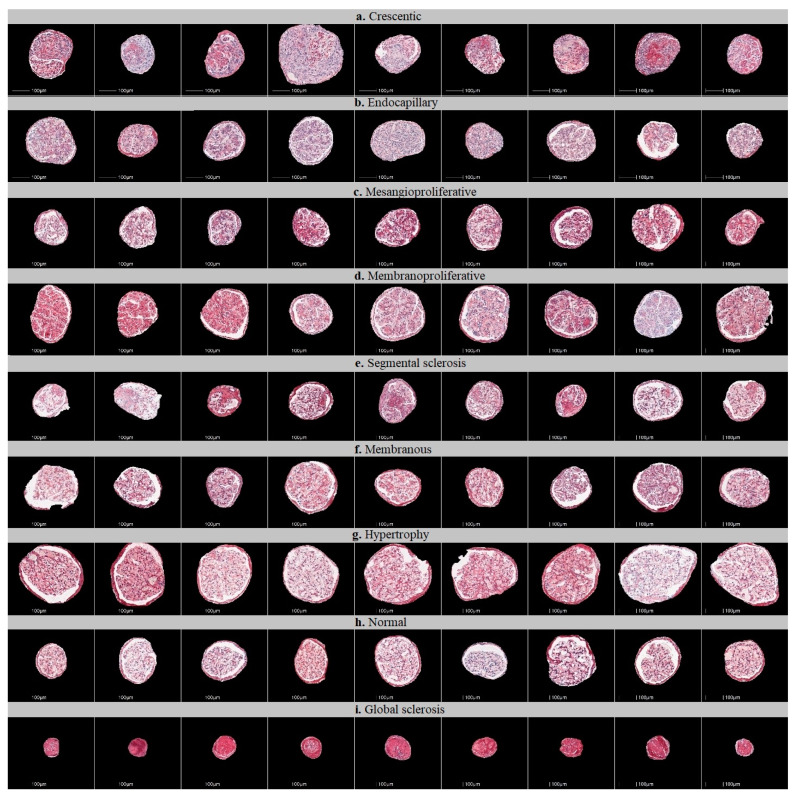
Examples of glomerular patches containing different patterns of injury used to train the classifiers. Nine classes of glomerular patterns of injury were used: (**a**) crescentic, (**b**) endocapillary, (**c**) mesangioproliferative, (**d**) membranoproliferative, (**e**) segmental sclerosis (FSGS), (**f**) membranous, (**g**) hypertrophy, (**h**) normal glomeruli, (**i**) global sclerosis.

All glomerular images that were extracted were reviewed and categorized into nine injury patterns: mesangioproliferative, endocapillary, membranoproliferative, membranous, crescentic, segmental glomerulosclerosis, hypertrophy, global glomerulosclerosis or normal glomeruli (Figure 1). First, glomerular images were sorted by the main diagnosis of the biopsy. Then, the images of glomerular injury patterns, as uniform as possible (avoiding mixed patterns), were preselected by the consensus of two nephropathologists (J.B., A.L.) who blindly reviewed images for CNN training. Glomeruli that represented mixed or ambiguous patterns of injury were not included in the training set. Images of cropped glomeruli representing pure patterns were randomly assigned to testing and training sets. Images of cropped glomeruli representing pure patterns were randomly assigned to the testing and training sets.

We doubled the number of glomeruli images in a training set by rotational augmentation; each original image of a cropped glomerulus was rotated by individually selecting a random angle of rotation in 90° steps (one of 90°, 180° or 270°). The complete composition of both training and testing (holdout) sets is given in Table 1.

### 2.4. Workflow of the Study

**Figure 2:**
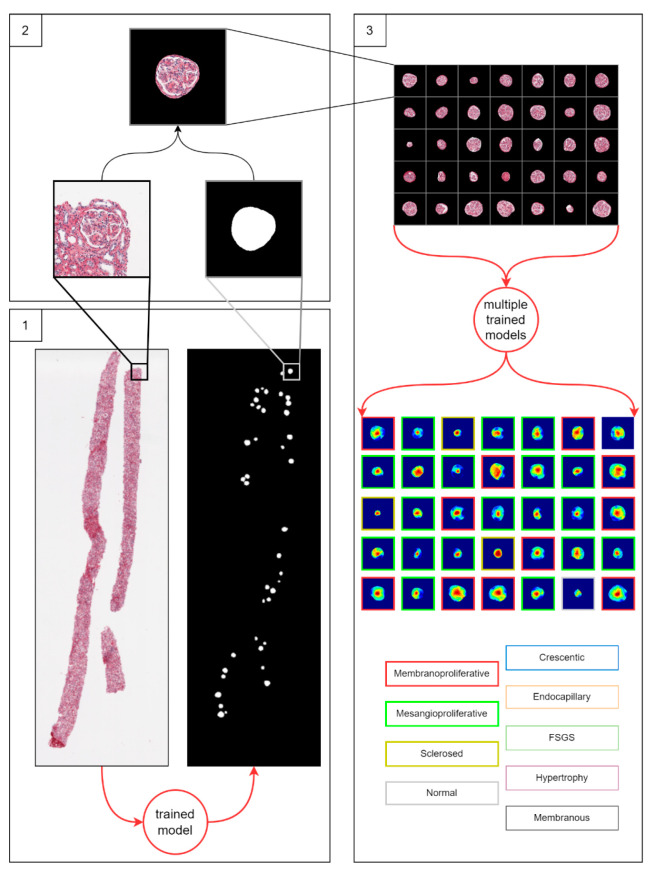
The three-step workflow for glomerulus classification. Step 1: detection of glomeruli in a biopsy by trained HALO AI (Indica Labs) classifier model. Step 2: preprocessing of detected glomeruli images. Preprocessing includes centering of a glomerulus area in a 1024 × 1024 pixel-sized patch and discarding non-glomerulus background pixels. Step 3: classification of all preprocessed glomeruli by the differently trained classifiers. Three classification approaches were used in step 3: multiple binary classifiers trained in the one-vs-rest setting, a single multiclass classifier and a spatially guided multiclass classifier. The classifier models were trained in a five-fold cross-validation setting. The result of the workflow is a set of classified glomeruli instances and corresponding attention heatmaps. We compare the three classification approaches by classification accuracy and area under curve metrics. For each classification approach, we provide neural attention visualization (heatmaps) of the trained model. We compare attention heatmaps of the three classification approaches versus nephropathologists’ annotations by intersection over union metrics.

The workflow of the study is outlined in Figure 2. The workflow consists of three major steps. First, WSIs of kidney biopsies were subjected to analysis by a trained HALO AI model to produce WSI-scale binary glomeruli segmentation masks stored alongside the original images. Second, in the WSI-scale masks, centroid coordinates were found for all glomeruli. This was achieved by labeling all connected regions and finding bounding boxes for all objects in a labeled array (using the *skimage* and *scipy* libraries in Python). Centroid coordinates were then used to crop out 1024 × 1024 pixel-sized patches from both the original images and corresponding binary masks. By multiplying both cropped images (the mask and the original), we produced a masked glomerulus image; the images were then randomly assigned to the training and testing datasets. Finally, glomerular classifiers were trained and evaluated. To interpret and explain the classification criteria used by classifiers during inference, we visualized discriminative regions of glomeruli images relevant to distinct injury patterns. For this task, we used a gradient-weighted class activation mapping technique that captures spatial information that is preserved through convolutional layers of a trained classifier [41]. The localization heatmap is calculated as a weighted sum of feature maps in the final convolutional layer of the classifier and up-sampled to match the size of the original image. For display, the up-sampled Grad-CAM localization heatmaps are overlayed on top of the glomerular images.

In this study, we aimed to achieve both better classification accuracy and better quality localization heatmaps. We first approached this problem with a multiclass classifier, then with multiple binary classifiers and finally with the proposed novel “spatially guided” multiclass classifier.

#### 2.4.1. Multiclass Classification of Glomerular Injury Patterns by a Single Artificial Neural Network-Based Classifier

We used the training set to develop classifiers for the nine patterns of glomerular injury. First, we built a single nine-class classifier based on an ImageNet-pretrained Xception neural network architecture [42]. We reconfigured the Xception model to accept input images with a size of 1024 × 1024 pixels. For each instance of glomerulus, the final classification layer of the model was set to generate a nine-class probability output via the softmax activation function. The classifier was trained with a balanced dataset by feeding the model with four image batches. The training was guided by a stochastic gradient descent (SGD) optimizer that minimizes categorical cross-entropy loss.

#### 2.4.2. One-vs-Rest Classification of Glomerular Injury Patterns by Multiple Binary Classifiers

Second, we performed a binary classification of glomerular injury patterns in a one-vs-rest setting by splitting the nine-class dataset into nine binary classification tasks (the complete composition of the one-vs-rest training dataset is given in Supplementary Table S1). For this task, we built nine distinct binary Xception-based classifiers, each directed to discriminate a particular glomerular injury type from remaining classes. For each binary classifier, we again reconfigured the original Xception architecture for an input with a size of 1024 × 1024 pixels, but the final classification layer was set to generate a single-class probability output via the sigmoid activation function. One-vs-rest classifiers were trained by an SGD optimizer to minimize binary cross-entropy loss. Since binarization of training labels introduces class imbalance, we therefore balanced the training set by sampling all the glomeruli from the positive (target) class and proportionally subsampling remaining classes to collect an equivalent number of glomeruli to represent a negative class. During inference for a particular glomerulus, each binary classifier predicts a class membership probability score. The argmax of these scores defines the overall predicted class of the glomerulus injury pattern.

#### 2.4.3. Spatially Guided Multiclass Classification of Glomerular Injury Patterns

Grad-CAM is a post hoc neural network attention method, which means that it does not participate in the classifier training. Localization heatmaps are not learned specifically and are not influenced by any particular model training parameters. Moreover, in Grad-CAM, up-sampling is achieved in a single step going from a tiny final convolutional layer-sized localization heatmap up to an input image-sized final heatmap by an integer factor that typically is of the order of dozens, meaning that the final heatmap is coarse and noisy.

#### 2.4.4. Proposed Neural Network Architecture

**Figure 3:**
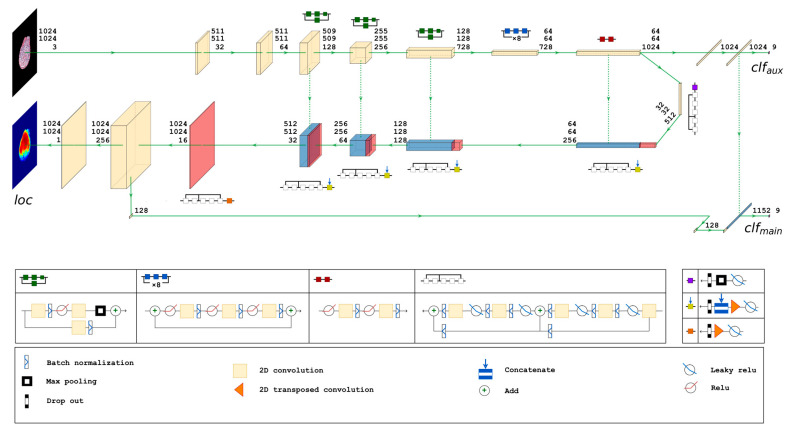
Detailed schema of the proposed neural network architecture of a spatially guided multiclass classifier.

Therefore, to improve classifier focus on essential parts of the image and increase attention localization heatmap granularity, we attempted to build a trainable attention mechanism. For this task, we employed a U-Net-like encoder–decoder structure. We again used the Xception architecture as the base model, but we modified it with U-Net-style decoder and skip connections. We designed the network with three output layers. The final convolutional layer of the Xception architecture on the encoder branch is connected to an aggregation block consisting of a global average pooling operation and an intermediate densely connected layer ($int_{1}$). This block feeds the first (auxiliary) densely connected output layer ($clf_{aux}$) that has a softmax activation function to produce a nine-class probability output. A similar block (global average pooling followed by dense intermediate ($int_{2}$)) is added at the end of the decoder branch. The second (main) dense classification layer ($clf_{main}$) receives concatenated output of intermediate dense layers ($int_{1}$) and ($int_{2}$) produces another nine-class probability output via the softmax activation function. In parallel to the ($clf_{main}$) branch, a single neuron 1 × 1 2D convolutional layer acts as the third output layer (loc) that produces a localization heatmap exactly matching the input image size. The main classification branch ($clf_{main}$) is therefore conditioned to depend upon both localization (loc) and auxiliary classification ($clf_{aux}$) branches. The detailed schema of the proposed neural network architecture is given in Figure 3.

During training, the network learns to assign features of the glomeruli images to the corresponding ground truth class labels of distinct glomerular injury patterns ($clf_{aux}$ and $clf_{main}$) and a functional mapping between pixels in glomeruli images and the pixels in corresponding ground truth localization heatmaps (loc).

The model is configured to accept 1024 × 1024 pixel-sized glomeruli images and is trained by an optimizer independently minimizing three weighted loss functions: categorical cross-entropy loss functions for $clf_{aux}$ and $clf_{main}$ classification outputs and a binary cross-entropy for localization heatmap output (loc). Weighting loss functions enable prioritization of network tasks, e.g., main classification over auxiliary classification ($w_{clf_{main}}>>w_{clf_{aux}}$) and localization slightly over main classification ($w_{clf_{main}}>w_{loc}$). We trained the model with the following constraints: $w_{clf_{main}}+w_{clf_{aux}}+w_{loc}=1$ and $w_{loc}>w_{clf_{main}}\gg w_{clf_{aux}}$.

#### 2.4.5. Ground Truth Localization Heatmaps

**Figure 4:**
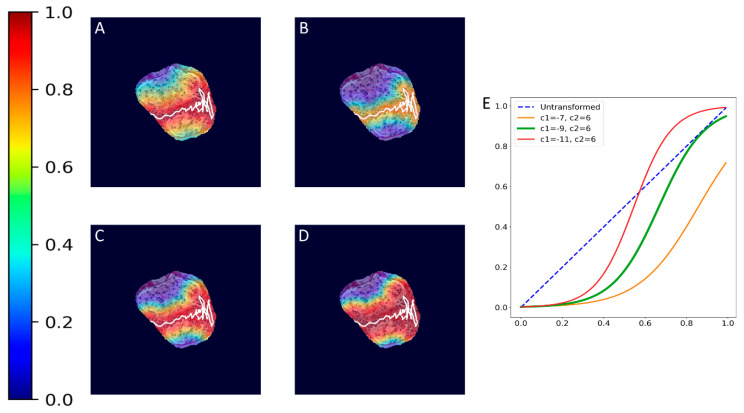
Annotation examples with different values of heatmap transformation coefficients ($c_{1},$ $c_{2}$) applied. The freeform annotation (white line in panels A to D) is placed on an image of the glomerulus. Localization heatmaps were generated with different values of transformation coefficients ($c_{1},$ $c_{2}$): (**A**)—untransformed (linear), (**B**)—(−7, 6), (**C**)—(−9, 6), and (**D**)—(−11, 6). (**E**)—corresponding transformation functions (solid lines) plotted against untransformed data (dashed line). The nonlinearity introduced by the transformation can be seen as a steep transition from low to high values in the function plot and as a sharp blue-to-red transition in a heatmap. The transformation coefficients were balanced to put emphasis on regions of interest inside the image of the glomerulus. For use in this paper, transformation coefficients ($c_{1}=–9$, $c_{2}=6)$ were selected by nephropathologists’ visual appreciation of the resulting ground truth localization heatmaps.

To train the proposed spatially guided classifier, ground truth localization heatmaps were generated. Firstly, nephropathologists were asked to highlight hotspots of segmental glomerular injury patterns by placing a simple freeform annotation (as small as a single pixel) in a copy of the glomeruli training set. Multiple hotspots were allowed. Similarly, annotations of diffuse patterns and normal glomeruli were needed. In these cases, annotations cover the full area of glomeruli, and therefore the annotation process was achieved automatically by placing a single pixel annotation at the centroid of the glomerular mask. These annotations were then transformed into heatmaps in which every pixel in a heatmap receives a value through a nonlinear distance-based function:

(1) $$h_{x,y}=\frac{1}{1+e^{-\left(c_{1}\cdot d_{x,y}+c_{2}\right)}}$$

where $h_{x,y}$ is the value of a pixel at x,y position in an image plane, $d_{x,y}$ is the Euclidean distance from that pixel to the nearest pixel in an annotation and $c_{1}$ and $c_{2}$ are preselected constants. Briefly, pixels closer to the annotation receive values closer to 1.0, and pixels further from the annotation receive values closer to 0.0. All pixels outside the glomerulus contour receive 0.0 values. Examples of localization heatmaps are shown in Figure 4.

#### 2.4.6. The Cross-Validation Scheme

The cross-validation (CV) procedure employed in our study was carefully designed to ensure robust and unbiased model evaluation. Firstly, we divided the dataset into training and testing sets. The test set was selected entirely randomly and well before the actual experimentation. Notably, the test set remained unchanged throughout the study to facilitate fair and comparable assessments of all developed models. This strategy was meticulously implemented to mitigate the risk of any knowledge transfer from the training phase into the test set, thereby preserving the transparency of the evaluation process. Subsequently, the training set was formed from the remaining glomeruli images and served as the foundation for all model development. To estimate model performance, we adopted a five-fold cross-validation scheme. In each iteration of the CV, four of the CV folds were utilized for training purposes while one fold was reserved for validation. As a result, we obtained five independent instances of a model, each trained on a unique composition of training and validation images. Following the completion of the CV iterations, five distinct model instances were available, each reflecting different training conditions. Subsequently, all five model instances were rigorously assessed using the test set, ensuring a consistent and equitable evaluation process. We used this CV procedure to train and evaluate single-multiclass, multiple-binary and the proposed spatially guided classifiers.

### 2.5. Metrics

Accuracy was used to monitor all classifier models during the training phase (see Supplementary Figure S1 for model training metrics). During inference, classifier performance was compared by the area under the ROC curve metrics (AUC), and multiclass confusion matrices were used to gain deeper insights of classification errors and biases. We compare classifier experiments in a multiclass classification setting, and therefore mean classification accuracy over cross-validation folds reported per class in the Results section is calculated as true positive rate. The amount of variance in classifier performance is reported by the standard deviation of accuracies (coefficients of variance are employed to identify values exceeding average). Generalized classification accuracy is calculated over the diagonal of an aggregated multiclass confusion matrix. The quality of predicted localization heatmaps was evaluated by intersection over union metrics between predicted and ground truth localization heatmaps at a threshold value. Furthermore, quantification of the injury pattern area was measured by a percentage of the predicted intraglomerular pattern heatmap.

### 2.6. Implementation

All image data manipulation steps (preprocessing of glomeruli images and generation of augmented images, ground truth heatmaps and figures in the manuscript) were done in Python 3.8.10 (Python Software Foundation, Wilmington, DE, USA) using ‘scikit image’ v.0.19.1 (https://doi.org/10.7717/peerj.453 (accessed on 30 August 2023), ‘matplotlib’ v.3.5.1 (https://doi.org/10.5281/zenodo.5773480 (accessed on 30 August 2023)), ‘numpy’ v.1.20.0 (https://doi.org/10.1038/s41586-020-2649-2 (accessed on 30 August 2023)) and ‘scipy’ v. 1.7.3 (https://doi.org/10.1038/s41592-019-0686-2 (accessed on 30 August 2023)) libraries. The classifiers were built, trained and evaluated in Python 3.8.10 using ‘tensorflow’ v.2.7.0 and ‘scikit-learn’ v.1.0.2 on a high-performance graphical processing unit (Nvidia GeForce RTX 3090).

## 3. Results

**Figure 5:**
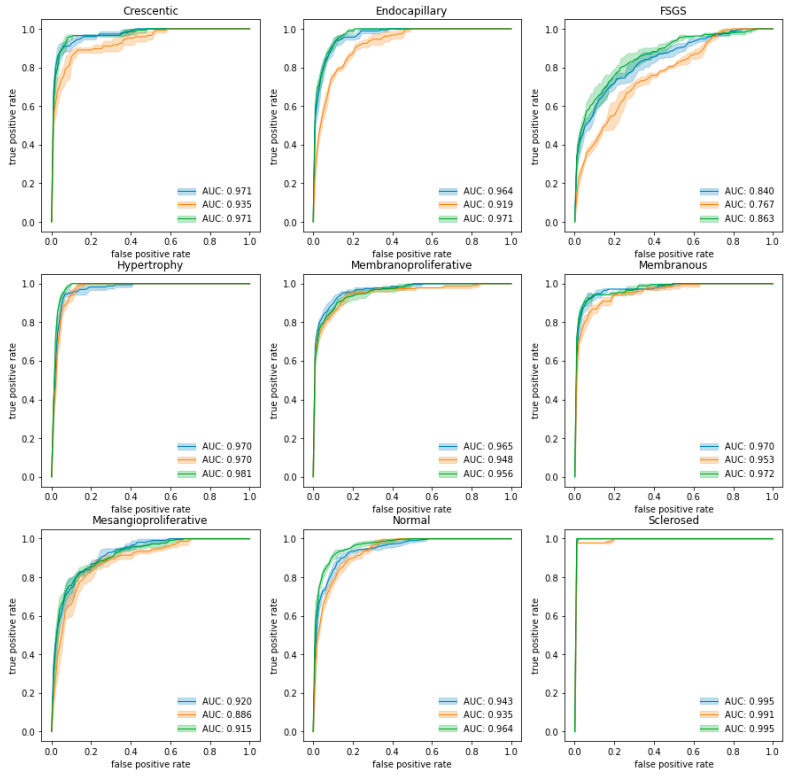
ROC curves and means of AUC scores by class for each classification experiment. The blue curve indicates the performance of the single-multiclass classifier, the orange curve indicates the performance of the multiple-binary classifier and the green curve indicates the spatially guided CNN classifier.

We conducted all classifier experiments in a five-fold cross-validation setting as described in Section 2.4.6. Trained classifiers were evaluated in a testing (holdout) set. Averaged ROC curves are presented in Figure 5, and the detailed results are presented in Supplementary Tables S2–S4. Mean classification metrics were obtained by averaging class-specific results for all folds in each of the classification experiments and are given in Table 2.

### 3.1. Classification of Glomeruli Patterns

Generalized classification accuracies in different classifier experiments range from 0.677 for the multiple-binary classifier up to 0.728 for the spatially guided classifier. The highest accuracy per class was observed for the sclerosed glomeruli pattern by all classifiers (mean of all ~0.985). Similarly, the lowest accuracy among the classifiers was obtained for the FSGS pattern (mean of all ~0.473). We observed an overall tendency towards higher classification accuracy for diffuse glomeruli (e.g., membranoproliferative, membranous and hypertrophy) patterns (mean ~0.817), while segmental injury patterns were more difficult to discriminate (mean ~0.626). The spatially guided classifier achieved the highest mean classification accuracies for diffuse (~0.830) and segmental (~0.659) patterns, while the multiple-binary classifier achieved the lowest accuracies for both diffuse (~0.806) and segmental (0.560).

**Table 2:** Mean classification accuracy metrics per class. Highlighted cells (in orange) identify values that exceed the mean coefficient of variance for the given experiment (9.90%, 9.59% and 12.06% for single-multiclass, multiple-binary and spatially guided classifiers, respectively). The mean IoU and the corresponding standard deviation metrics were calculated over five cross-validation folds. Individual IoU scores were computed at a 0.5 threshold (see Supplementary Tables S2–S4 for detailed cross-validation classification metrics). The IoU measure was not calculated for diffuse patterns (marked n/a).

Classifier Experiment	Crescentic	Endocapillary	FSGS	Mesangioproliferative	Membranoproliferative	Membranous	Hypertrophy	Normal	Sclerosed	Generalized Multiclass
	Segmental Injury				Diffuse					
	Mean Classification Accuracy (Standard Deviation)									
Single-multiclass	0.841 (0.046)	0.730 (0.118)	0.478 (0.076)	0.586 (0.148)	0.765 (0.060)	0.817 (0.066)	0.879 (0.057)	0.640 (0.025)	0.978 (0.000)	0.719 (0.010)
Multiple-binary	0.745 (0.072)	0.573 (0.147)	0.437 (0.025)	0.486 (0.080)	0.757 (0.082)	0.840 (0.048)	0.830 (0.051)	0.625 (0.039)	0.978 (0.000)	0.677 (0.006)
Spatially guided	0.814 (0.076)	0.676 (0.128)	0.504 (0.072)	0.643 (0.154)	0.739 (0.063)	0.840 (0.082)	0.927 (0.046)	0.644 (0.120)	1.000 (0.000)	0.728 (0.028)
	Mean AUC (standard deviation)									
Single-multiclass	0.971 (0.005)	0.964 (0.006)	0.840 (0.014)	0.920 (0.010)	0.965 (0.005)	0.970 (0.004)	0.970 (0.005)	0.943 (0.007)	0.995 (0.000)	0.949 (0.002)
Multiple-binary	0.935 (0.013)	0.919 (0.006)	0.767 (0.006)	0.886 (0.012)	0.948 (0.003)	0.953 (0.001)	0.970 (0.003)	0.935 (0.006)	0.991 (0.000)	0.923 (0.003)
Spatially guided	0.971 (0.003)	0.971 (0.003)	0.863 (0.020)	0.915 (0.010)	0.956 (0.005)	0.972 (0.003)	0.981 (0.003)	0.964 (0.003)	0.995 (0.000)	0.954 (0.004)
	Mean IoU (standard deviation)									
Single-multiclass	0.061 (0.012)	0.050 (0.006)	0.042 (0.003)	0.041 (0.003)	n/a	n/a	n/a	n/a	n/a	0.049 (0.003)
Multiple-binary	0.060 (0.006)	0.052 (0.007)	0.034 (0.012)	0.049 (0.016)	n/a	n/a	n/a	n/a	n/a	0.048 (0.007)
Spatially guided	0.404 (0.174)	0.379 (0.138)	0.263 (0.116)	0.235 (0.114)	n/a	n/a	n/a	n/a	n/a	0.320 (0.133)

While classification accuracy is a straightforward and intuitive metric that directly indicates proportion of correct predictions, AUC metrics add to the evaluation a probabilistic component of ranking predictions. The mean of AUC scores indicates that the spatially guided classifier has a higher prediction confidence for most (seven out of nine) glomeruli patterns compared to other approaches. AUC scores generalized to all glomeruli patterns in all classifier experiments exceeded 0.900, with the spatially guided classifier being the most accurate and confident (generalized AUC score of 0.954).

The consistency of all classifiers can be inferred from the standard deviations reported for the classification metrics. The highlighted cells in Table 2 identify values that exceed the mean coefficient of variance for the given experiment (9.90%, 9.59% and 12.06% for the single-multiclass, multiple-binary and spatially guided classifiers, respectively). A higher amount of variance in classifier performance can be observed for segmental injury patterns.

**Figure 6:**
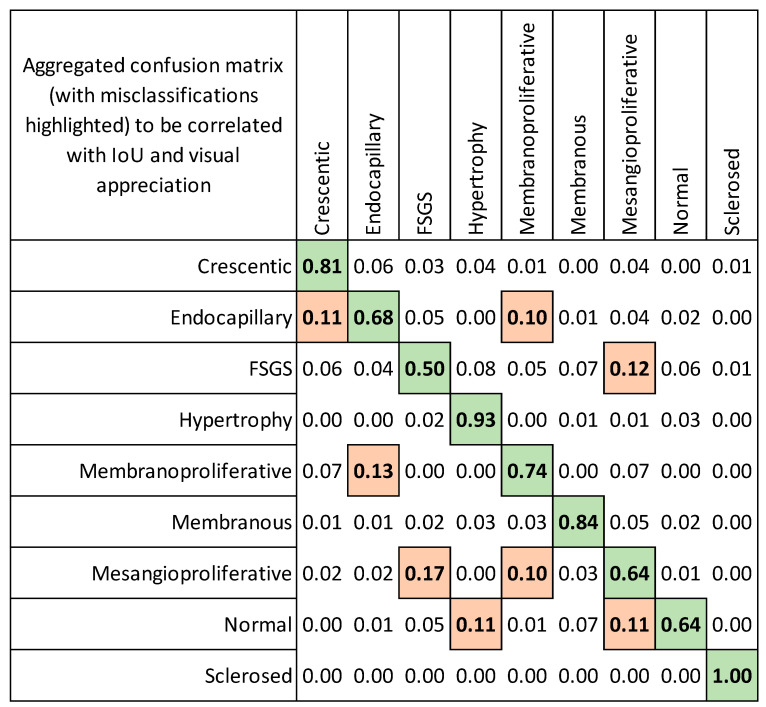
The confusion matrix of a spatially guided classifier. The orange color in the confusion matrix highlights the misclassification–the most confused patterns (averaged over cross-validation folds). The green color in the confusion matrix highlights the best classification results.

The highest pairwise misclassification rates (Figure 6) were observed between the following groups: endocapillary patterns and crescentic/membranoproliferative patterns (0.11 and 0.1), FSGS and mesangioproliferative changes (0.12), membranoproliferative and endocapillary (0.13), mesangioproliferative and FSGS (0.17), normal and hypertrophy/mesangioproliferative (0.11).

### 3.2. Evaluation of Localization Heatmaps and Pattern Quantification

Ground truth localization heatmaps were generated for glomeruli images in a training set (both original and augmented glomeruli images) as well as for ones in a testing (holdout) set, which allowed us to conduct an analysis of concordance between ground truth localization heatmaps and classifier-produced localization heatmaps. An overview of concordance measured by intersection over union is given in Table 2. In general, gradient-based heatmaps merely overlap expert-annotated areas inside the glomeruli with mean IoU values below 0.05 for both single-multiclass and multiple-binary classifiers. In fact, quite often, these classifiers tend to reason glomeruli pattern classification in areas outside glomeruli contour, thus likely capturing shape and size characteristics. Importantly, the spatially guided classifier with trainable localization heatmaps achieved the 0.320 generalized mean IoU value. More in-depth analysis revealing special cases of classifier localization heatmaps is presented in Table 3, Table 4 and Table 5.

Quantification of the intraglomerular injury pattern revealed miscellaneous results depending on a fold of the cross validation set. Detailed comparison of pattern quantification results on a glomerular basis is presented in Supplementary Figure S2, where we present injury pattern segmentation for the complete test dataset (n = 417).

## 4. Discussion

In this study, we exploited several novel opportunities that the use of CNN offers for the recognition and quantification of glomerular injury patterns. First, we proposed a new spatially guided modification of the CNN classifier and applied it for improved classification of glomerular injury patterns. Second, we estimated the accuracy of the localization of intraglomerular injury patterns compared to ground truth annotations produced by nephropathologists and demonstrated the potential of intraglomerular pattern quantification.

Our spatially guided CNN classifier showed the best classification results for most of the investigated glomerular injury patterns compared to single-multiclass and multiple binary classifiers. Importantly, classification precision was high also for segmental patterns (crescentic, endocapillary, FSGS), which are usually more complicated for automated segmentation but are necessary for comprehensive assessment of glomerular pathology. The AUC values for the spatially guided classifier are very close to previously reported AUC measurements for the same patterns. However, our glomerular image datasets used for training purposes are relatively small (only 81 glomeruli for each type of injury without augmentation and 1146 in total) compared to previous studies (for example, a total number of 32,267 glomeruli were used by Yang et al.) [31,40]. This could be seen as a drawback of our study; however, on the contrary, it may indicate the added value of our spatial guidance model for CNN architecture, which provides satisfactory results from significantly smaller datasets.

Imbalanced datasets, in general, present a major issue for machine learning, computer vision, and pattern recognition tasks. Data class imbalances occur when there is a significant inequality between the number of examples of different classes, and, if not addressed, this imbalance greatly impairs classifier detection accuracy [43]. In the field of CNN applications for glomerular pattern recognition, imbalanced datasets were usually used due to low incidence of some patterns such as pure, non-overlapping endocapillary hypercellularity or crescent formation that are relatively rare compared to other pathological features [31,33,40]. In this study, in total, 695 native renal biopsies and 27,156 glomeruli were used to create a balanced dataset for each type of glomerular class in both the training and testing subsets (1146 glomeruli in total). We therefore handled the foreground–foreground class imbalance at the early sampling stage in the object detection pipeline [43].

We preprocessed glomerular images by replacing surrounding renal tissue with a black background to focus our classifiers exclusively on glomerular structures. In contrast to previous studies that included surrounding tissues for classifier training and testing purposes, we preferred to avoid any extraglomerular context by replacing it with black [30,31,33]. It could be argued that this methodological step could introduce a bias and limit potentially useful information in the context of whole renal tissue in the biopsy images. However, our method achieved high accuracies, revealing the good performance of the model. To further explore the impact of this approach, investigations are needed to measure the effects of classifier performance by using various background preprocessing procedures: removal/inclusion, background color, cropping, scaling and the amount of background.

In this study, we compared three different modifications of a CNN classifier in terms of classification and localization accuracy. Although single-multiclass and multiple-binary classifiers showed high accuracy in the prediction of glomerular labels (Table 2), the concordance between ground truth localization heatmaps and classifier-produced localization heatmaps were considerably worse than the spatially guided CNN. Surprisingly, the differences in heatmap overlap area were distinct: the spatially guided classifier achieved a generalized mean IoU of 0.320, compared to only 0.048 and 0.049 for single-multiclass and multiple-binary classifiers, respectively. These results indicate the importance of providing manually annotated images for classifier training if pattern segmentation and quantification are sought. Of note, the heatmap validation procedure was not explored in previous studies of CNN methods for glomerular pattern segmentation [30,31,33].

The analysis of classifier-produced heatmaps and its comparison to manually annotated images on the validation set provided several observations. First, despite the correct label prediction results, single-multiclass and multiple-binary classifiers usually produced poor pattern localization heatmaps (Table 3, row 3; Table 4, rows 1–3, Table 5, row 1). Some of these heatmaps even focused on the black background, ignoring the glomerulus itself. Second, the attention heatmaps of some glomeruli were labeled incorrectly by spatially guided CNN, yet they contained precise annotation of other/less important patterns in the glomerulus. For example, some glomeruli that were labeled as FSGS by an expert were marked as crescentic by a CNN. Further investigation revealed that these glomeruli were taken from a crescentic glomerulonephritis case and were chosen to present an FSGS pattern potentially containing a segmental sclerosed crescentic lesion. Another example was of small mesangioproliferative areas picked up by the spatial CNN heatmap in the glomerulus presenting FSGS in an IgA nephropathy case. These findings indicate that our spatially guided CNN classifier can recognize several patterns within a glomerulus; a procedure to extract these data from the classifier therefore remains to be explored. Weis et al., have developed CNN classifiers that could deal with complex cases and recognize several patterns occurring in the same glomerulus. In contrast to our experiment, that study explored CNN performance in a more systematic way: a different image dataset was used with complex changes that cannot be attributed to any single category by a pathologist [33].

Significant pairwise misclassification rates (Figure 6) between endocapillary patterns and crescentic/membranoproliferative patterns, FSGS and mesangioproliferative changes, membranoproliferative and endocapillary, mesangioproliferative and FSGS, normal glomeruli and hypertrophy could be explained by some similarities of the histological patterns. However, classifier attention heatmaps revealed that some glomeruli, incorrectly assigned to the normal class, showed perfect detection of glomerular structures that contain areas with normal capillary loops on heatmap visualizations (Table 5). We suggest that it could be related to some imbalances in our training dataset. Datasets were balanced by the number of glomeruli but unbalanced by the area of different patterns inside the histology images. For example, an area of normal capillaries was significantly higher than the FSGS area and can be found in the context of several patterns (hypertrophy, FSGS, mesangioproliferative and crescentic). This might have impacted the classifier training results. This phenomenon has been noted previously by Selvaraju et al., and defined as an inherent bias in datasets when the CNN merely focuses on a frequently occurring feature, which is not always a true pathological lesion [41]. This should be taken into account when planning such experiments and could be avoided by subdividing the glomerular image into several parts according to histological lesions and structures [44]. A recent study proposed by Sato et al., illustrates that on patch-based analysis, a CNN classifier could correctly give higher attention to the structures in the images such as cellular components, sclerotic regions or crescent regions than segmentation results performed on entire glomerular images [30].

Our study contains several limitations. First, a stain normalization procedure was not performed prior to CNN training. This step is essential before image analysis applications, especially when multicenter/multilaboratory specimens are used. However, this experiment was performed on histology specimens stained by a single laboratory, providing a modified Picrosirius Red stain that was rather stable in our practice. This staining modification is not a routine method for kidney biopsies in other laboratories, which hinders the possibilities to perform tests of the CNN methods on open image datasets from other medical centers. Second, our test set was curated to encompass the entirety of our glomeruli image dataset, capturing all potential variations in a transparent and equitable manner. While these images were treated as unseen data for reporting purposes, it is important to acknowledge that no additional images were acquired post-model development and training, thus limiting the assessment of the models’ generalization to novel data. Third, the performance of our trained models remains to be tested on mixed glomerular patterns of injury and stratified in a patient-wise manner. Our training and validation sets were composed of glomerular images that contained only as pure as possible histological patterns within a glomerulus. Further testing in real-life cases, with mixed patterns, remains to be performed and assessed against current scoring schemes of glomerular lesions, clinical parameters and kidney outcome data.

## 5. Conclusions

We propose a novel method of a spatially guided CNN classifier for the purpose of recognizing glomerular patterns of injury. We also present a validation procedure of automatically produced classifier heatmaps for intraglomerular localization of lesions and demonstrate the advantages of spatially guided CNN performance.

## Figures and Tables

**Table 1:** Composition of training and testing datasets.

Glomerular Injury Pattern	Testing Set	Training Set		Total Original Glomeruli
	Original	Original	Augmented	
Crescentic	29	81	81	110
Endocapillary	37	81	81	118
FSGS	54	81	81	135
Hypertrophy	33	81	81	114
Membranoproliferative	46	81	81	127
Membranous	35	81	81	116
Mesangioproliferative	42	81	81	123
Normal	96	81	81	177
Sclerosed	45	81	81	126
Total	417	1458		1146

**Table 3:** Heatmap visualizations of comparison classifiers for concordant and true positive glomeruli pattern classification results (cases with high IoU values).

	Original	Annotation	Single Multiclass	Multiple Binary	Spatially Guided
True label: Crescentic	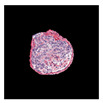	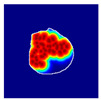	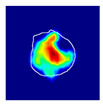	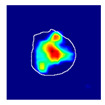	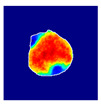
single-multiclass: Crescentic p = 0.999, IoU = 0.154					
multiple-binary: Crescentic p = 1.000, IoU = 0.128					
Spatially guided: Crescentic p = 0.979, IoU = 0.740					
True label: Endocapillary	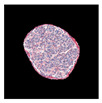	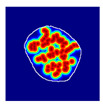	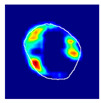	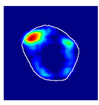	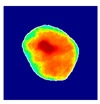
single-multiclass: Endocapillary p = 1.000, IoU = 0.055					
multiple-binary: Endocapillary p = 0.993, IoU = 0.029					
spatially guided: Endocapillary p = 0.976, IoU = 0.710					
True label: FSGS	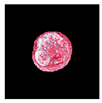	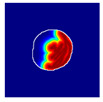	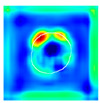	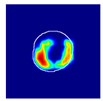	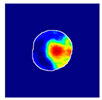
single-multiclass: FSGS p = 0.964, IoU = 0.063					
multiple-binary: FSGS p = 0.999, IoU = 0.076					
spatially guided: FSGS p = 0.862, IoU = 0.390					
True label: Mesangioproliferative	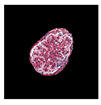	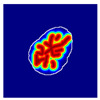	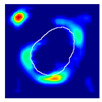	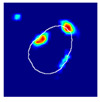	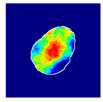
single-multiclass: Mesangioproliferative p = 0.935, IoU = 0.029					
multiple-binary: Mesangioproliferative p = 0.988, IoU = 0.032					
spatially guided: Mesangioproliferative p = 0.873, IoU = 0.201					

**Table 4:** Heatmap visualizations of comparison classifiers for concordant and true positive glomeruli pattern classification results (cases with low IoU values).

	Original	Annotation	Single Multiclass	Multiple Binary	Spatially Guided
True label: Crescentic	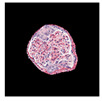	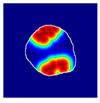	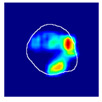	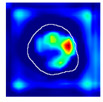	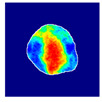
single-multiclass: Crescentic p = 0.940, IoU = 0.058					
multiple-binary: Crescentic p = 0.959, IoU = 0.048					
spatially guided: Crescentic p = 0.983, IoU = 0.163					
True label: FSGS	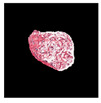	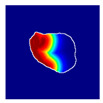	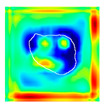	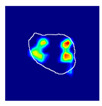	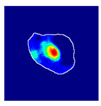
single-multiclass: FSGS p = 0.960, IoU = 0.058					
multiple-binary: FSGS p = 0.986, IoU = 0.041					
spatially guided: FSGS p = 0.774, IoU = 0.015					
True label: Mesangioproliferative	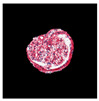	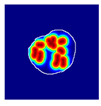	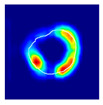	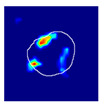	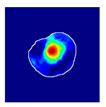
single-multiclass: Mesangioproliferative p = 0.868, IoU = 0.047					
multiple-binary: Mesangioproliferative p = 0.956, IoU = 0.031					
spatially guided: Mesangioproliferative p = 0.840, IoU = 0.141					

**Table 5:** Heatmap visualizations of comparison classifiers for false or discrepant glomeruli pattern classification results.

	Original	Annotation	Single Multiclass	Multiple Binary	Spatially Guided
True label: FSGS	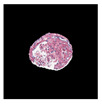	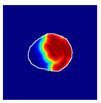	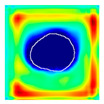	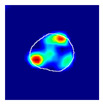	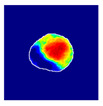
single-multiclass: FSGS p = 0.672, IoU = 0.056					
multiple-binary: Crescentic p = 0.938, IoU = 0.047					
spatially guided: Crescentic p = 0.715, IoU = 0.568					
True label: FSGS	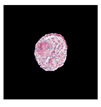	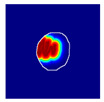	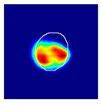	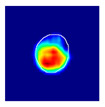	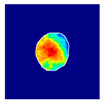
single-multiclass: Crescentic p = 0.949, IoU = 0.102					
multiple-binary: Crescentic p = 0.990, IoU = 0.118					
spatially guided: Crescentic p = 0.966, IoU = 0.449					
True label: FSGS	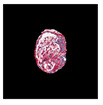	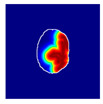	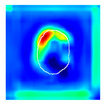	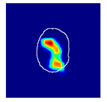	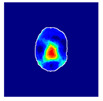
single-multiclass: Normal p = 0.529, IoU = 0.067					
multiple-binary: FSGS p = 0.752, IoU = 0.070					
spatially guided: Mesangioproliferative p = 0.588, IoU = 0.121					
True label: FSGS	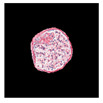	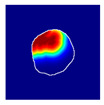	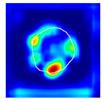	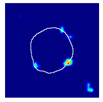	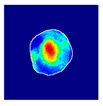
single-multiclass: Normal p = 0.917, IoU = 0.052					
multiple-binary: FSGS p = 0.414, IoU = 0.000					
spatially guided: Normal p = 0.817, IoU = 0.199					
True label: Endocapillary	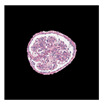	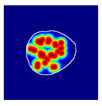	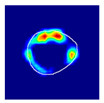	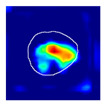	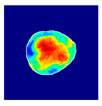
single-multiclass: Endocapillary p = 0.447, IoU = 0.057					
multiple-binary: Crescentic p = 0.766, IoU = 0.081					
spatially guided: Membranoproliferative p = 0.833, IoU = 0.484					

## Data Availability

Glomerular images used for training and testing purposes can be provided upon request. Code for the proposed method is available at: https://github.com/mindEM/glomnet (accessed on 8 October 2023).

## Supplementary Materials

The following supporting information can be downloaded at: https://www.mdpi.com/article/10.3390/jimaging9100220/s1, Figure S1: Model training metrics (loss) from cross validation of all classifiers. Figure S2: Comparison of pattern quantification results per glomerular basis. Table S1: Composition of One-vs-Rest training data set. Table S2: Confusion matrices from cross validation of all classifiers (Single-multiclass). Table S3: AUC scores from cross validation of all classifiers. Table S4: IoU scores (segmental injury patterns only) from cross validation of all classifiers.

## Abbreviations

CNN	convolutional neural network
IoU	intersection over union metrics
GN	glomerulonephritis
Grad-CAM gradient	weighted class activation mapping technique
WSI	whole slide images
FSGS	focal segmental glomerular sclerosis
SGD	stochastic gradient descent
