# Supplementary material for: A Spatially Guided Machine-Learning Method to Classify and Quantify Glomerular Patterns of Injury in Histology Images
Source: J Imaging. 2023 Oct 11;9(10):220. doi: 10.3390/jimaging9100220 (PMC10607091; doi:10.3390/jimaging9100220)
Supplement: Supplementary file 1 [file jimaging-09-00220-s001.zip › Supplementary Figure S1.pdf]

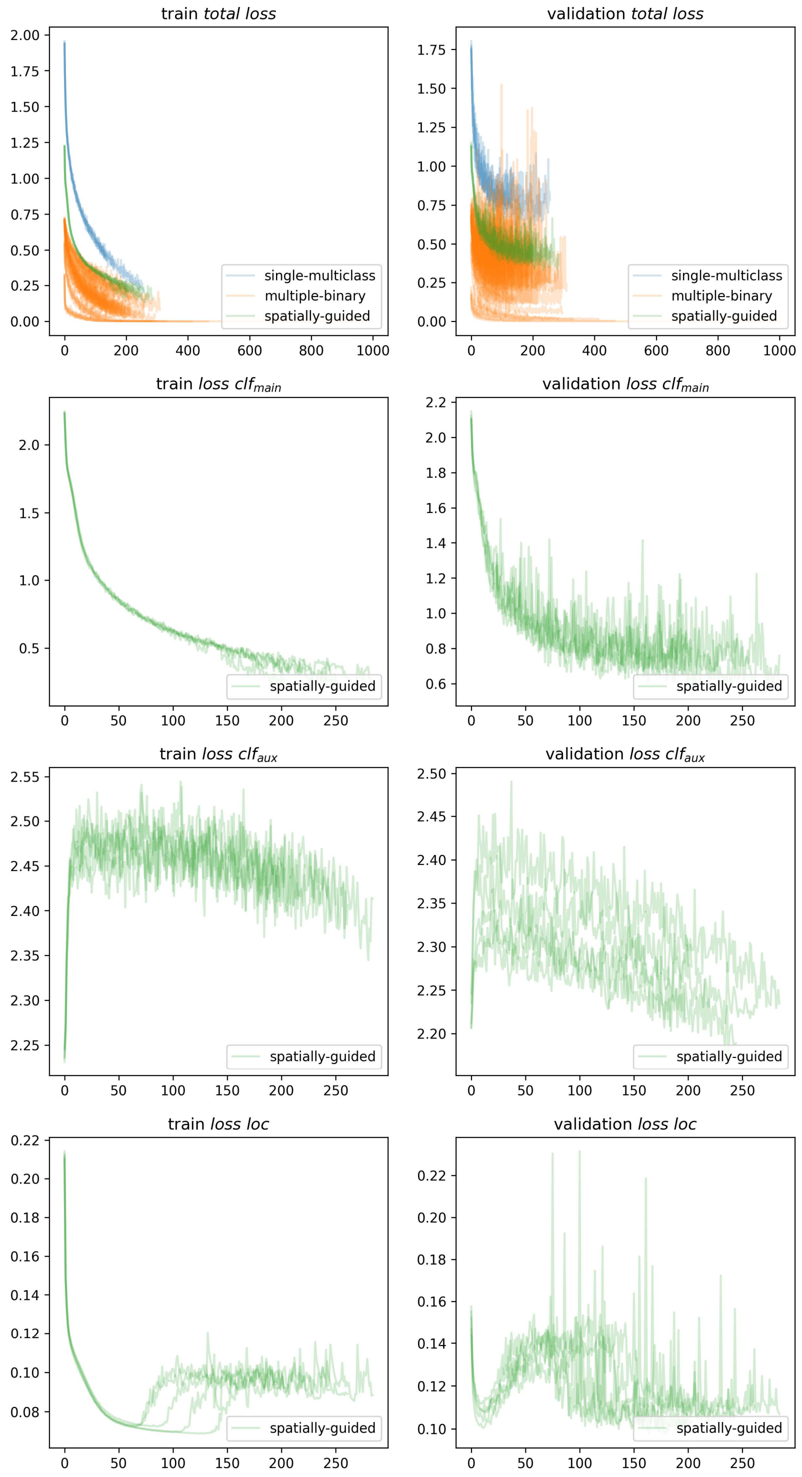

Supplementary Figure S1. Model training metrics (loss) from cross validation of all classifiers.

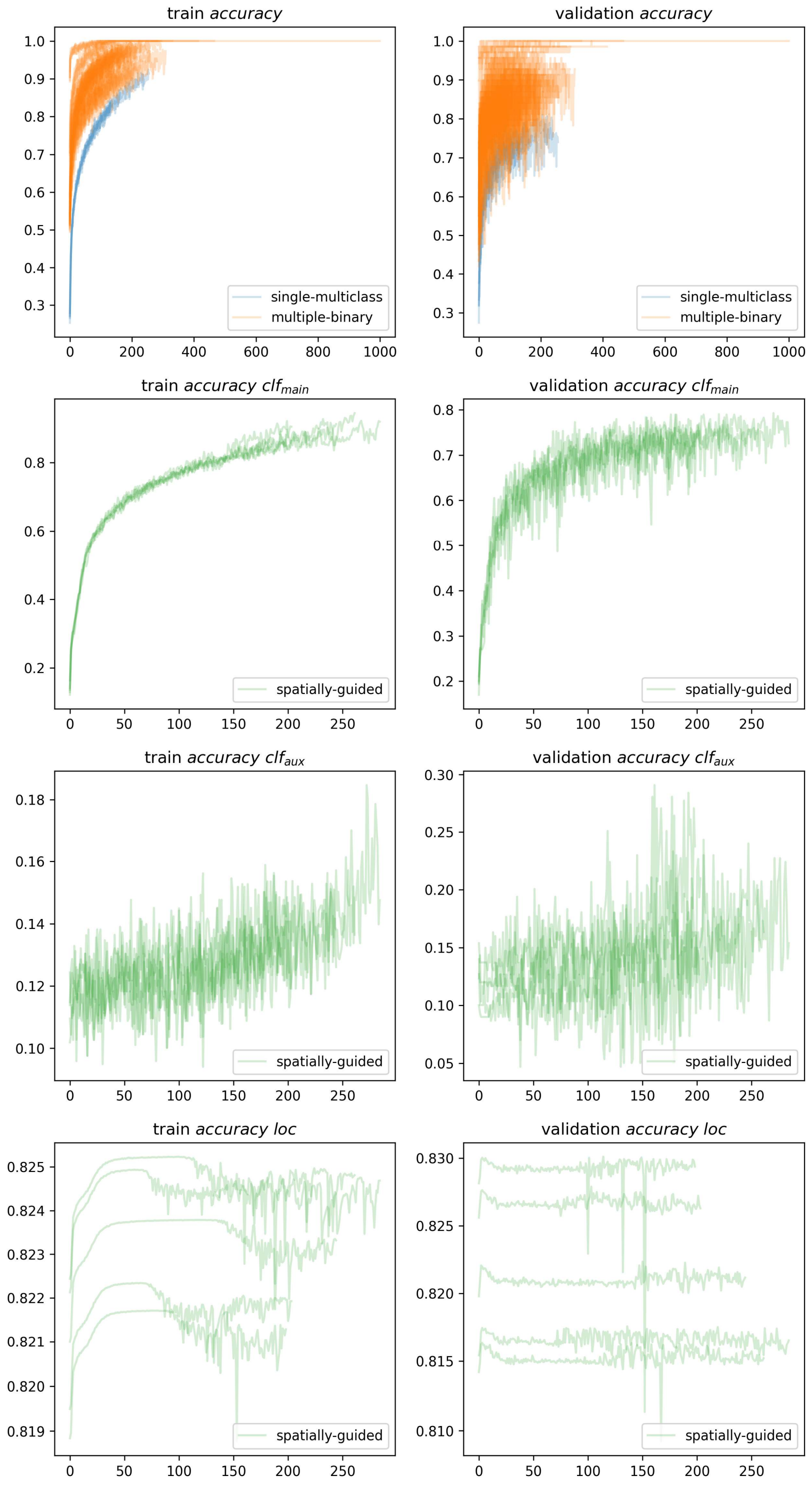

Supplementary Figure S1 (continued). Model training metrics (accuracy) from cross validation of all classifiers.
