# Supplementary material for: A Spatially Guided Machine-Learning Method to Classify and Quantify Glomerular Patterns of Injury in Histology Images
Source: J Imaging. 2023 Oct 11;9(10):220. doi: 10.3390/jimaging9100220 (PMC10607091; doi:10.3390/jimaging9100220)
Supplement: Supplementary file 1 [file jimaging-09-00220-s001.zip › Supplementary Figure S2.pdf]

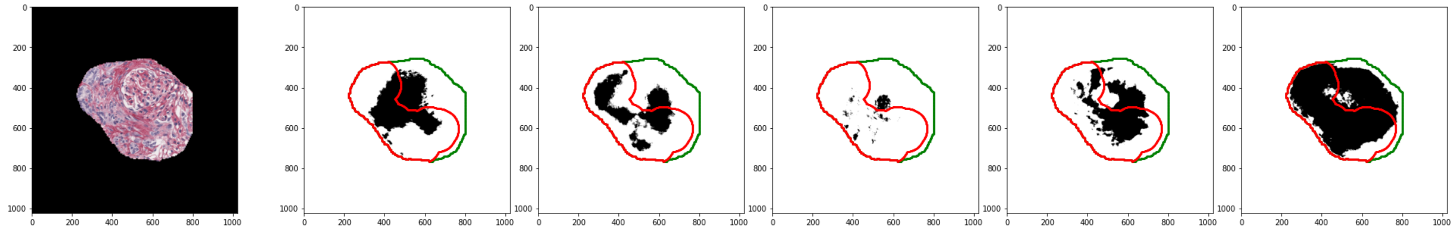

predicted area, %:	31.000	24.263	3.293	28.720	73.154
annotated area, %:	64.358	64.358	64.358	64.358	64.358
true label:	Crescentic	Crescentic	Crescentic	Crescentic	Crescentic
predicted label:	Crescentic	Crescentic	Crescentic	Crescentic	Crescentic
id: 001	CV fold 1	CV fold 2	CV fold 3	CV fold 4	CV fold 5

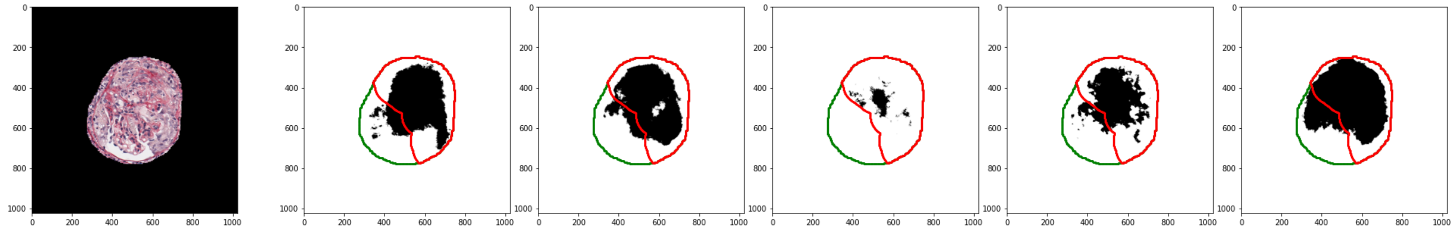

predicted area, %:	45.287	48.746	5.302	33.157	61.616
annotated area, %:	67.326	67.326	67.326	67.326	67.326
true label:	Crescentic	Crescentic	Crescentic	Crescentic	Crescentic
predicted label:	Crescentic	Crescentic	Crescentic	Crescentic	Crescentic
id: 002	CV fold 1	CV fold 2	CV fold 3	CV fold 4	CV fold 5

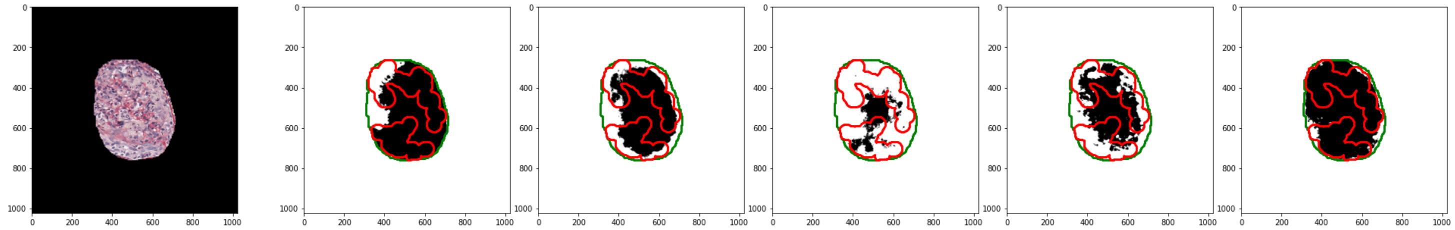

predicted area, %:	79.009	62.369	17.506	50.344	87.049
annotated area, %:	58.806	58.806	58.806	58.806	58.806
true label:	Crescentic	Crescentic	Crescentic	Crescentic	Crescentic
predicted label:	Crescentic	Crescentic	Crescentic	Crescentic	Crescentic
id: 003	CV fold 1	CV fold 2	CV fold 3	CV fold 4	CV fold 5

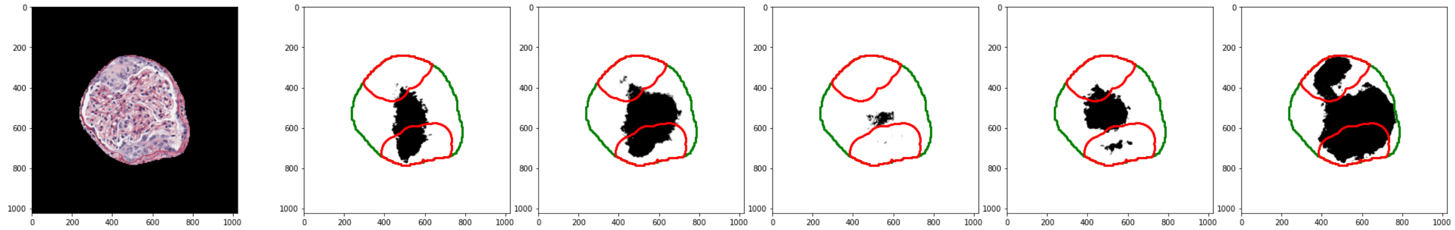

predicted area, %:	19.632	30.867	2.125	16.988	56.714
annotated area, %:	45.840	45.840	45.840	45.840	45.840
true label:	Crescentic	Crescentic	Crescentic	Crescentic	Crescentic
predicted label:	Crescentic	Crescentic	Crescentic	Crescentic	Crescentic
id: 004	CV fold 1	CV fold 2	CV fold 3	CV fold 4	CV fold 5

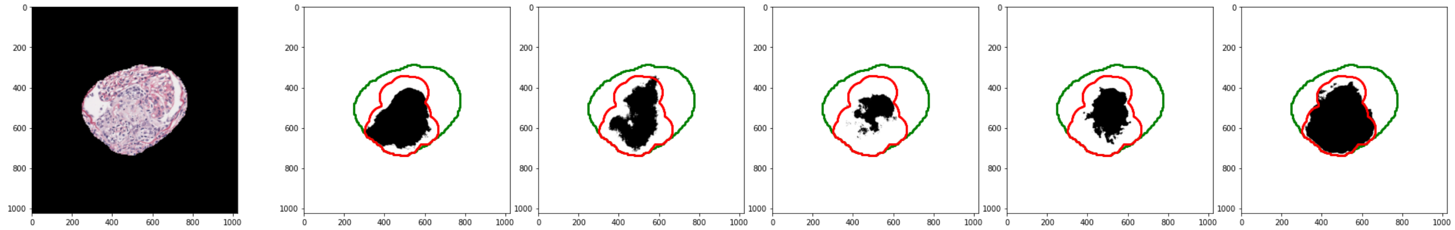

predicted area, %:	36.658	28.462	12.797	21.170	49.037
annotated area, %:	57.633	57.633	57.633	57.633	57.633
true label:	Crescentic	Crescentic	Crescentic	Crescentic	Crescentic
predicted label:	Crescentic	FSGS	Crescentic	Crescentic	Endocapillary
id: 005	CV fold 1	CV fold 2	CV fold 3	CV fold 4	CV fold 5

Supplementary Figure S2. Comparison of pattern quantification results per glomerular basis.

Red contour: annotated area @ 0.5 threshold **Green contour:** total area **Black fill:** predicted area @ 0.5 threshold

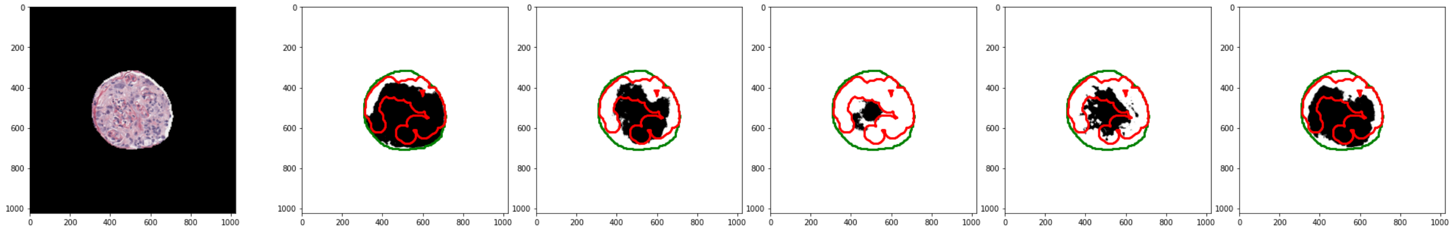

predicted area, %:	77.754	46.414	9.670	28.815	59.302
annotated area, %:	61.022	61.022	61.022	61.022	61.022
true label:	Crescentic	Crescentic	Crescentic	Crescentic	Crescentic
predicted label:	Crescentic	Crescentic	Crescentic	Crescentic	Crescentic
id: 006	CV fold 1	CV fold 2	CV fold 3	CV fold 4	CV fold 5

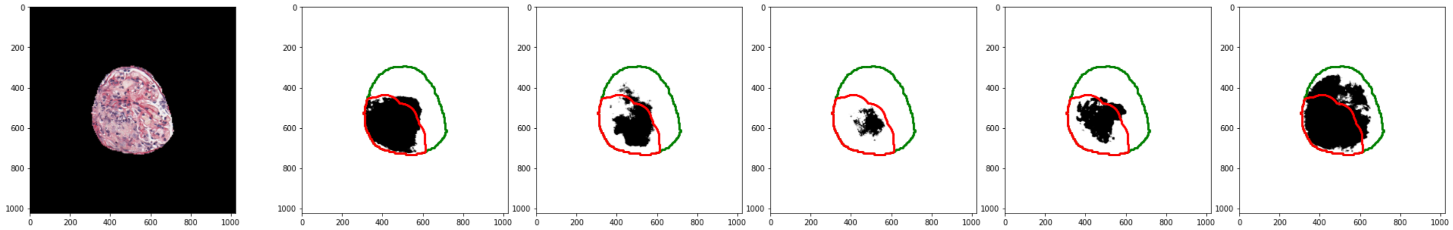

predicted area, %:	45.255	24.957	8.064	23.102	65.962
annotated area, %:	48.779	48.779	48.779	48.779	48.779
true label:	Crescentic	Crescentic	Crescentic	Crescentic	Crescentic
predicted label:	Crescentic	Mesangioproliferative	Crescentic	Crescentic	Crescentic
id: 007	CV fold 1	CV fold 2	CV fold 3	CV fold 4	CV fold 5

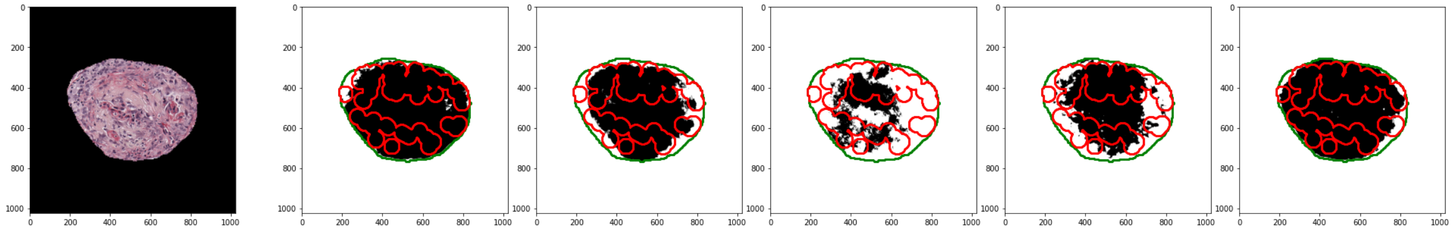

predicted area, %:	91.415	72.830	31.524	58.157	89.591
annotated area, %:	58.509	58.509	58.509	58.509	58.509
true label:	Crescentic	Crescentic	Crescentic	Crescentic	Crescentic
predicted label:	Crescentic	Crescentic	Crescentic	Crescentic	Crescentic
id: 008	CV fold 1	CV fold 2	CV fold 3	CV fold 4	CV fold 5

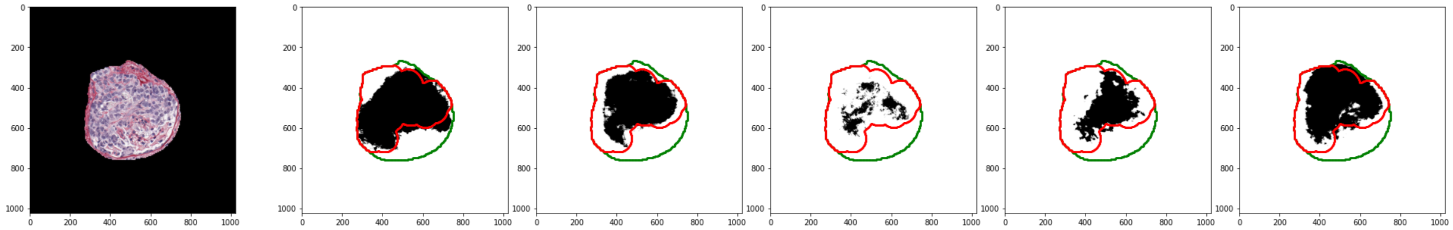

predicted area, %:	65.182	47.378	11.445	29.678	54.595
annotated area, %:	72.932	72.932	72.932	72.932	72.932
true label:	Crescentic	Crescentic	Crescentic	Crescentic	Crescentic
predicted label:	Crescentic	Crescentic	Crescentic	Crescentic	Crescentic
id: 009	CV fold 1	CV fold 2	CV fold 3	CV fold 4	CV fold 5

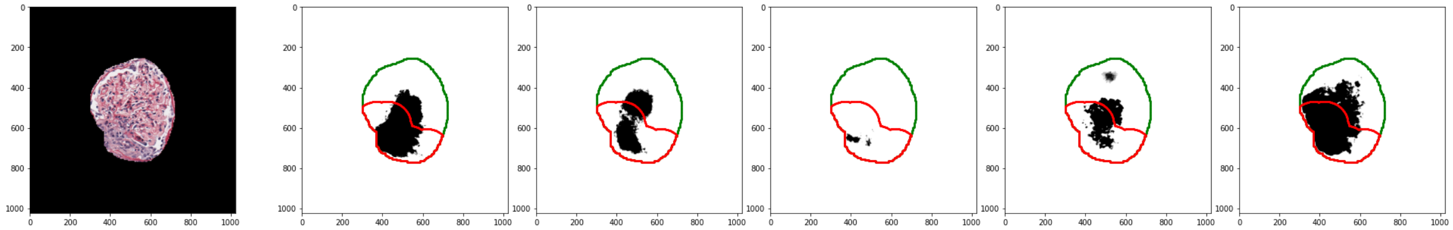

predicted area, %:	32.026	20.441	1.395	17.915	46.112
annotated area, %:	43.066	43.066	43.066	43.066	43.066
true label:	Crescentic	Crescentic	Crescentic	Crescentic	Crescentic
predicted label:	Crescentic	Crescentic	Crescentic	Crescentic	Crescentic
id: 010	CV fold 1	CV fold 2	CV fold 3	CV fold 4	CV fold 5

Supplementary Figure S2 (continued). Comparison of pattern quantification results per glomerular basis.

Red contour: annotated area @ 0.5 threshold **Green contour:** total area **Black fill:** predicted area @ 0.5 threshold

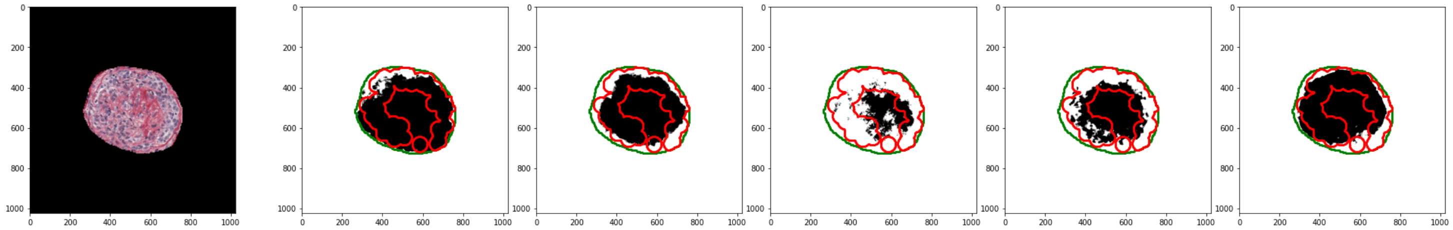

predicted area, %:	82.063	66.372	24.085	48.322	73.500
annotated area, %:	61.552	61.552	61.552	61.552	61.552
true label:	Crescentic	Crescentic	Crescentic	Crescentic	Crescentic
predicted label:	Crescentic	Crescentic	Crescentic	Crescentic	Crescentic
id: 011	CV fold 1	CV fold 2	CV fold 3	CV fold 4	CV fold 5

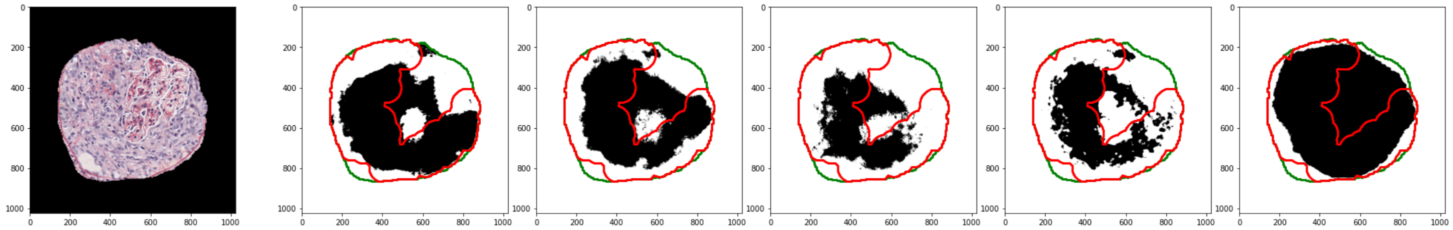

predicted area, %:	57.599	54.386	33.137	37.638	81.531
annotated area, %:	72.429	72.429	72.429	72.429	72.429
true label:	Crescentic	Crescentic	Crescentic	Crescentic	Crescentic
predicted label:	Crescentic	Crescentic	Crescentic	Crescentic	Crescentic
id: 012	CV fold 1	CV fold 2	CV fold 3	CV fold 4	CV fold 5

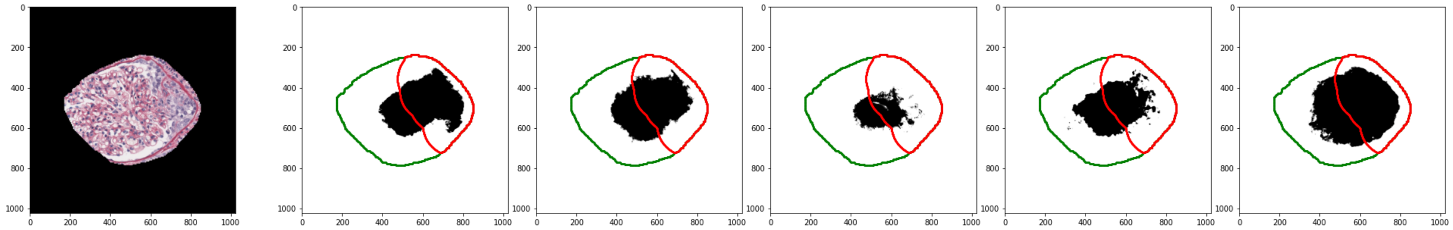

predicted area, %:	33.622	34.500	13.767	27.780	48.455
annotated area, %:	44.108	44.108	44.108	44.108	44.108
true label:	Crescentic	Crescentic	Crescentic	Crescentic	Crescentic
predicted label:	Hypertrophy	Hypertrophy	Hypertrophy	Hypertrophy	Hypertrophy
id: 013	CV fold 1	CV fold 2	CV fold 3	CV fold 4	CV fold 5

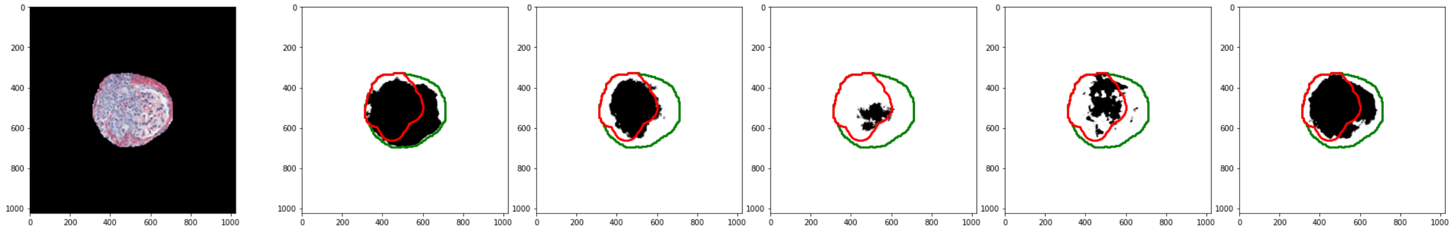

predicted area, %:	79.729	44.935	10.564	25.177	64.240
annotated area, %:	58.734	58.734	58.734	58.734	58.734
true label:	Crescentic	Crescentic	Crescentic	Crescentic	Crescentic
predicted label:	Crescentic	Sclerosed	Crescentic	Sclerosed	Crescentic
id: 014	CV fold 1	CV fold 2	CV fold 3	CV fold 4	CV fold 5

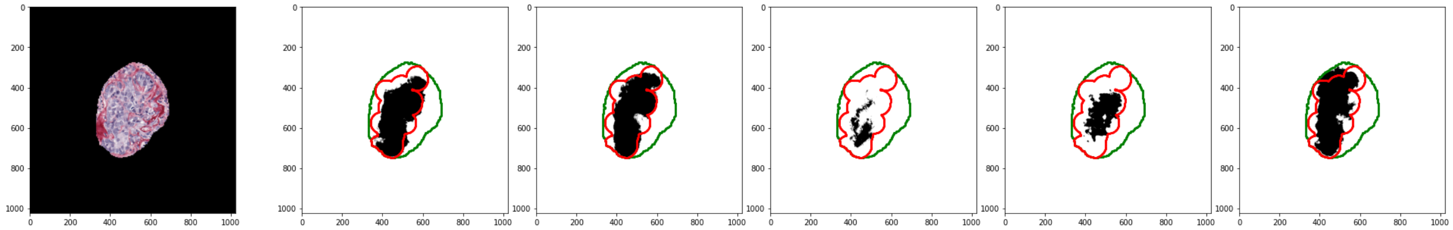

predicted area, %:	47.200	47.885	8.687	20.581	48.464
annotated area, %:	62.381	62.381	62.381	62.381	62.381
true label:	Crescentic	Crescentic	Crescentic	Crescentic	Crescentic
predicted label:	Crescentic	Crescentic	Crescentic	Crescentic	Crescentic
id: 015	CV fold 1	CV fold 2	CV fold 3	CV fold 4	CV fold 5

Supplementary Figure S2 (continued). Comparison of pattern quantification results per glomerular basis.

Red contour: annotated area @ 0.5 threshold **Green contour:** total area **Black fill:** predicted area @ 0.5 threshold

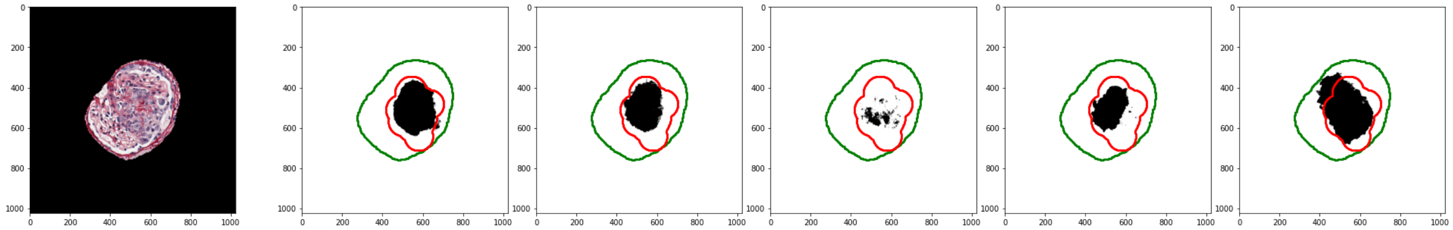

predicted area, %:	26.545	21.248	4.414	16.612	36.466
annotated area, %:	42.383	42.383	42.383	42.383	42.383
true label:	Crescentic	Crescentic	Crescentic	Crescentic	Crescentic
predicted label:	Mesangioproliferative	Mesangioproliferative	Mesangioproliferative	FSGS	Mesangioproliferative
id: 016	CV fold 1	CV fold 2	CV fold 3	CV fold 4	CV fold 5

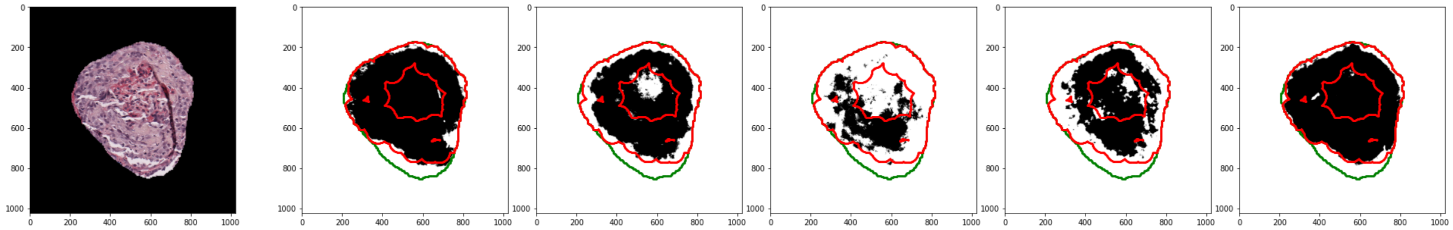

predicted area, %:	82.813	65.255	26.370	52.380	82.094
annotated area, %:	71.708	71.708	71.708	71.708	71.708
true label:	Crescentic	Crescentic	Crescentic	Crescentic	Crescentic
predicted label:	Crescentic	Crescentic	Crescentic	Crescentic	Crescentic
id: 017	CV fold 1	CV fold 2	CV fold 3	CV fold 4	CV fold 5

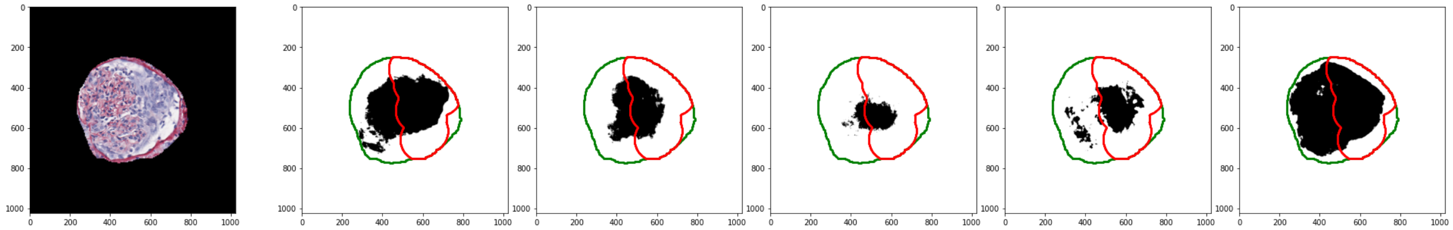

predicted area, %:	45.750	28.568	11.278	19.979	68.511
annotated area, %:	51.876	51.876	51.876	51.876	51.876
true label:	Crescentic	Crescentic	Crescentic	Crescentic	Crescentic
predicted label:	Crescentic	Crescentic	Crescentic	Crescentic	Crescentic
id: 018	CV fold 1	CV fold 2	CV fold 3	CV fold 4	CV fold 5

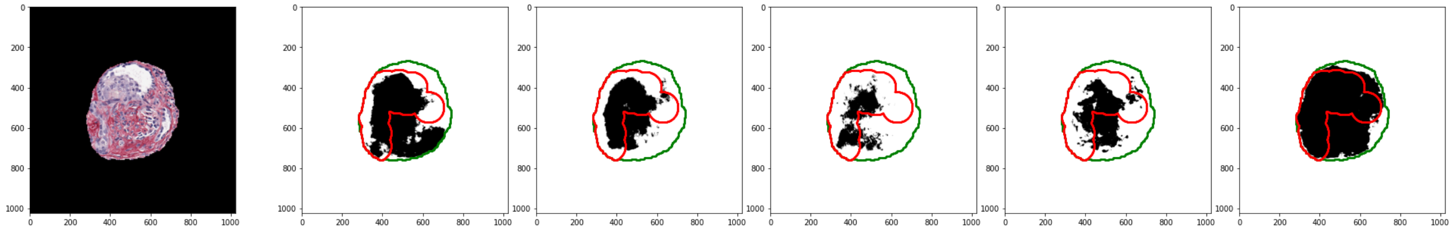

predicted area, %:	56.448	38.494	19.995	31.459	83.938
annotated area, %:	58.183	58.183	58.183	58.183	58.183
true label:	Crescentic	Crescentic	Crescentic	Crescentic	Crescentic
predicted label:	Crescentic	Crescentic	Crescentic	Crescentic	Crescentic
id: 019	CV fold 1	CV fold 2	CV fold 3	CV fold 4	CV fold 5

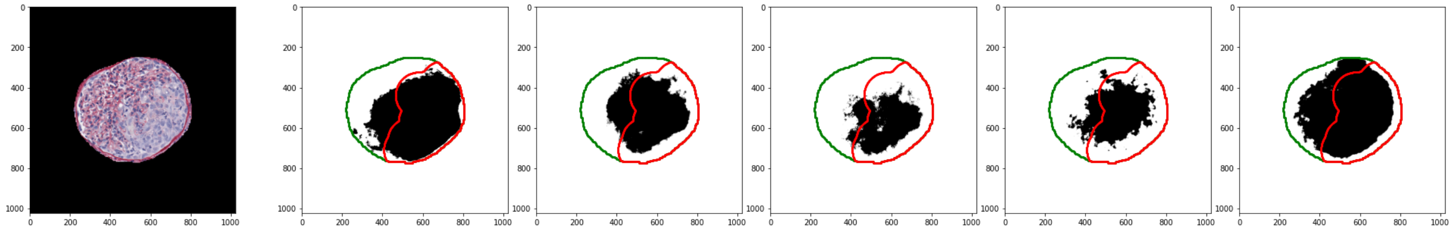

predicted area, %:	63.110	47.194	31.603	35.344	71.099
annotated area, %:	57.675	57.675	57.675	57.675	57.675
true label:	Crescentic	Crescentic	Crescentic	Crescentic	Crescentic
predicted label:	Crescentic	Crescentic	Crescentic	Crescentic	Crescentic
id: 020	CV fold 1	CV fold 2	CV fold 3	CV fold 4	CV fold 5

Supplementary Figure S2 (continued). Comparison of pattern quantification results per glomerular basis.

Red contour: annotated area @ 0.5 threshold **Green contour:** total area **Black fill:** predicted area @ 0.5 threshold

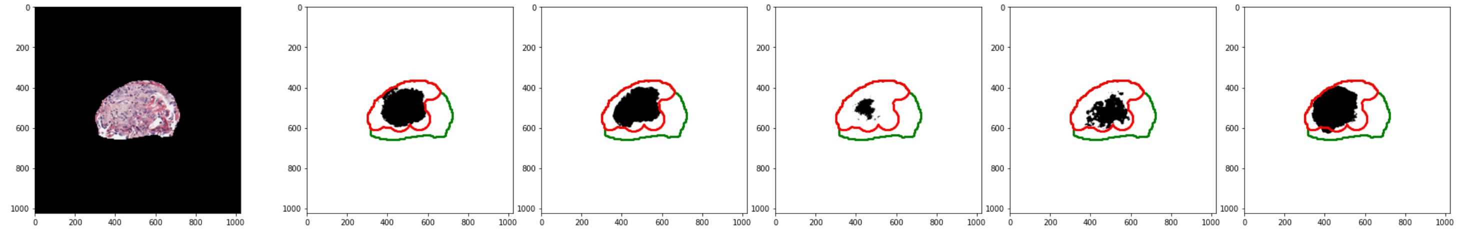

predicted area, %:	35.930	35.379	6.637	21.709	39.610
annotated area, %:	64.849	64.849	64.849	64.849	64.849
true label:	Crescentic	Crescentic	Crescentic	Crescentic	Crescentic
predicted label:	Crescentic	Mesangioproliferative	Crescentic	Crescentic	Crescentic
id: 021	CV fold 1	CV fold 2	CV fold 3	CV fold 4	CV fold 5

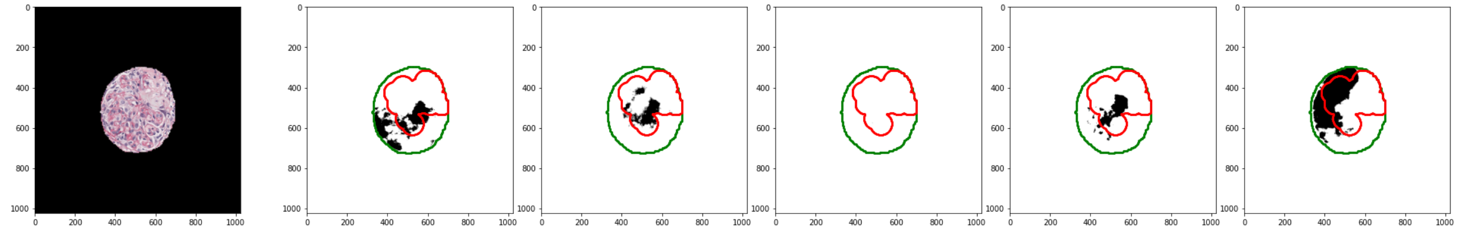

predicted area, %:	18.775	10.196	0.002	9.135	34.357
annotated area, %:	51.796	51.796	51.796	51.796	51.796
true label:	Crescentic	Crescentic	Crescentic	Crescentic	Crescentic
predicted label:	Crescentic	Crescentic	Crescentic	Crescentic	Crescentic
id: 022	CV fold 1	CV fold 2	CV fold 3	CV fold 4	CV fold 5

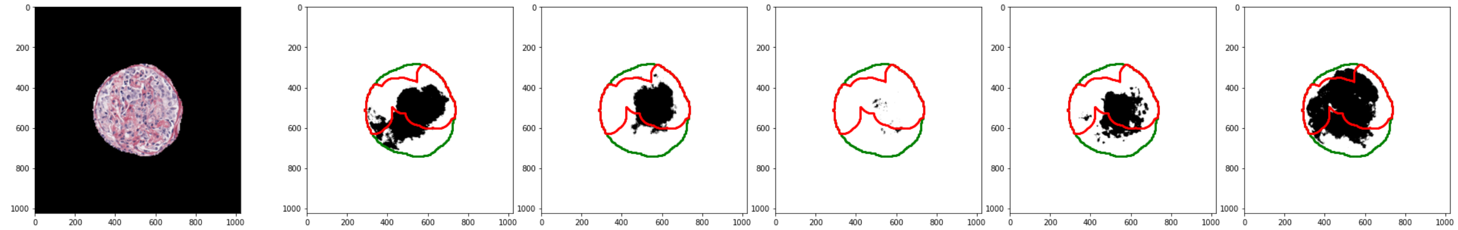

predicted area, %:	40.295	24.572	0.931	24.628	58.430
annotated area, %:	61.565	61.565	61.565	61.565	61.565
true label:	Crescentic	Crescentic	Crescentic	Crescentic	Crescentic
predicted label:	Crescentic	Crescentic	Crescentic	Crescentic	Crescentic
id: 023	CV fold 1	CV fold 2	CV fold 3	CV fold 4	CV fold 5

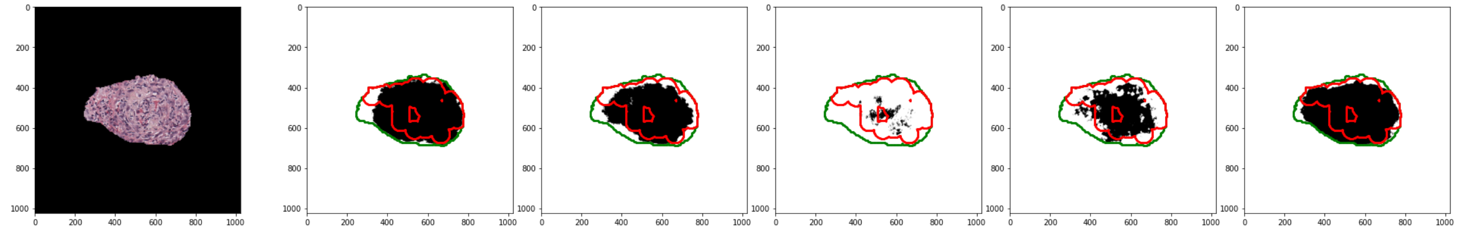

predicted area, %:	83.485	69.203	6.120	46.662	84.817
annotated area, %:	71.469	71.469	71.469	71.469	71.469
true label:	Crescentic	Crescentic	Crescentic	Crescentic	Crescentic
predicted label:	Crescentic	Crescentic	Crescentic	Crescentic	Crescentic
id: 024	CV fold 1	CV fold 2	CV fold 3	CV fold 4	CV fold 5

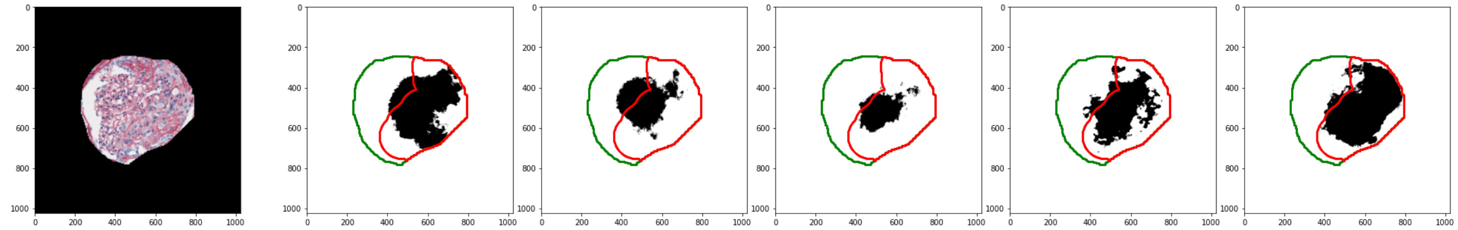

predicted area, %:	39.408	23.466	13.858	31.883	43.883
annotated area, %:	59.612	59.612	59.612	59.612	59.612
true label:	Crescentic	Crescentic	Crescentic	Crescentic	Crescentic
predicted label:	Crescentic	Hypertrophy	FSGS	FSGS	Crescentic
id: 025	CV fold 1	CV fold 2	CV fold 3	CV fold 4	CV fold 5

Supplementary Figure S2 (continued). Comparison of pattern quantification results per glomerular basis.

Red contour: annotated area @ 0.5 threshold **Green contour:** total area **Black fill:** predicted area @ 0.5 threshold

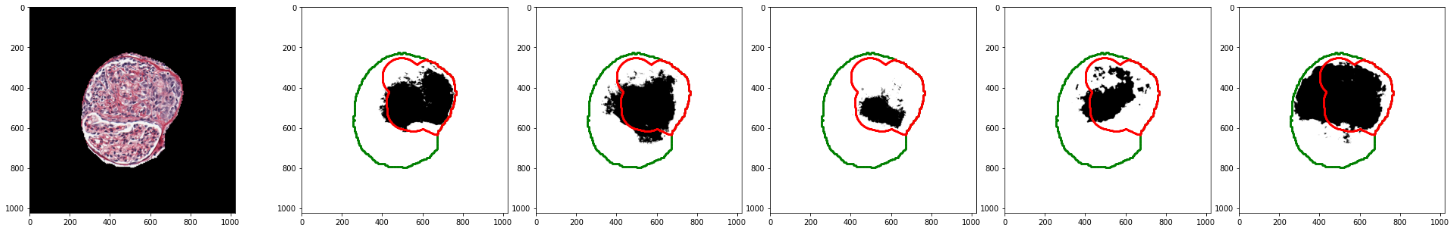

predicted area, %:	33.869	35.384	10.754	19.911	52.487
annotated area, %:	48.999	48.999	48.999	48.999	48.999
true label:	Crescentic	Crescentic	Crescentic	Crescentic	Crescentic
predicted label:	Endocapillary	Endocapillary	Endocapillary	Membranoproliferative	Endocapillary
id: 026	CV fold 1	CV fold 2	CV fold 3	CV fold 4	CV fold 5

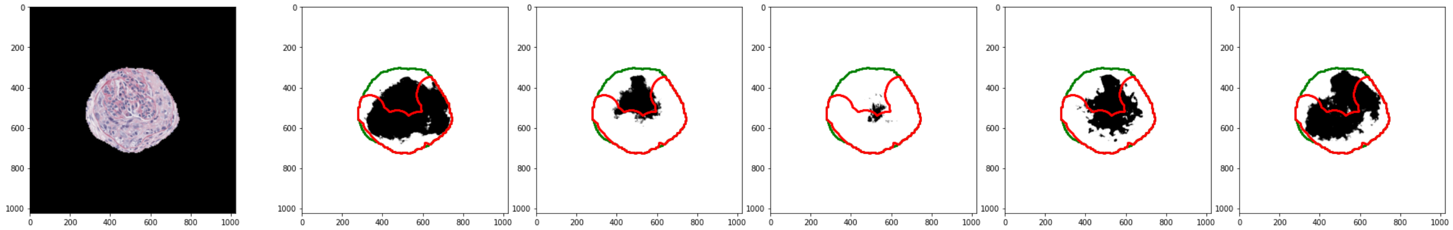

predicted area, %:	62.468	20.341	3.112	33.412	53.265
annotated area, %:	66.588	66.588	66.588	66.588	66.588
true label:	Crescentic	Crescentic	Crescentic	Crescentic	Crescentic
predicted label:	Crescentic	Crescentic	Crescentic	Crescentic	Crescentic
id: 027	CV fold 1	CV fold 2	CV fold 3	CV fold 4	CV fold 5

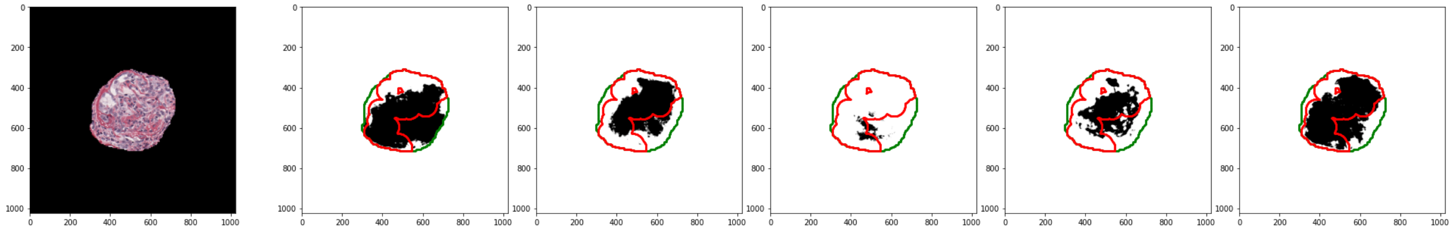

predicted area, %:	65.927	46.896	4.944	33.900	64.954
annotated area, %:	71.582	71.582	71.582	71.582	71.582
true label:	Crescentic	Crescentic	Crescentic	Crescentic	Crescentic
predicted label:	Crescentic	Endocapillary	Endocapillary	Crescentic	Endocapillary
id: 028	CV fold 1	CV fold 2	CV fold 3	CV fold 4	CV fold 5

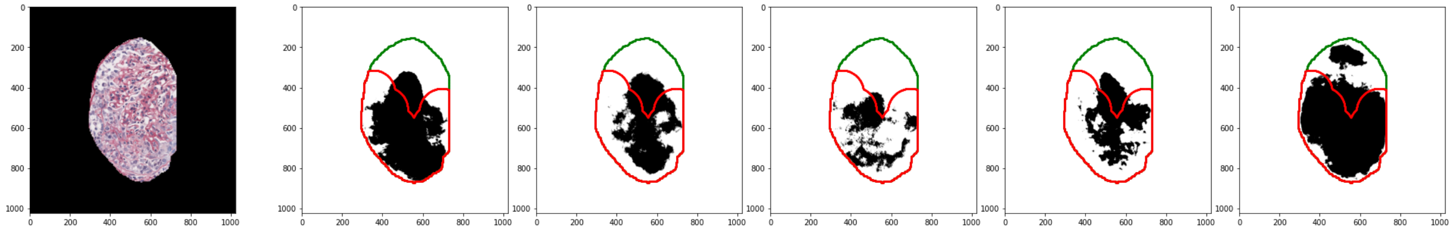

predicted area, %:	54.368	37.930	20.950	30.148	70.122
annotated area, %:	67.952	67.952	67.952	67.952	67.952
true label:	Crescentic	Crescentic	Crescentic	Crescentic	Crescentic
predicted label:	Crescentic	Crescentic	Crescentic	Crescentic	Crescentic
id: 029	CV fold 1	CV fold 2	CV fold 3	CV fold 4	CV fold 5

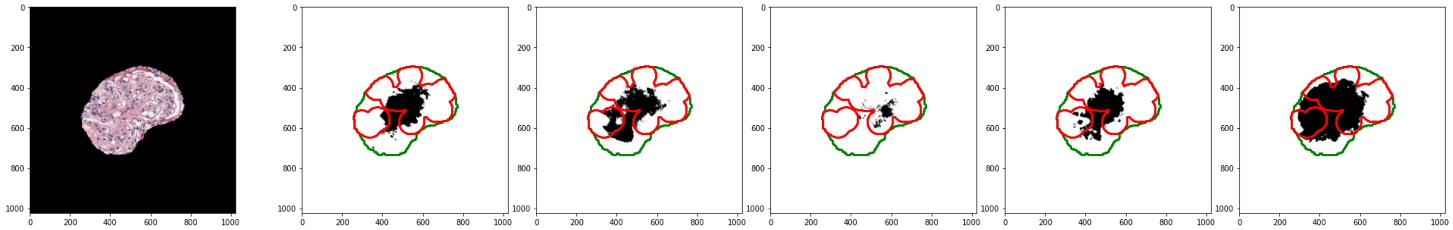

predicted area, %:	22.464	27.671	4.025	22.311	51.509
annotated area, %:	67.459	67.459	67.459	67.459	67.459
true label:	Endocapillary	Endocapillary	Endocapillary	Endocapillary	Endocapillary
predicted label:	Endocapillary	Endocapillary	Endocapillary	Endocapillary	Endocapillary
id: 030	CV fold 1	CV fold 2	CV fold 3	CV fold 4	CV fold 5

Supplementary Figure S2 (continued). Comparison of pattern quantification results per glomerular basis.

Red contour: annotated area @ 0.5 threshold **Green contour:** total area **Black fill:** predicted area @ 0.5 threshold

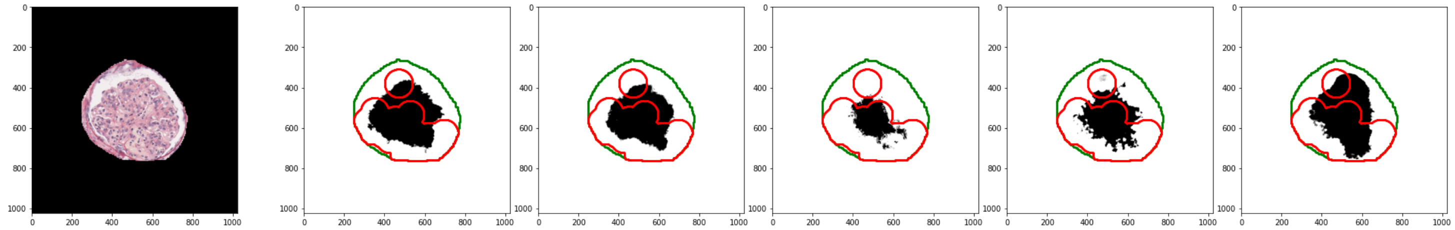

predicted area, %:	40.967	36.378	15.910	28.516	42.843
annotated area, %:	62.267	62.267	62.267	62.267	62.267
true label:	Endocapillary	Endocapillary	Endocapillary	Endocapillary	Endocapillary
predicted label:	Endocapillary	Endocapillary	Endocapillary	Endocapillary	Endocapillary
id: 031	CV fold 1	CV fold 2	CV fold 3	CV fold 4	CV fold 5

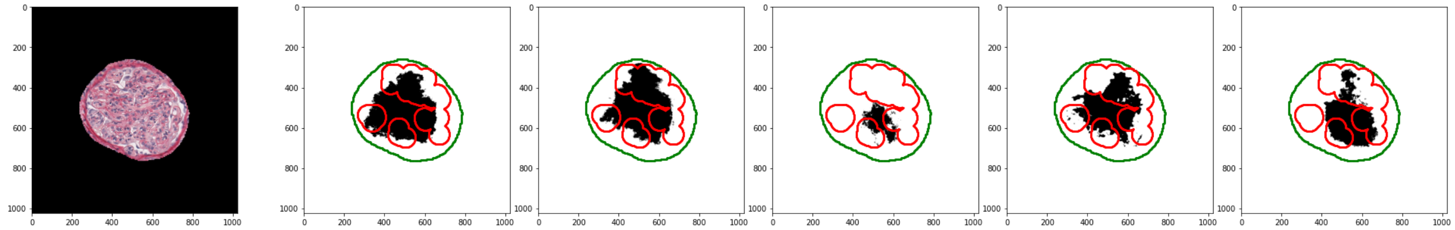

predicted area, %:	41.432	40.005	8.236	31.708	26.800
annotated area, %:	50.388	50.388	50.388	50.388	50.388
true label:	Endocapillary	Endocapillary	Endocapillary	Endocapillary	Endocapillary
predicted label:	Crescentic	Crescentic	Crescentic	Crescentic	Crescentic
id: 032	CV fold 1	CV fold 2	CV fold 3	CV fold 4	CV fold 5

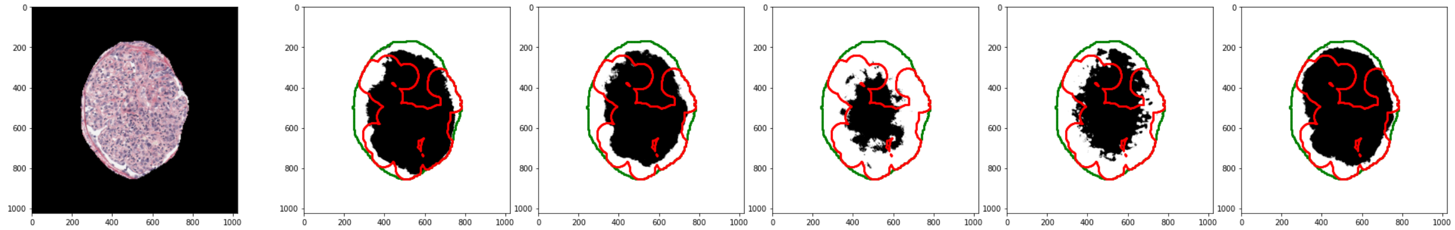

predicted area, %:	72.605	63.236	27.799	47.431	73.035
annotated area, %:	69.920	69.920	69.920	69.920	69.920
true label:	Endocapillary	Endocapillary	Endocapillary	Endocapillary	Endocapillary
predicted label:	Endocapillary	Endocapillary	Endocapillary	Endocapillary	Endocapillary
id: 033	CV fold 1	CV fold 2	CV fold 3	CV fold 4	CV fold 5

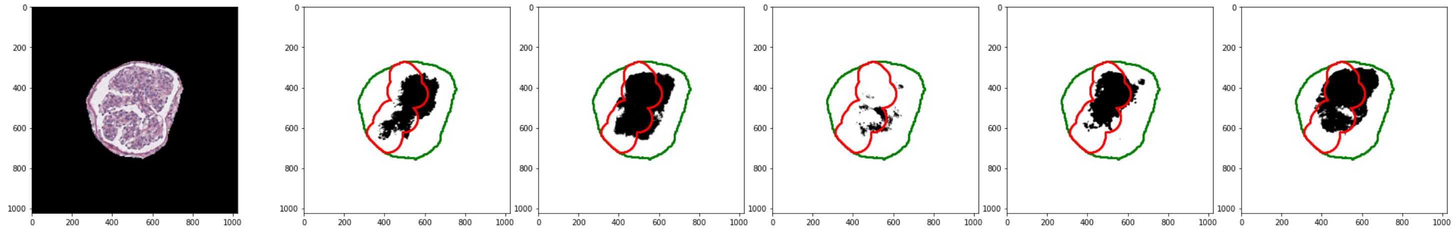

predicted area, %:	26.873	38.946	5.830	25.121	38.492
annotated area, %:	43.151	43.151	43.151	43.151	43.151
true label:	Endocapillary	Endocapillary	Endocapillary	Endocapillary	Endocapillary
predicted label:	Endocapillary	Endocapillary	Endocapillary	Endocapillary	Endocapillary
id: 034	CV fold 1	CV fold 2	CV fold 3	CV fold 4	CV fold 5

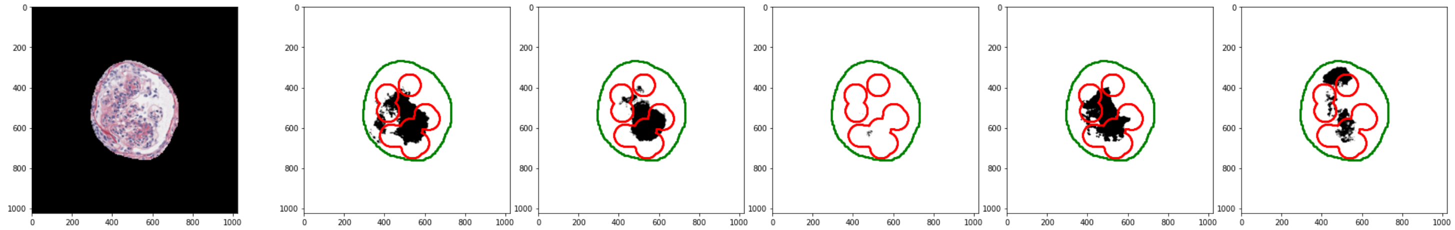

predicted area, %:	22.740	16.920	0.174	20.573	12.812
annotated area, %:	41.399	41.399	41.399	41.399	41.399
true label:	Endocapillary	Endocapillary	Endocapillary	Endocapillary	Endocapillary
predicted label:	Crescentic	Crescentic	Crescentic	Crescentic	Endocapillary
id: 035	CV fold 1	CV fold 2	CV fold 3	CV fold 4	CV fold 5

Supplementary Figure S2 (continued). Comparison of pattern quantification results per glomerular basis.

Red contour: annotated area @ 0.5 threshold **Green contour:** total area **Black fill:** predicted area @ 0.5 threshold

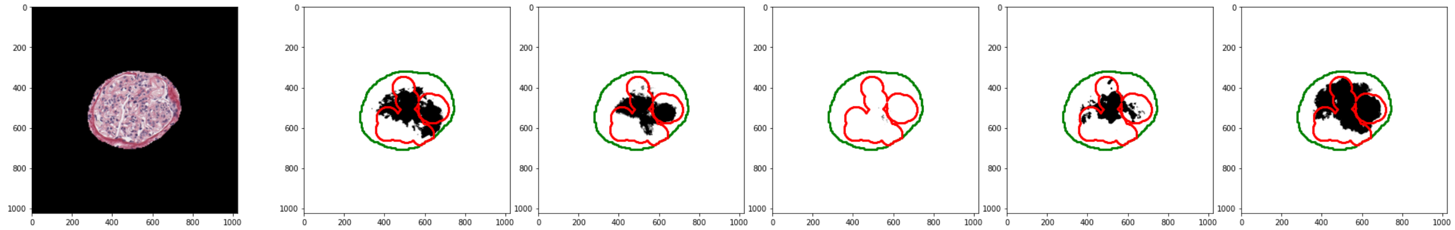

predicted area, %:	31.911	23.193	0.109	16.759	42.966
annotated area, %:	52.228	52.228	52.228	52.228	52.228
true label:	Endocapillary	Endocapillary	Endocapillary	Endocapillary	Endocapillary
predicted label:	FSGS	FSGS	FSGS	FSGS	FSGS
id: 036	CV fold 1	CV fold 2	CV fold 3	CV fold 4	CV fold 5

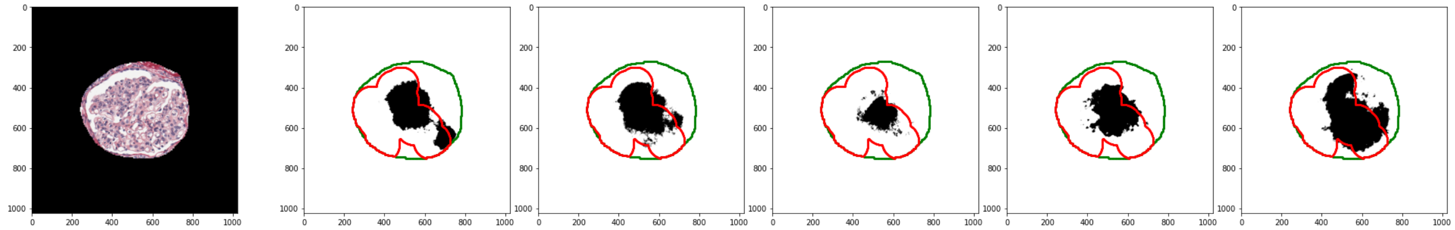

predicted area, %:	24.961	26.676	10.843	22.364	37.733
annotated area, %:	66.543	66.543	66.543	66.543	66.543
true label:	Endocapillary	Endocapillary	Endocapillary	Endocapillary	Endocapillary
predicted label:	Membranoproliferative	Membranoproliferative	Membranoproliferative	Membranoproliferative	Membranoproliferative
id: 037	CV fold 1	CV fold 2	CV fold 3	CV fold 4	CV fold 5

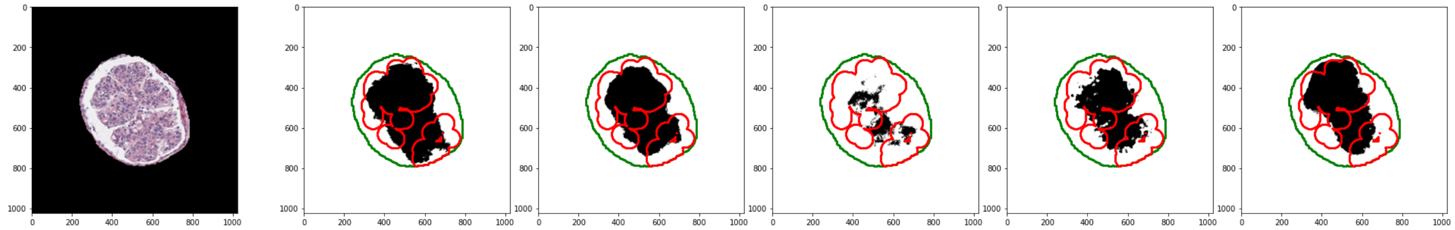

predicted area, %:	53.877	47.403	11.289	36.000	45.563
annotated area, %:	61.460	61.460	61.460	61.460	61.460
true label:	Endocapillary	Endocapillary	Endocapillary	Endocapillary	Endocapillary
predicted label:	Endocapillary	Endocapillary	Endocapillary	Endocapillary	Endocapillary
id: 038	CV fold 1	CV fold 2	CV fold 3	CV fold 4	CV fold 5

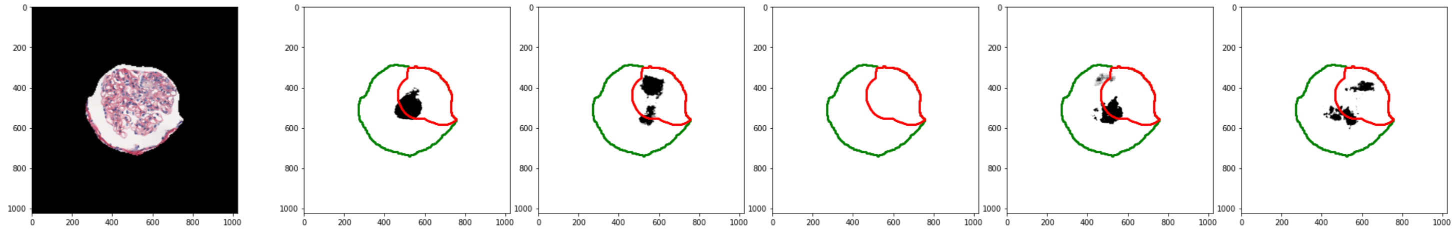

predicted area, %:	8.680	8.333	0.000	7.085	7.235
annotated area, %:	36.677	36.677	36.677	36.677	36.677
true label:	Endocapillary	Endocapillary	Endocapillary	Endocapillary	Endocapillary
predicted label:	Normal	Normal	Normal	Normal	FSGS
id: 039	CV fold 1	CV fold 2	CV fold 3	CV fold 4	CV fold 5

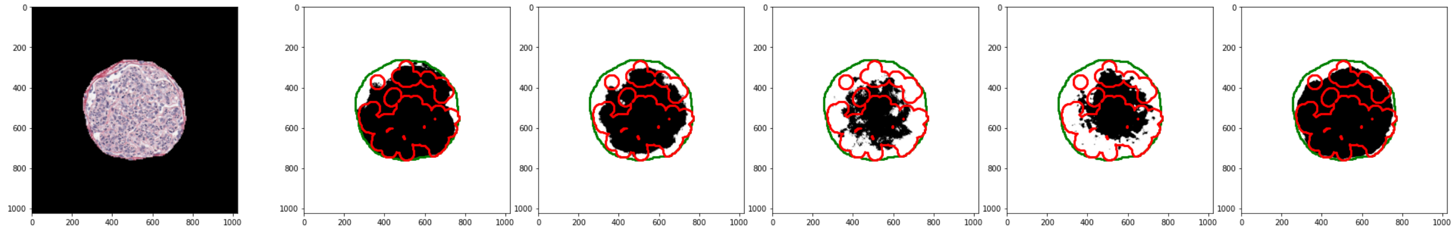

predicted area, %:	84.944	66.000	37.783	39.496	83.222
annotated area, %:	68.605	68.605	68.605	68.605	68.605
true label:	Endocapillary	Endocapillary	Endocapillary	Endocapillary	Endocapillary
predicted label:	Endocapillary	Endocapillary	Endocapillary	Endocapillary	Endocapillary
id: 040	CV fold 1	CV fold 2	CV fold 3	CV fold 4	CV fold 5

Supplementary Figure S2 (continued). Comparison of pattern quantification results per glomerular basis.

Red contour: annotated area @ 0.5 threshold **Green contour:** total area **Black fill:** predicted area @ 0.5 threshold

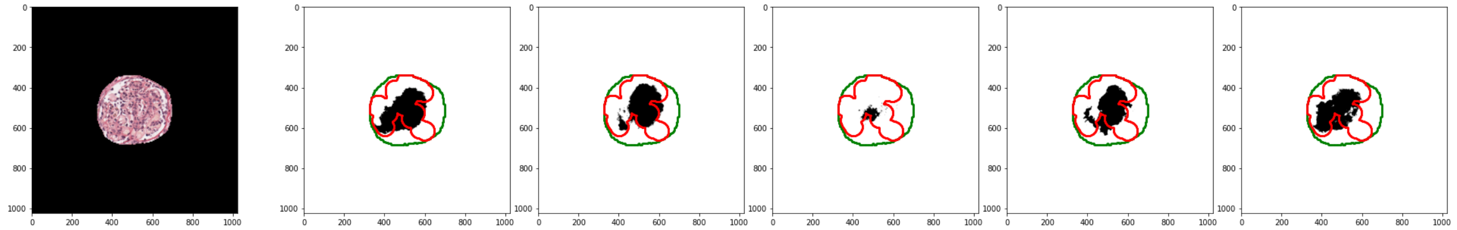

predicted area, %:	34.999	30.026	4.230	27.428	31.109
annotated area, %:	64.139	64.139	64.139	64.139	64.139
true label:	Endocapillary	Endocapillary	Endocapillary	Endocapillary	Endocapillary
predicted label:	Mesangioproliferative	Mesangioproliferative	Endocapillary	Mesangioproliferative	Endocapillary
id: 041	CV fold 1	CV fold 2	CV fold 3	CV fold 4	CV fold 5

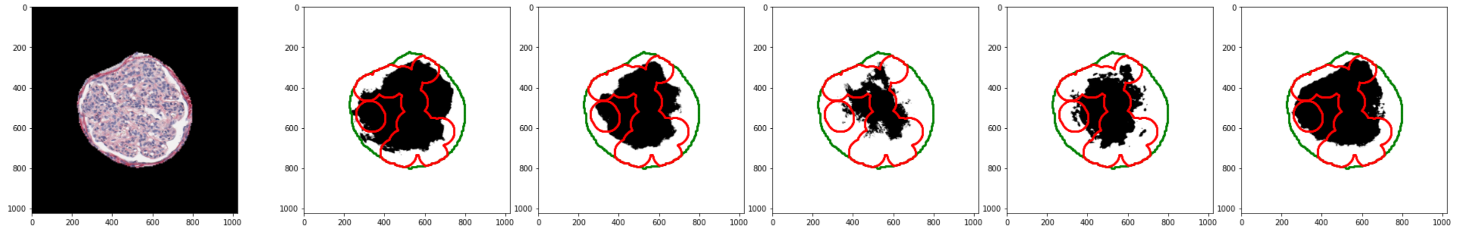

predicted area, %:	63.727	49.201	25.255	36.888	56.557
annotated area, %:	63.285	63.285	63.285	63.285	63.285
true label:	Endocapillary	Endocapillary	Endocapillary	Endocapillary	Endocapillary
predicted label:	Endocapillary	Endocapillary	Endocapillary	Endocapillary	Endocapillary
id: 042	CV fold 1	CV fold 2	CV fold 3	CV fold 4	CV fold 5

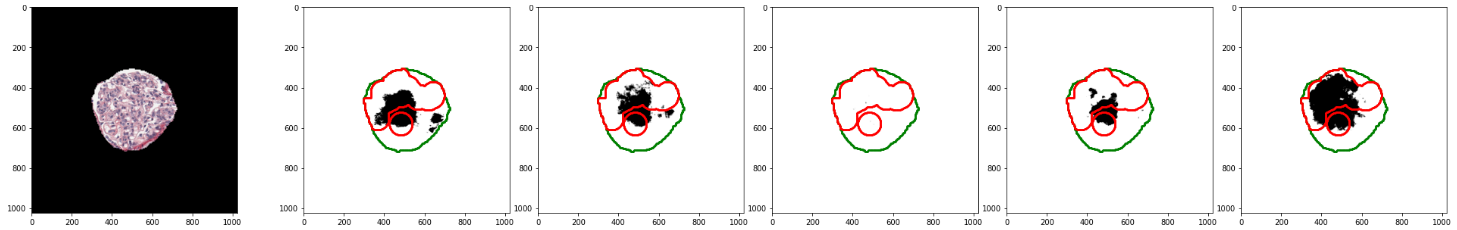

predicted area, %:	24.246	20.811	0.031	12.286	41.143
annotated area, %:	58.949	58.949	58.949	58.949	58.949
true label:	Endocapillary	Endocapillary	Endocapillary	Endocapillary	Endocapillary
predicted label:	Endocapillary	Endocapillary	Endocapillary	Endocapillary	Endocapillary
id: 043	CV fold 1	CV fold 2	CV fold 3	CV fold 4	CV fold 5

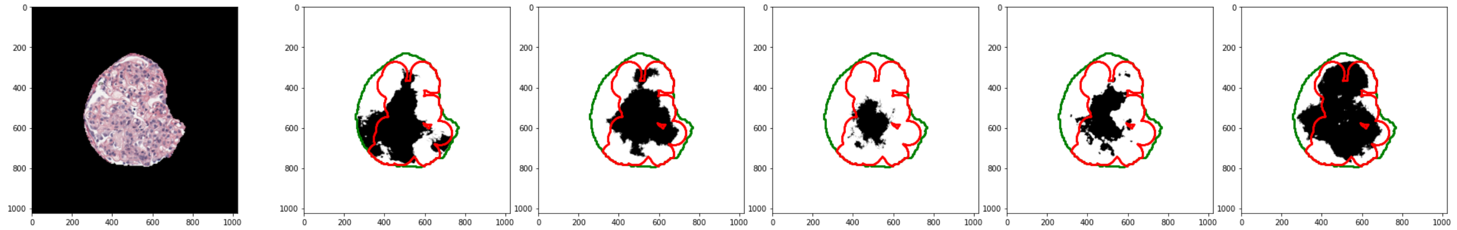

predicted area, %:	43.587	37.252	13.591	21.418	58.718
annotated area, %:	73.312	73.312	73.312	73.312	73.312
true label:	Endocapillary	Endocapillary	Endocapillary	Endocapillary	Endocapillary
predicted label:	Crescentic	Crescentic	Endocapillary	Crescentic	Endocapillary
id: 044	CV fold 1	CV fold 2	CV fold 3	CV fold 4	CV fold 5

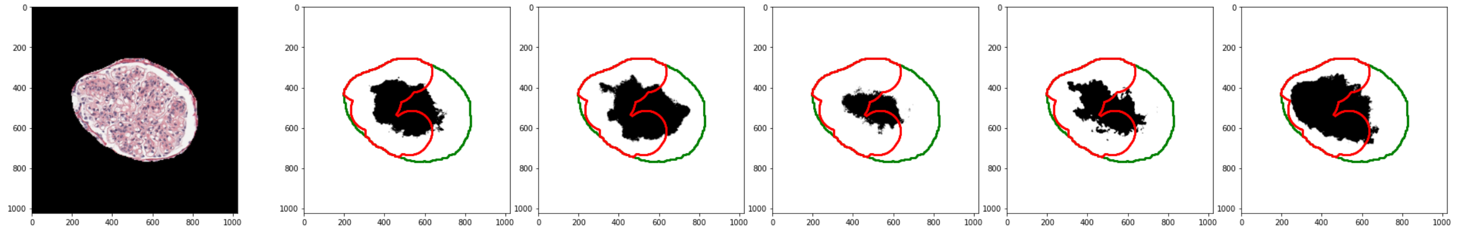

predicted area, %:	29.464	34.987	15.108	24.087	41.507
annotated area, %:	59.937	59.937	59.937	59.937	59.937
true label:	Endocapillary	Endocapillary	Endocapillary	Endocapillary	Endocapillary
predicted label:	Endocapillary	Endocapillary	Membranous	Endocapillary	Endocapillary
id: 045	CV fold 1	CV fold 2	CV fold 3	CV fold 4	CV fold 5

Supplementary Figure S2 (continued). Comparison of pattern quantification results per glomerular basis.

Red contour: annotated area @ 0.5 threshold **Green contour:** total area **Black fill:** predicted area @ 0.5 threshold

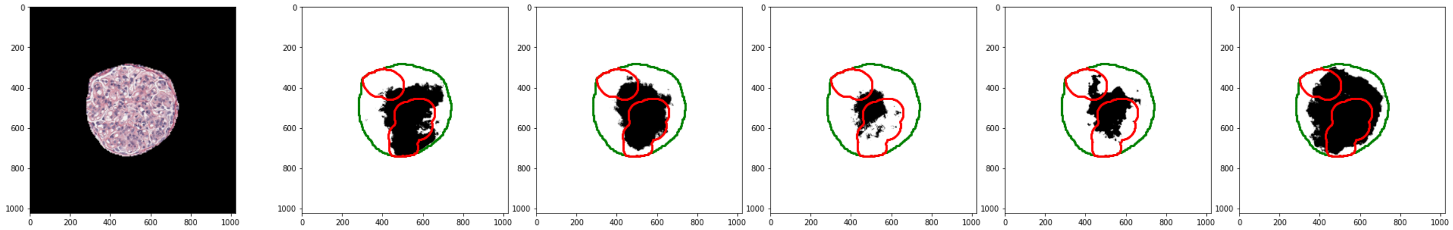

predicted area, %:	45.648	39.889	14.641	24.164	67.572
annotated area, %:	42.728	42.728	42.728	42.728	42.728
true label:	Endocapillary	Endocapillary	Endocapillary	Endocapillary	Endocapillary
predicted label:	Endocapillary	Mesangioproliferative	Membranoproliferative	Membranoproliferative	Endocapillary
id: 046	CV fold 1	CV fold 2	CV fold 3	CV fold 4	CV fold 5

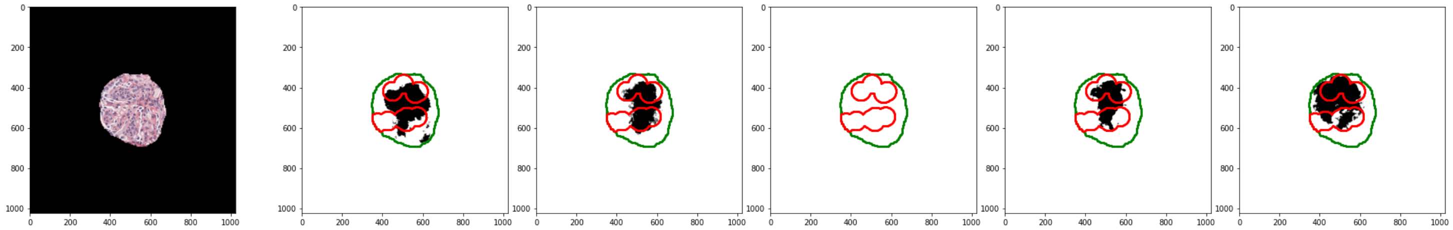

predicted area, %:	38.463	32.249	0.000	24.636	44.630
annotated area, %:	45.929	45.929	45.929	45.929	45.929
true label:	Endocapillary	Endocapillary	Endocapillary	Endocapillary	Endocapillary
predicted label:	Crescentic	Crescentic	Endocapillary	Crescentic	Endocapillary
id: 047	CV fold 1	CV fold 2	CV fold 3	CV fold 4	CV fold 5

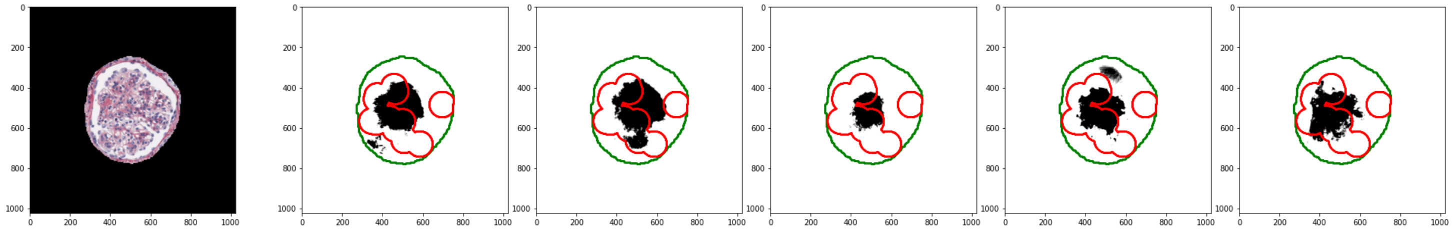

predicted area, %:	23.262	28.195	11.342	20.730	22.684
annotated area, %:	47.349	47.349	47.349	47.349	47.349
true label:	Endocapillary	Endocapillary	Endocapillary	Endocapillary	Endocapillary
predicted label:	Endocapillary	Endocapillary	Endocapillary	Crescentic	Endocapillary
id: 048	CV fold 1	CV fold 2	CV fold 3	CV fold 4	CV fold 5

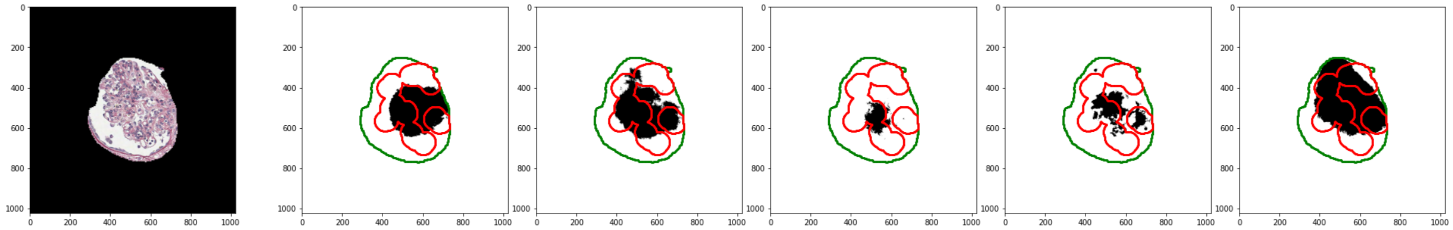

predicted area, %:	33.016	36.149	7.898	14.985	54.850
annotated area, %:	51.679	51.679	51.679	51.679	51.679
true label:	Endocapillary	Endocapillary	Endocapillary	Endocapillary	Endocapillary
predicted label:	Endocapillary	Endocapillary	Endocapillary	Endocapillary	Endocapillary
id: 049	CV fold 1	CV fold 2	CV fold 3	CV fold 4	CV fold 5

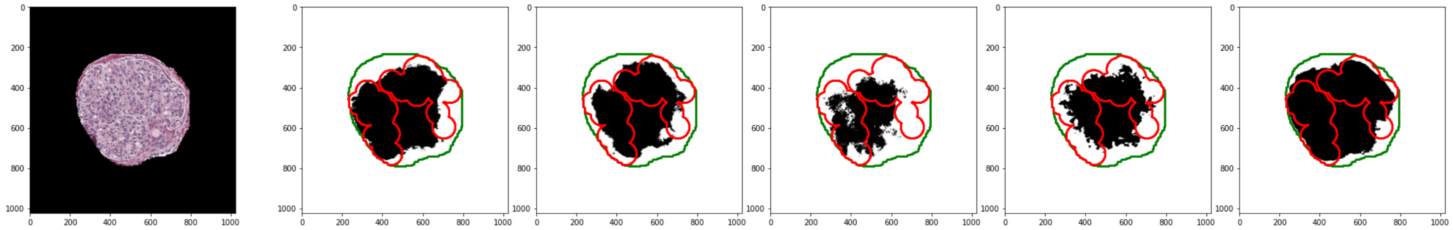

predicted area, %:	62.702	57.621	35.262	46.834	77.098
annotated area, %:	59.178	59.178	59.178	59.178	59.178
true label:	Endocapillary	Endocapillary	Endocapillary	Endocapillary	Endocapillary
predicted label:	Endocapillary	Crescentic	Endocapillary	Endocapillary	Endocapillary
id: 050	CV fold 1	CV fold 2	CV fold 3	CV fold 4	CV fold 5

Supplementary Figure S2 (continued). Comparison of pattern quantification results per glomerular basis.

Red contour: annotated area @ 0.5 threshold **Green contour:** total area **Black fill:** predicted area @ 0.5 threshold

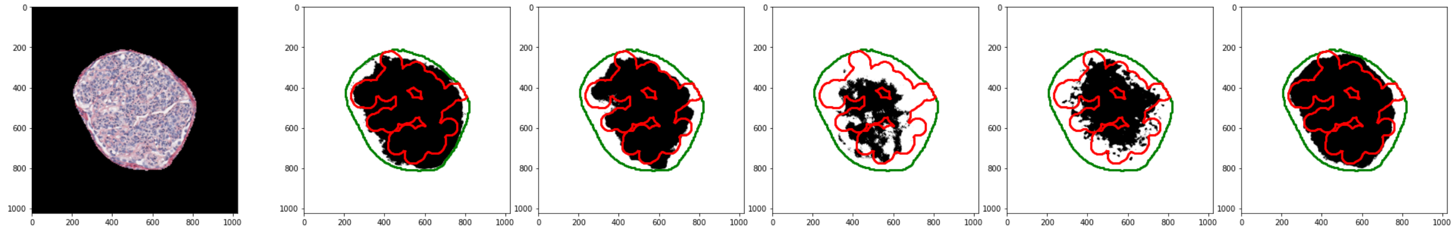

predicted area, %:	81.337	66.642	30.816	44.675	78.541
annotated area, %:	61.509	61.509	61.509	61.509	61.509
true label:	Endocapillary	Endocapillary	Endocapillary	Endocapillary	Endocapillary
predicted label:	Endocapillary	Endocapillary	Endocapillary	Endocapillary	Endocapillary
id: 051	CV fold 1	CV fold 2	CV fold 3	CV fold 4	CV fold 5

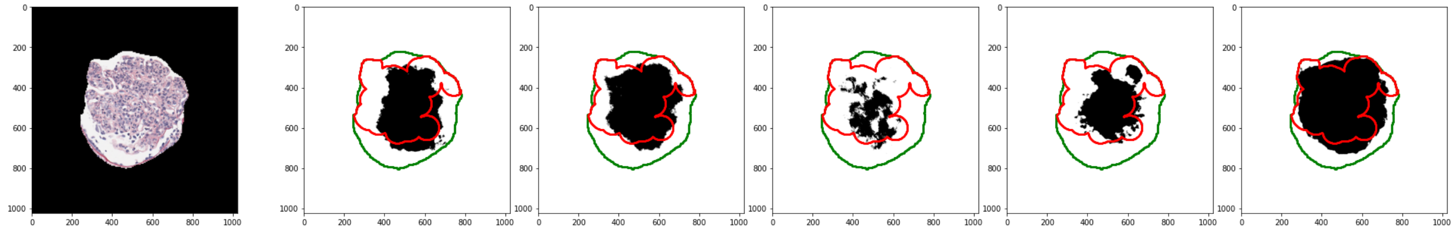

predicted area, %:	45.381	49.929	17.142	32.457	71.064
annotated area, %:	63.114	63.114	63.114	63.114	63.114
true label:	Endocapillary	Endocapillary	Endocapillary	Endocapillary	Endocapillary
predicted label:	Endocapillary	Endocapillary	Endocapillary	Endocapillary	Endocapillary
id: 052	CV fold 1	CV fold 2	CV fold 3	CV fold 4	CV fold 5

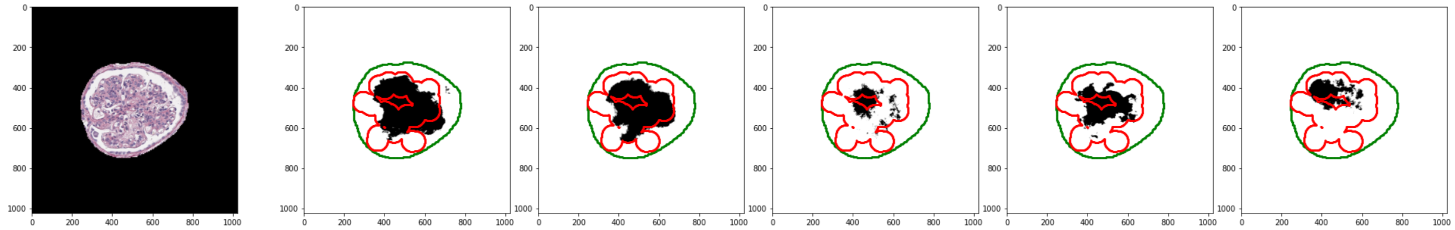

predicted area, %:	38.204	34.522	9.130	19.571	11.868
annotated area, %:	55.992	55.992	55.992	55.992	55.992
true label:	Endocapillary	Endocapillary	Endocapillary	Endocapillary	Endocapillary
predicted label:	Membranoproliferative	Membranoproliferative	Membranoproliferative	Membranoproliferative	Endocapillary
id: 053	CV fold 1	CV fold 2	CV fold 3	CV fold 4	CV fold 5

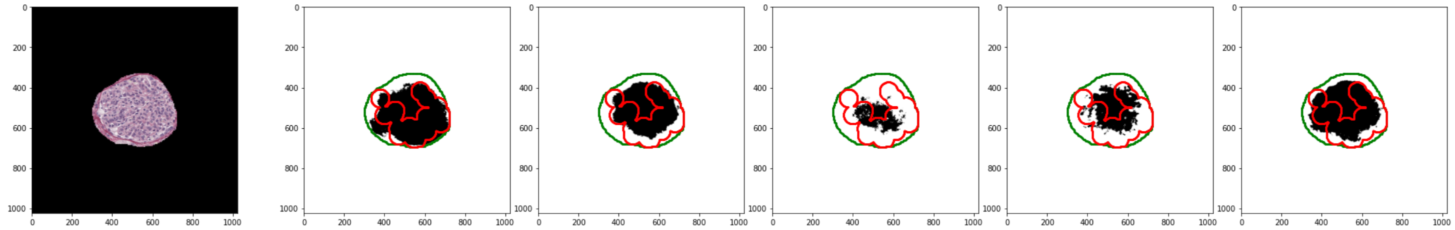

predicted area, %:	72.053	55.532	18.751	36.184	63.373
annotated area, %:	59.522	59.522	59.522	59.522	59.522
true label:	Endocapillary	Endocapillary	Endocapillary	Endocapillary	Endocapillary
predicted label:	Crescentic	Endocapillary	Endocapillary	Crescentic	Endocapillary
id: 054	CV fold 1	CV fold 2	CV fold 3	CV fold 4	CV fold 5

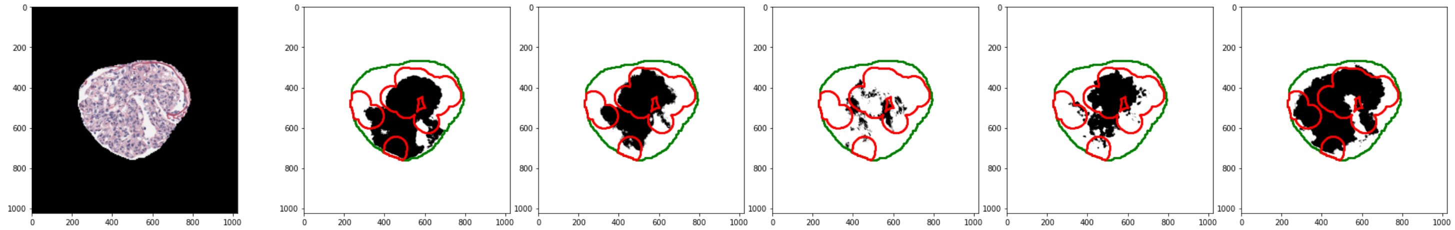

predicted area, %:	48.694	38.583	8.744	28.029	59.270
annotated area, %:	52.972	52.972	52.972	52.972	52.972
true label:	Endocapillary	Endocapillary	Endocapillary	Endocapillary	Endocapillary
predicted label:	Endocapillary	Endocapillary	Endocapillary	Endocapillary	Endocapillary
id: 055	CV fold 1	CV fold 2	CV fold 3	CV fold 4	CV fold 5

Supplementary Figure S2 (continued). Comparison of pattern quantification results per glomerular basis.

Red contour: annotated area @ 0.5 threshold **Green contour:** total area **Black fill:** predicted area @ 0.5 threshold

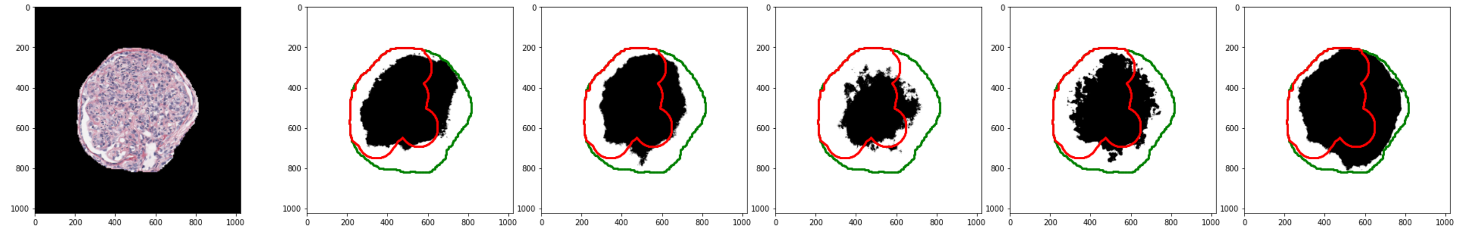

predicted area, %:	57.912	55.399	35.082	50.401	75.548
annotated area, %:	61.849	61.849	61.849	61.849	61.849
true label:	Endocapillary	Endocapillary	Endocapillary	Endocapillary	Endocapillary
predicted label:	Endocapillary	Endocapillary	Endocapillary	Endocapillary	Endocapillary
id: 056	CV fold 1	CV fold 2	CV fold 3	CV fold 4	CV fold 5

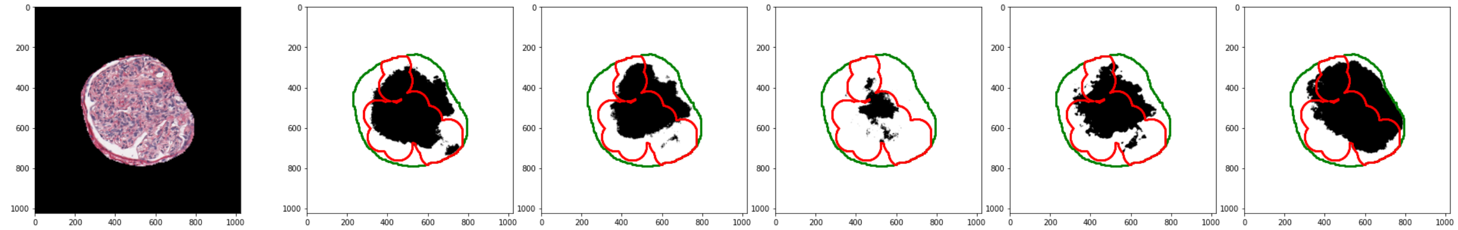

predicted area, %:	50.383	40.835	9.799	37.371	60.070
annotated area, %:	63.101	63.101	63.101	63.101	63.101
true label:	Endocapillary	Endocapillary	Endocapillary	Endocapillary	Endocapillary
predicted label:	Endocapillary	Endocapillary	Endocapillary	Endocapillary	Endocapillary
id: 057	CV fold 1	CV fold 2	CV fold 3	CV fold 4	CV fold 5

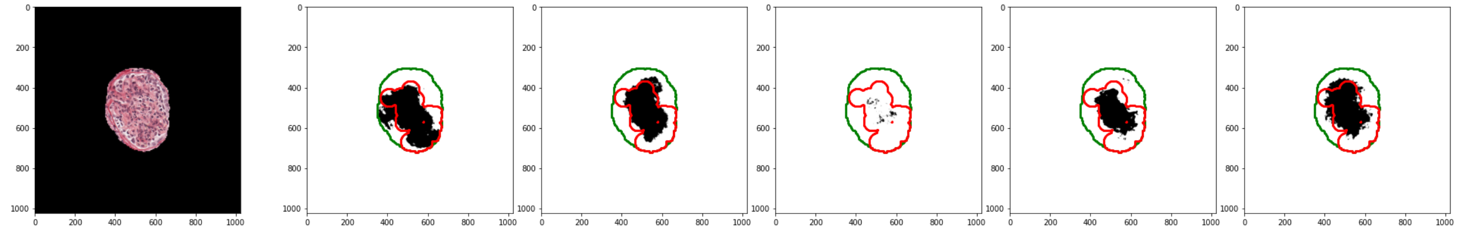

predicted area, %:	50.408	41.348	1.458	26.855	40.324
annotated area, %:	58.198	58.198	58.198	58.198	58.198
true label:	Endocapillary	Endocapillary	Endocapillary	Endocapillary	Endocapillary
predicted label:	Mesangioproliferative	Mesangioproliferative	Endocapillary	Endocapillary	Endocapillary
id: 058	CV fold 1	CV fold 2	CV fold 3	CV fold 4	CV fold 5

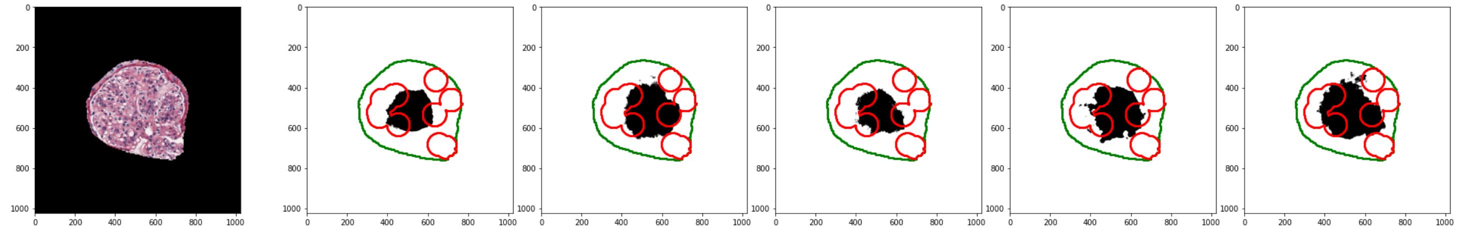

predicted area, %:	19.409	29.464	19.403	27.415	34.525
annotated area, %:	41.693	41.693	41.693	41.693	41.693
true label:	Endocapillary	Endocapillary	Endocapillary	Endocapillary	Endocapillary
predicted label:	Endocapillary	Membranoproliferative	Membranoproliferative	Membranoproliferative	Endocapillary
id: 059	CV fold 1	CV fold 2	CV fold 3	CV fold 4	CV fold 5

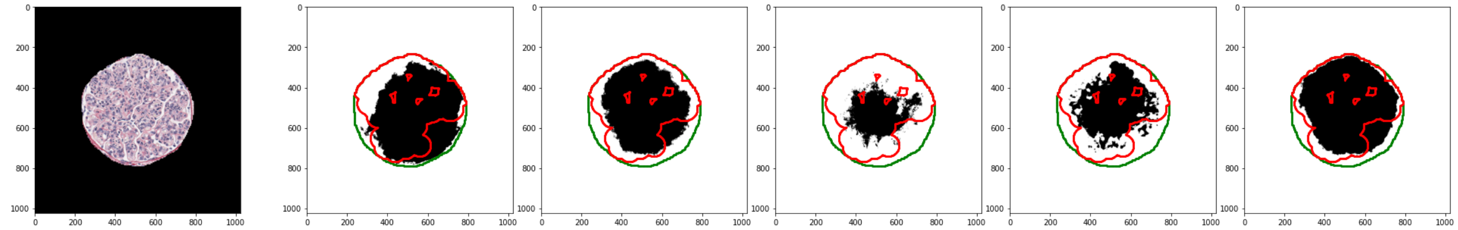

predicted area, %:	72.331	63.686	26.306	44.205	75.041
annotated area, %:	78.882	78.882	78.882	78.882	78.882
true label:	Endocapillary	Endocapillary	Endocapillary	Endocapillary	Endocapillary
predicted label:	Endocapillary	Endocapillary	Endocapillary	Endocapillary	Endocapillary
id: 060	CV fold 1	CV fold 2	CV fold 3	CV fold 4	CV fold 5

Supplementary Figure S2 (continued). Comparison of pattern quantification results per glomerular basis.

Red contour: annotated area @ 0.5 threshold **Green contour:** total area **Black fill:** predicted area @ 0.5 threshold

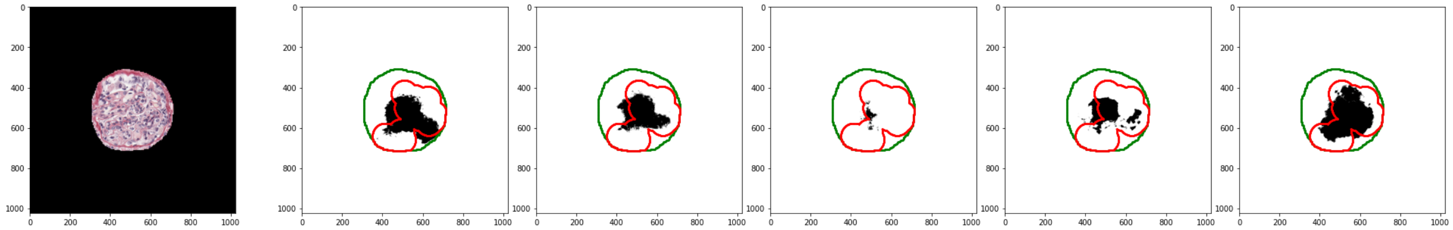

predicted area, %:	29.890	22.105	2.053	15.298	36.195
annotated area, %:	58.613	58.613	58.613	58.613	58.613
true label:	Endocapillary	Endocapillary	Endocapillary	Endocapillary	Endocapillary
predicted label:	FSGS	Mesangioproliferative	FSGS	FSGS	Endocapillary
id: 061	CV fold 1	CV fold 2	CV fold 3	CV fold 4	CV fold 5

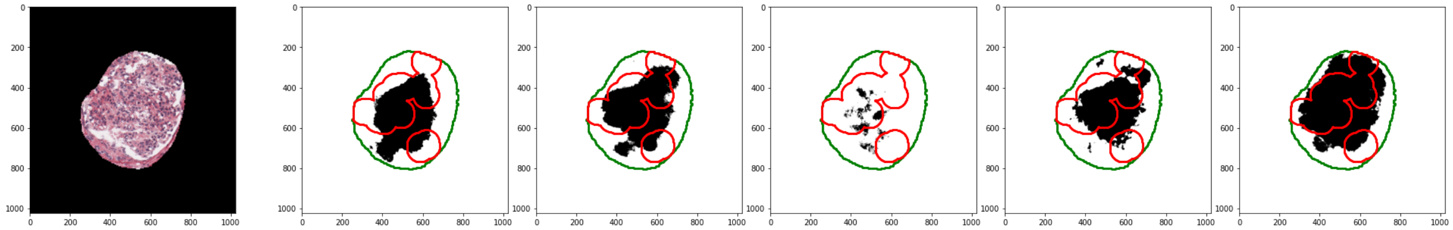

predicted area, %:	40.116	44.960	5.177	38.393	64.289
annotated area, %:	50.374	50.374	50.374	50.374	50.374
true label:	Endocapillary	Endocapillary	Endocapillary	Endocapillary	Endocapillary
predicted label:	Endocapillary	Endocapillary	Endocapillary	Endocapillary	Endocapillary
id: 062	CV fold 1	CV fold 2	CV fold 3	CV fold 4	CV fold 5

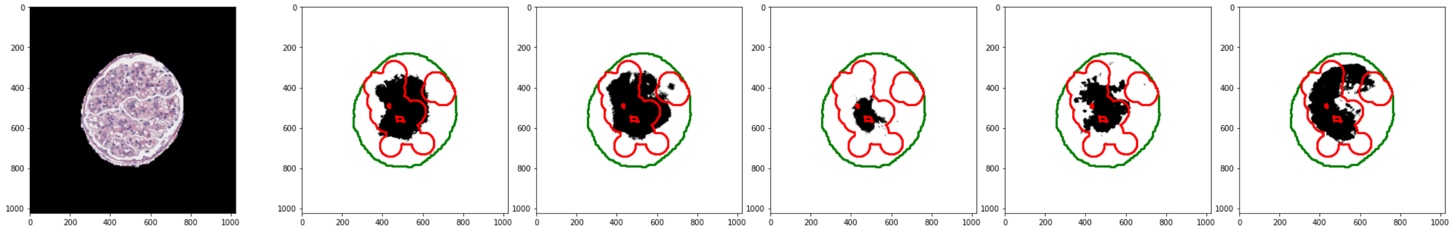

predicted area, %:	29.425	34.592	7.495	19.764	32.079
annotated area, %:	51.694	51.694	51.694	51.694	51.694
true label:	Endocapillary	Endocapillary	Endocapillary	Endocapillary	Endocapillary
predicted label:	Membranoproliferative	Membranoproliferative	Membranoproliferative	Membranoproliferative	Endocapillary
id: 063	CV fold 1	CV fold 2	CV fold 3	CV fold 4	CV fold 5

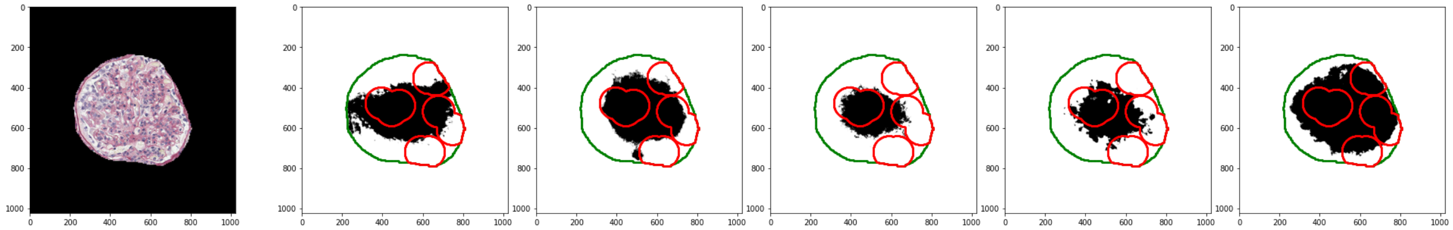

predicted area, %:	46.264	43.607	23.553	29.143	66.650
annotated area, %:	45.824	45.824	45.824	45.824	45.824
true label:	Endocapillary	Endocapillary	Endocapillary	Endocapillary	Endocapillary
predicted label:	Endocapillary	Endocapillary	Endocapillary	Crescentic	Endocapillary
id: 064	CV fold 1	CV fold 2	CV fold 3	CV fold 4	CV fold 5

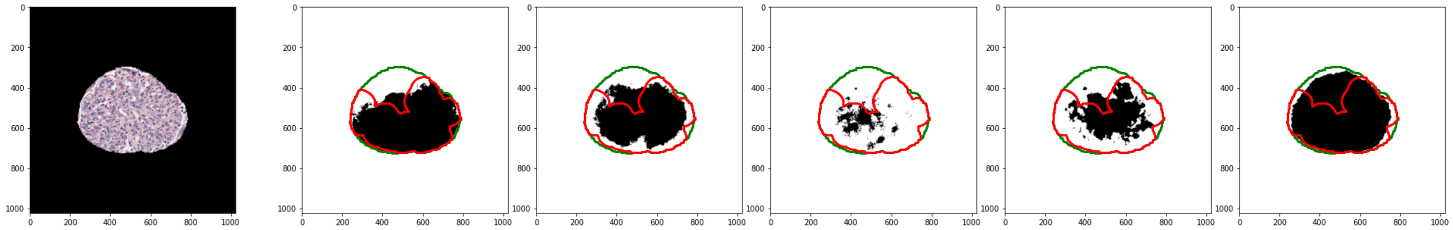

predicted area, %:	73.258	61.052	11.497	37.195	85.885
annotated area, %:	75.289	75.289	75.289	75.289	75.289
true label:	Endocapillary	Endocapillary	Endocapillary	Endocapillary	Endocapillary
predicted label:	Endocapillary	Endocapillary	Crescentic	Endocapillary	Endocapillary
id: 065	CV fold 1	CV fold 2	CV fold 3	CV fold 4	CV fold 5

Supplementary Figure S2 (continued). Comparison of pattern quantification results per glomerular basis.

Red contour: annotated area @ 0.5 threshold **Green contour:** total area **Black fill:** predicted area @ 0.5 threshold

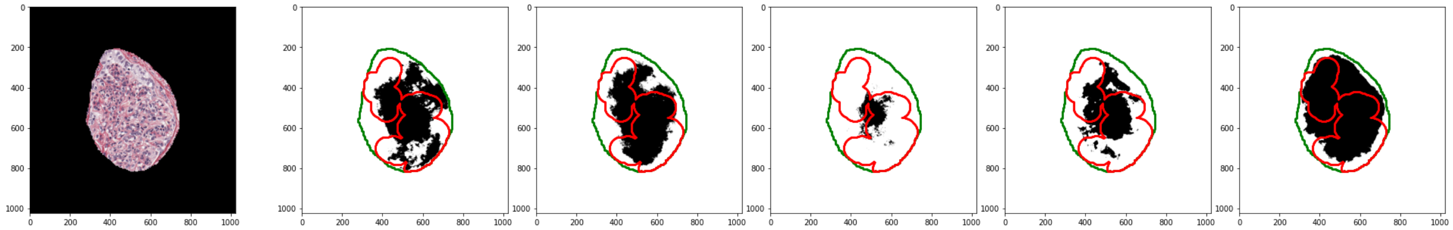

predicted area, %:	48.791	52.333	9.314	32.457	74.057
annotated area, %:	63.268	63.268	63.268	63.268	63.268
true label:	Endocapillary	Endocapillary	Endocapillary	Endocapillary	Endocapillary
predicted label:	Endocapillary	Endocapillary	Endocapillary	Endocapillary	Endocapillary
id: 066	CV fold 1	CV fold 2	CV fold 3	CV fold 4	CV fold 5

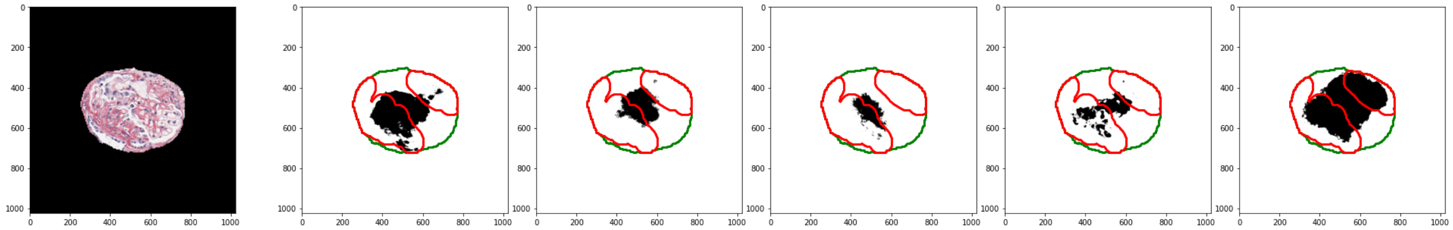

predicted area, %:	30.031	15.124	9.175	12.808	50.584
annotated area, %:	61.343	61.343	61.343	61.343	61.343
true label:	FSGS	FSGS	FSGS	FSGS	FSGS
predicted label:	FSGS	FSGS	FSGS	FSGS	FSGS
id: 067	CV fold 1	CV fold 2	CV fold 3	CV fold 4	CV fold 5

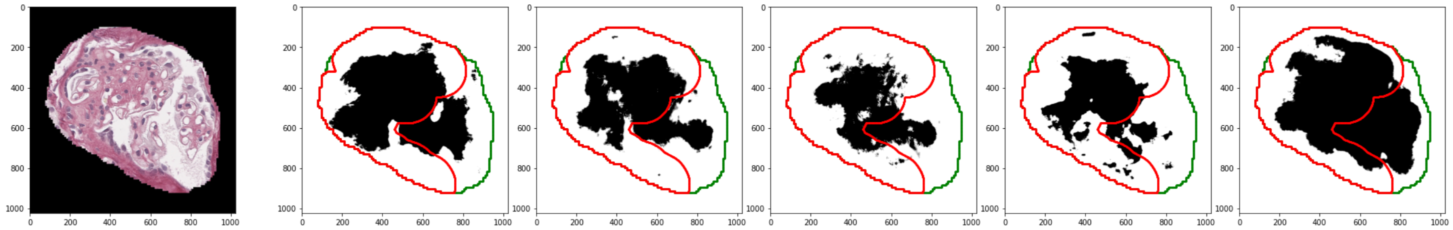

predicted area, %:	47.226	36.871	27.513	29.201	60.333
annotated area, %:	70.985	70.985	70.985	70.985	70.985
true label:	FSGS	FSGS	FSGS	FSGS	FSGS
predicted label:	Hypertrophy	Hypertrophy	Hypertrophy	Hypertrophy	Hypertrophy
id: 068	CV fold 1	CV fold 2	CV fold 3	CV fold 4	CV fold 5

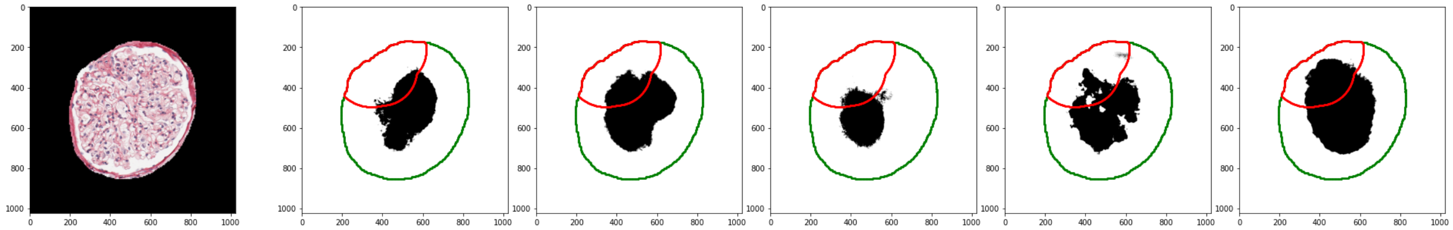

predicted area, %:	22.246	29.197	14.731	26.024	37.497
annotated area, %:	25.841	25.841	25.841	25.841	25.841
true label:	FSGS	FSGS	FSGS	FSGS	FSGS
predicted label:	Hypertrophy	Hypertrophy	Hypertrophy	Hypertrophy	Hypertrophy
id: 069	CV fold 1	CV fold 2	CV fold 3	CV fold 4	CV fold 5

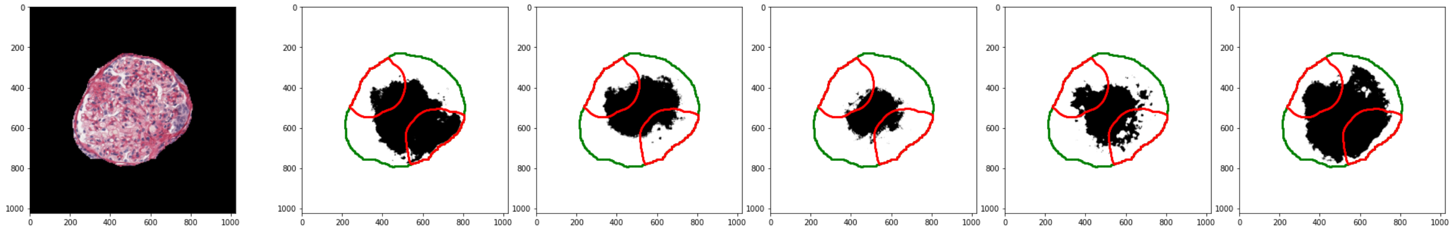

predicted area, %:	46.594	33.831	19.082	30.270	56.257
annotated area, %:	40.862	40.862	40.862	40.862	40.862
true label:	FSGS	FSGS	FSGS	FSGS	FSGS
predicted label:	Endocapillary	Endocapillary	Endocapillary	Endocapillary	Endocapillary
id: 070	CV fold 1	CV fold 2	CV fold 3	CV fold 4	CV fold 5

Supplementary Figure S2 (continued). Comparison of pattern quantification results per glomerular basis.

Red contour: annotated area @ 0.5 threshold **Green contour:** total area **Black fill:** predicted area @ 0.5 threshold

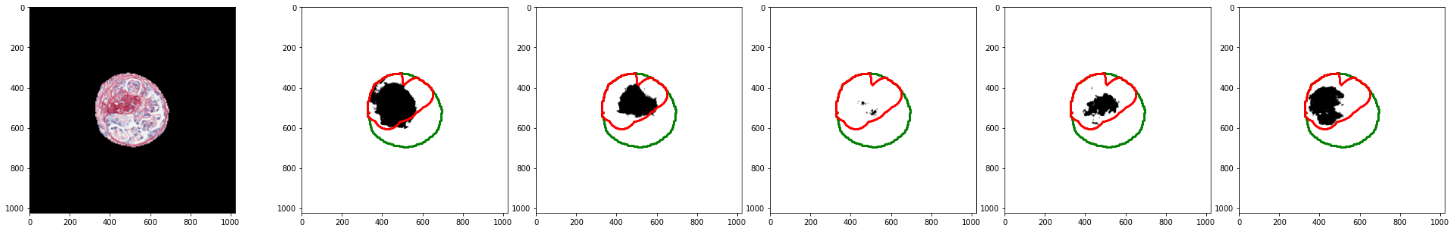

predicted area, %:	36.013	20.738	0.835	11.920	22.326
annotated area, %:	57.377	57.377	57.377	57.377	57.377
true label:	FSGS	FSGS	FSGS	FSGS	FSGS
predicted label:	FSGS	FSGS	FSGS	FSGS	FSGS
id: 071	CV fold 1	CV fold 2	CV fold 3	CV fold 4	CV fold 5

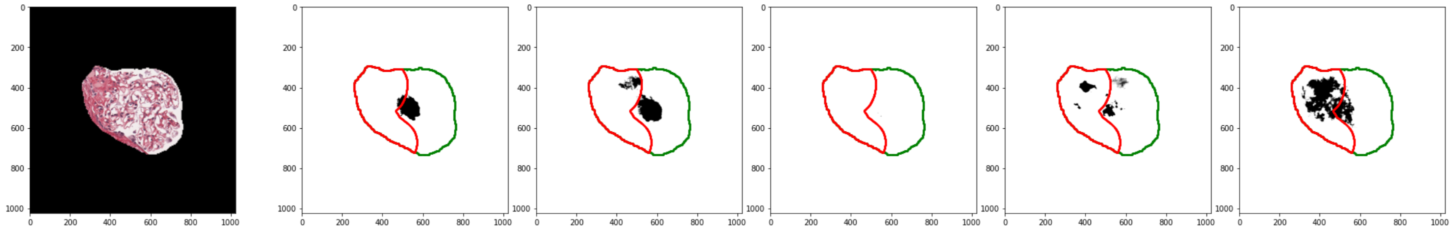

predicted area, %:	5.706	8.693	0.006	4.111	18.339
annotated area, %:	47.528	47.528	47.528	47.528	47.528
true label:	FSGS	FSGS	FSGS	FSGS	FSGS
predicted label:	FSGS	FSGS	Membranous	FSGS	FSGS
id: 072	CV fold 1	CV fold 2	CV fold 3	CV fold 4	CV fold 5

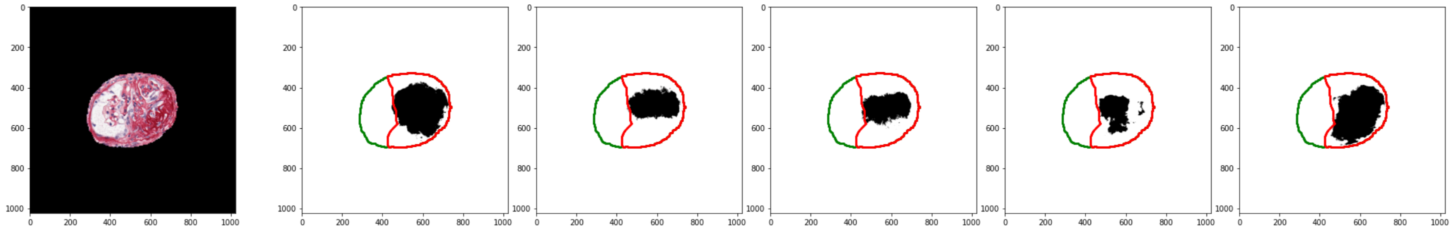

predicted area, %:	39.669	23.087	21.750	16.201	38.651
annotated area, %:	66.776	66.776	66.776	66.776	66.776
true label:	FSGS	FSGS	FSGS	FSGS	FSGS
predicted label:	FSGS	FSGS	FSGS	FSGS	FSGS
id: 073	CV fold 1	CV fold 2	CV fold 3	CV fold 4	CV fold 5

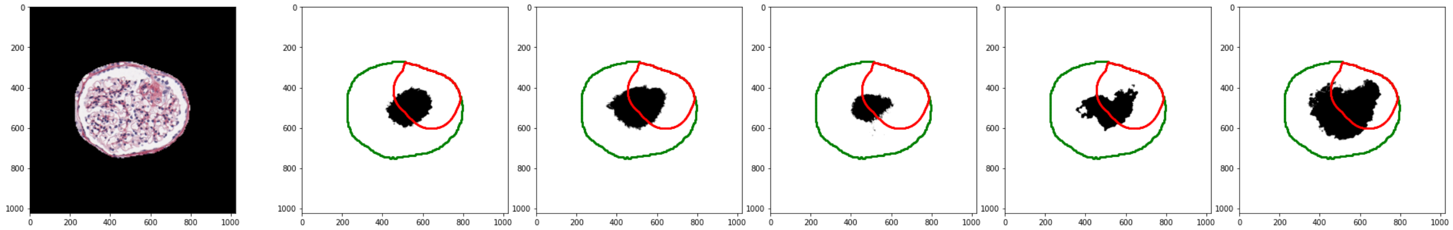

predicted area, %:	13.953	19.272	9.248	15.498	32.396
annotated area, %:	35.157	35.157	35.157	35.157	35.157
true label:	FSGS	FSGS	FSGS	FSGS	FSGS
predicted label:	Normal	Normal	Normal	Normal	Normal
id: 074	CV fold 1	CV fold 2	CV fold 3	CV fold 4	CV fold 5

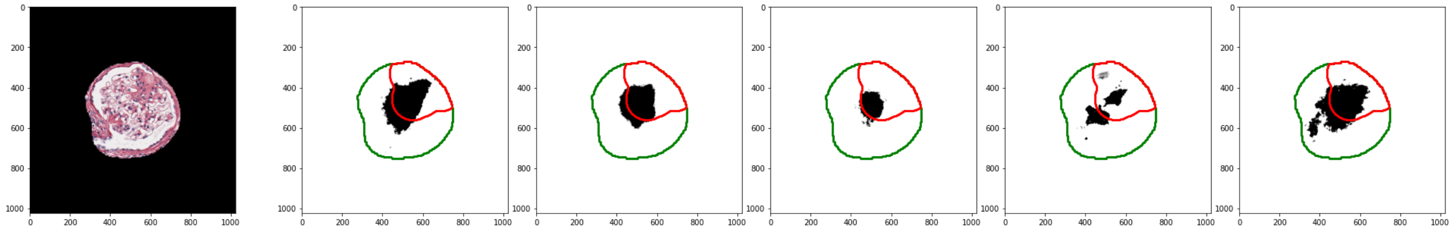

predicted area, %:	22.409	16.817	7.976	8.576	25.107
annotated area, %:	35.667	35.667	35.667	35.667	35.667
true label:	FSGS	FSGS	FSGS	FSGS	FSGS
predicted label:	FSGS	Membranous	FSGS	FSGS	FSGS
id: 075	CV fold 1	CV fold 2	CV fold 3	CV fold 4	CV fold 5

Supplementary Figure S2 (continued). Comparison of pattern quantification results per glomerular basis.

Red contour: annotated area @ 0.5 threshold **Green contour:** total area **Black fill:** predicted area @ 0.5 threshold

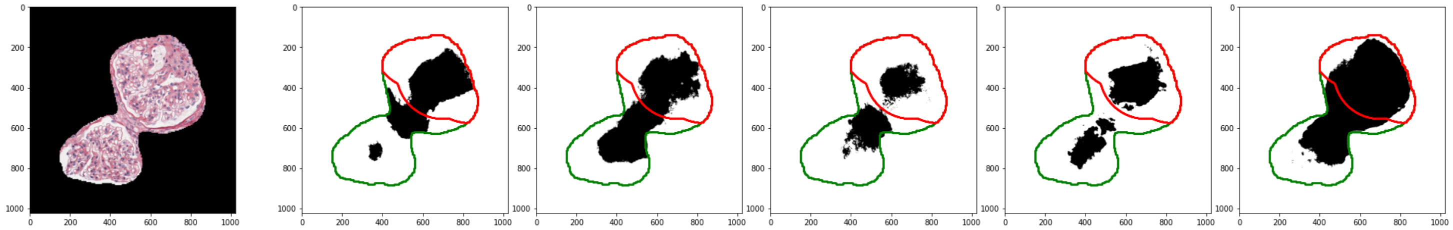

predicted area, %:	33.278	41.837	21.506	25.192	61.609
annotated area, %:	49.364	49.364	49.364	49.364	49.364
true label:	FSGS	FSGS	FSGS	FSGS	FSGS
predicted label:	Membranoproliferative	Membranoproliferative	Membranous	Membranous	Membranous
id: 076	CV fold 1	CV fold 2	CV fold 3	CV fold 4	CV fold 5

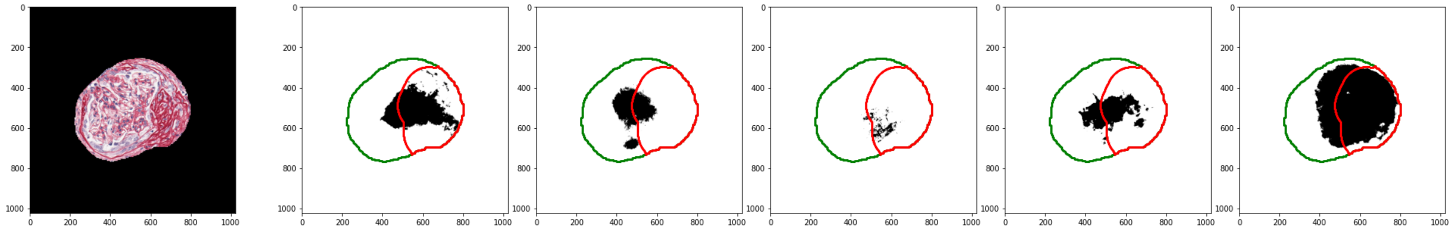

predicted area, %:	22.844	13.896	2.505	15.041	58.144
annotated area, %:	47.058	47.058	47.058	47.058	47.058
true label:	FSGS	FSGS	FSGS	FSGS	FSGS
predicted label:	FSGS	FSGS	FSGS	FSGS	FSGS
id: 077	CV fold 1	CV fold 2	CV fold 3	CV fold 4	CV fold 5

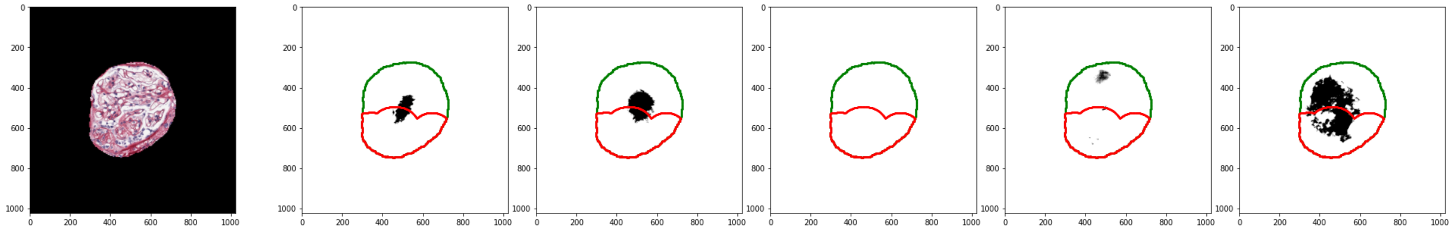

predicted area, %:	4.691	8.669	0.000	1.241	26.980
annotated area, %:	46.099	46.099	46.099	46.099	46.099
true label:	FSGS	FSGS	FSGS	FSGS	FSGS
predicted label:	FSGS	FSGS	FSGS	FSGS	FSGS
id: 078	CV fold 1	CV fold 2	CV fold 3	CV fold 4	CV fold 5

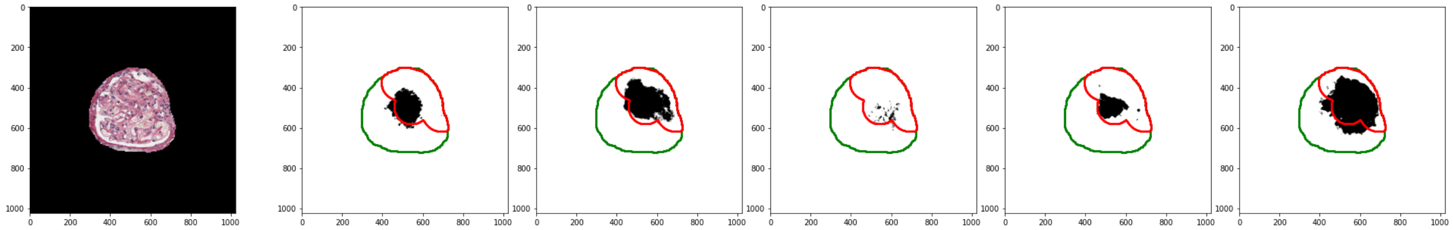

predicted area, %:	15.175	23.987	1.753	8.996	38.632
annotated area, %:	47.358	47.358	47.358	47.358	47.358
true label:	FSGS	FSGS	FSGS	FSGS	FSGS
predicted label:	Mesangioproliferative	Mesangioproliferative	Mesangioproliferative	FSGS	Mesangioproliferative
id: 079	CV fold 1	CV fold 2	CV fold 3	CV fold 4	CV fold 5

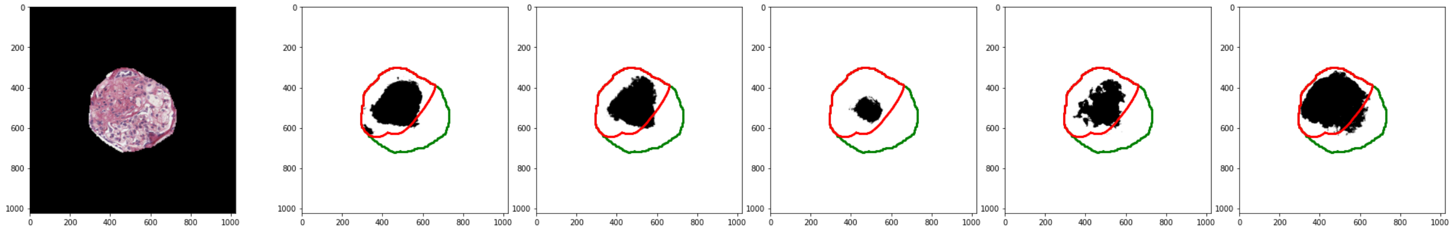

predicted area, %:	32.677	31.179	9.027	25.705	50.952
annotated area, %:	62.880	62.880	62.880	62.880	62.880
true label:	FSGS	FSGS	FSGS	FSGS	FSGS
predicted label:	FSGS	FSGS	FSGS	FSGS	Endocapillary
id: 080	CV fold 1	CV fold 2	CV fold 3	CV fold 4	CV fold 5

Supplementary Figure S2 (continued). Comparison of pattern quantification results per glomerular basis.

Red contour: annotated area @ 0.5 threshold **Green contour:** total area **Black fill:** predicted area @ 0.5 threshold

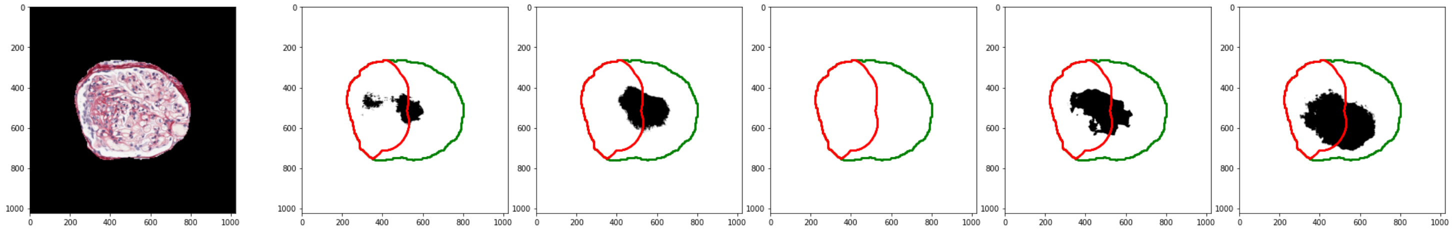

predicted area, %:	7.679	15.498	0.000	18.142	30.335
annotated area, %:	48.475	48.475	48.475	48.475	48.475
true label:	FSGS	FSGS	FSGS	FSGS	FSGS
predicted label:	Normal	Hypertrophy	Normal	Normal	Hypertrophy
id: 081	CV fold 1	CV fold 2	CV fold 3	CV fold 4	CV fold 5

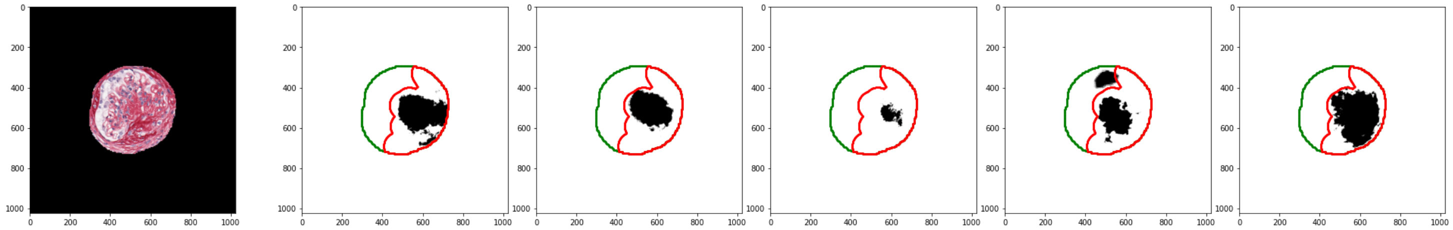

predicted area, %:	23.952	19.082	4.168	19.788	32.419
annotated area, %:	61.385	61.385	61.385	61.385	61.385
true label:	FSGS	FSGS	FSGS	FSGS	FSGS
predicted label:	FSGS	FSGS	FSGS	FSGS	FSGS
id: 082	CV fold 1	CV fold 2	CV fold 3	CV fold 4	CV fold 5

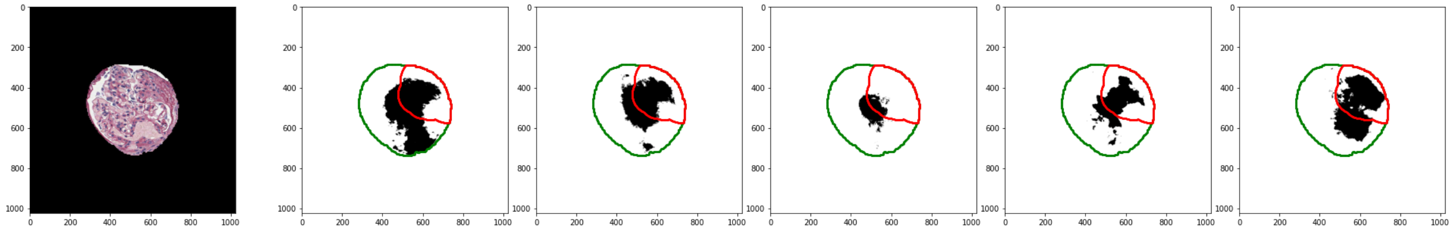

predicted area, %:	39.627	28.517	9.774	19.529	32.533
annotated area, %:	34.547	34.547	34.547	34.547	34.547
true label:	FSGS	FSGS	FSGS	FSGS	FSGS
predicted label:	Crescentic	Crescentic	Crescentic	Crescentic	Crescentic
id: 083	CV fold 1	CV fold 2	CV fold 3	CV fold 4	CV fold 5

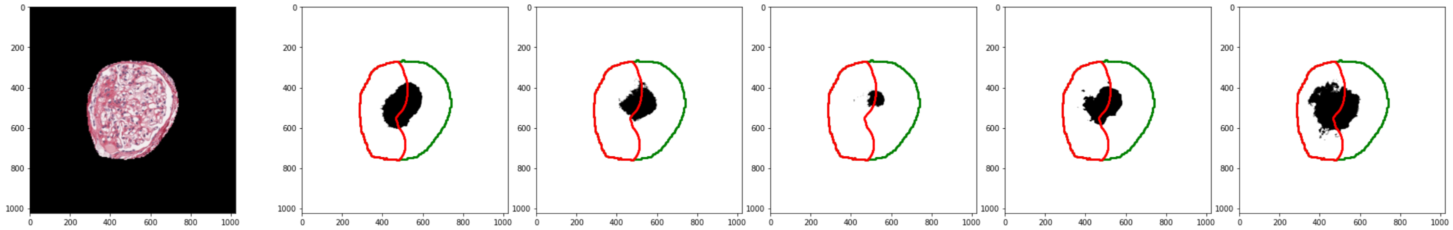

predicted area, %:	17.347	11.661	3.032	13.389	23.914
annotated area, %:	50.374	50.374	50.374	50.374	50.374
true label:	FSGS	FSGS	FSGS	FSGS	FSGS
predicted label:	Membranous	Membranous	Membranous	Membranous	Membranous
id: 084	CV fold 1	CV fold 2	CV fold 3	CV fold 4	CV fold 5

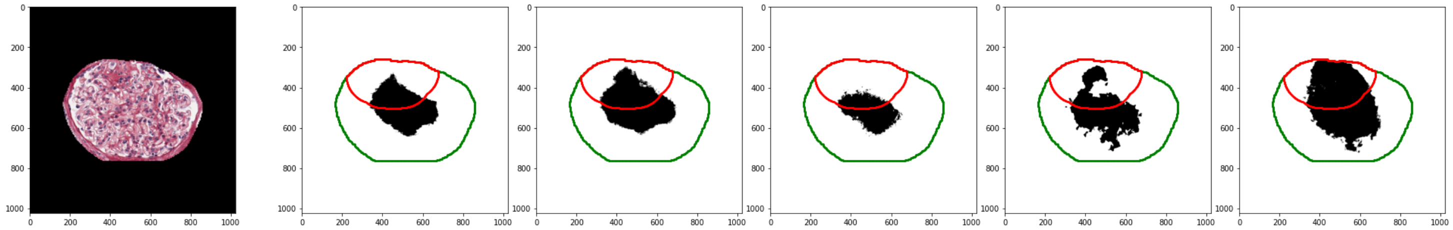

predicted area, %:	21.251	26.567	14.209	24.746	39.963
annotated area, %:	30.562	30.562	30.562	30.562	30.562
true label:	FSGS	FSGS	FSGS	FSGS	FSGS
predicted label:	Hypertrophy	Hypertrophy	Hypertrophy	Membranous	Hypertrophy
id: 085	CV fold 1	CV fold 2	CV fold 3	CV fold 4	CV fold 5

Supplementary Figure S2 (continued). Comparison of pattern quantification results per glomerular basis.

Red contour: annotated area @ 0.5 threshold **Green contour:** total area **Black fill:** predicted area @ 0.5 threshold

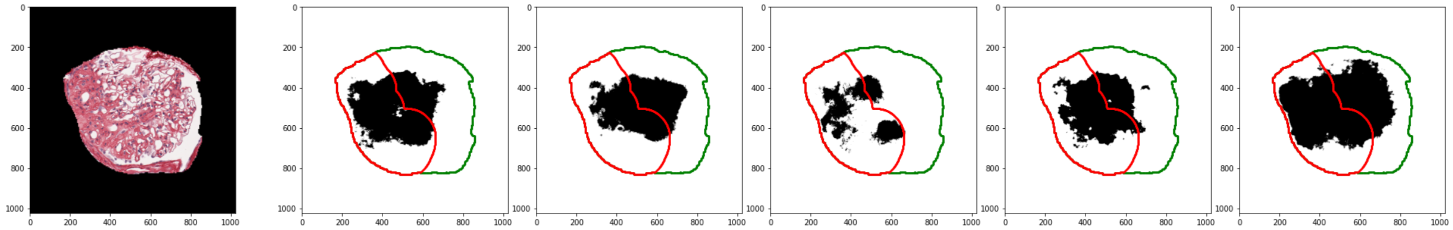

predicted area, %:	36.166	31.278	15.233	29.995	52.178
annotated area, %:	51.218	51.218	51.218	51.218	51.218
true label:	FSGS	FSGS	FSGS	FSGS	FSGS
predicted label:	FSGS	Hypertrophy	FSGS	Hypertrophy	Hypertrophy
id: 086	CV fold 1	CV fold 2	CV fold 3	CV fold 4	CV fold 5

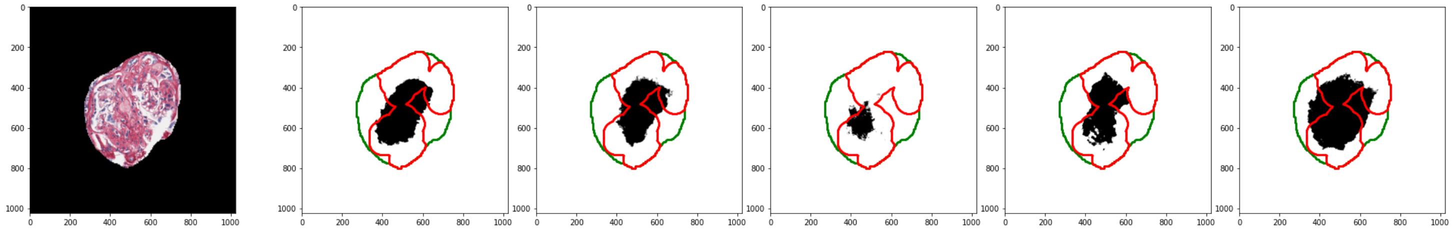

predicted area, %:	29.410	26.909	7.857	24.088	43.106
annotated area, %:	68.743	68.743	68.743	68.743	68.743
true label:	FSGS	FSGS	FSGS	FSGS	FSGS
predicted label:	FSGS	FSGS	FSGS	FSGS	FSGS
id: 087	CV fold 1	CV fold 2	CV fold 3	CV fold 4	CV fold 5

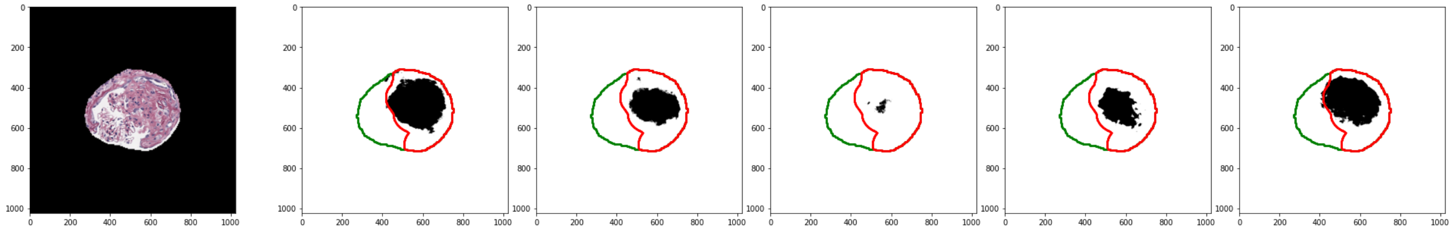

predicted area, %:	38.214	23.418	1.423	17.856	34.776
annotated area, %:	66.077	66.077	66.077	66.077	66.077
true label:	FSGS	FSGS	FSGS	FSGS	FSGS
predicted label:	Crescentic	FSGS	Crescentic	FSGS	Crescentic
id: 088	CV fold 1	CV fold 2	CV fold 3	CV fold 4	CV fold 5

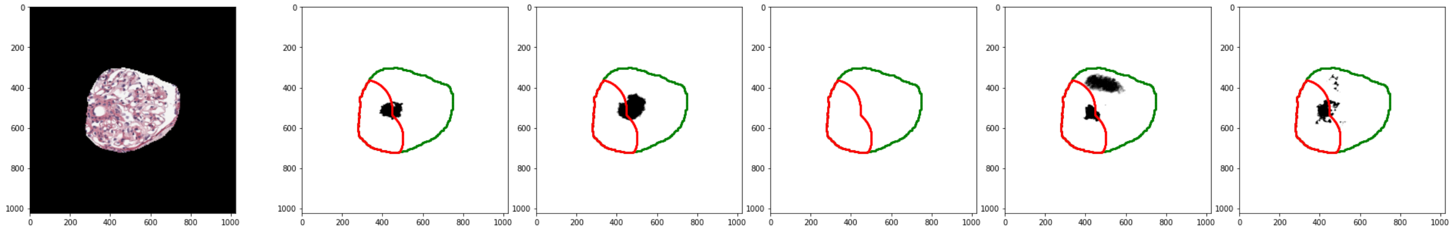

predicted area, %:	4.471	8.152	0.000	9.455	4.801
annotated area, %:	36.479	36.479	36.479	36.479	36.479
true label:	FSGS	FSGS	FSGS	FSGS	FSGS
predicted label:	Mesangioproliferative	Mesangioproliferative	Mesangioproliferative	Membranous	Mesangioproliferative
id: 089	CV fold 1	CV fold 2	CV fold 3	CV fold 4	CV fold 5

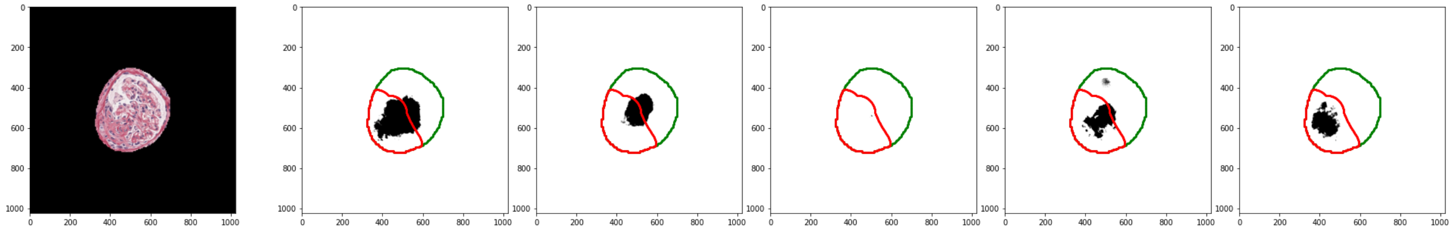

predicted area, %:	27.994	13.863	0.022	12.670	11.371
annotated area, %:	46.791	46.791	46.791	46.791	46.791
true label:	FSGS	FSGS	FSGS	FSGS	FSGS
predicted label:	FSGS	FSGS	FSGS	FSGS	FSGS
id: 090	CV fold 1	CV fold 2	CV fold 3	CV fold 4	CV fold 5

Supplementary Figure S2 (continued). Comparison of pattern quantification results per glomerular basis.

Red contour: annotated area @ 0.5 threshold **Green contour:** total area **Black fill:** predicted area @ 0.5 threshold

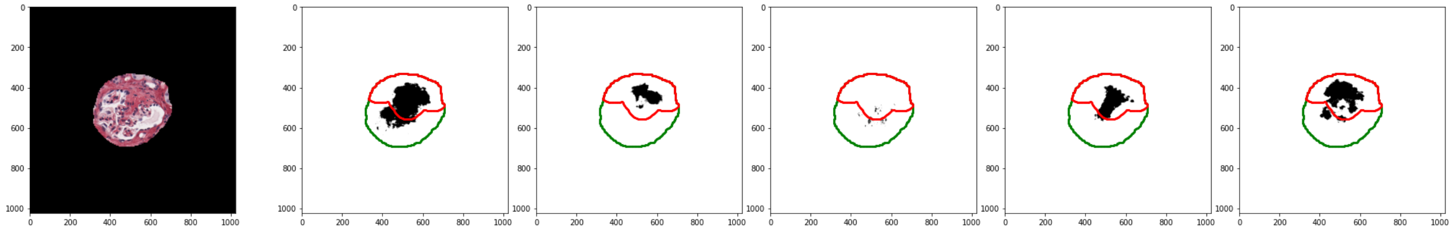

predicted area, %:	29.026	8.321	0.854	12.299	19.482
annotated area, %:	50.956	50.956	50.956	50.956	50.956
true label:	FSGS	FSGS	FSGS	FSGS	FSGS
predicted label:	FSGS	FSGS	FSGS	FSGS	FSGS
id: 091	CV fold 1	CV fold 2	CV fold 3	CV fold 4	CV fold 5

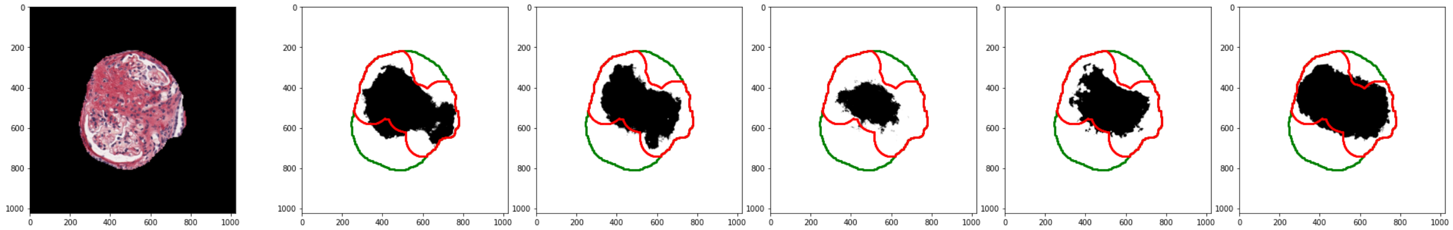

predicted area, %:	46.111	40.813	19.403	34.056	51.187
annotated area, %:	67.618	67.618	67.618	67.618	67.618
true label:	FSGS	FSGS	FSGS	FSGS	FSGS
predicted label:	Membranoproliferative	Membranoproliferative	Membranoproliferative	Membranoproliferative	Membranoproliferative
id: 092	CV fold 1	CV fold 2	CV fold 3	CV fold 4	CV fold 5

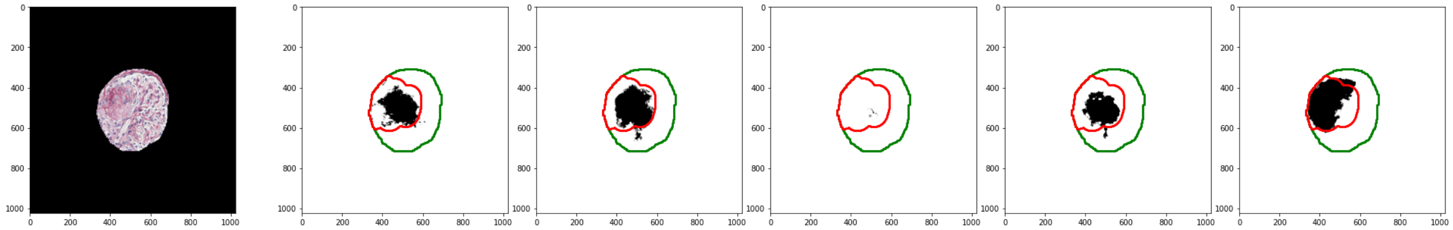

predicted area, %:	21.421	27.094	0.164	19.065	34.331
annotated area, %:	46.402	46.402	46.402	46.402	46.402
true label:	FSGS	FSGS	FSGS	FSGS	FSGS
predicted label:	Crescentic	Crescentic	Crescentic	Crescentic	Crescentic
id: 093	CV fold 1	CV fold 2	CV fold 3	CV fold 4	CV fold 5

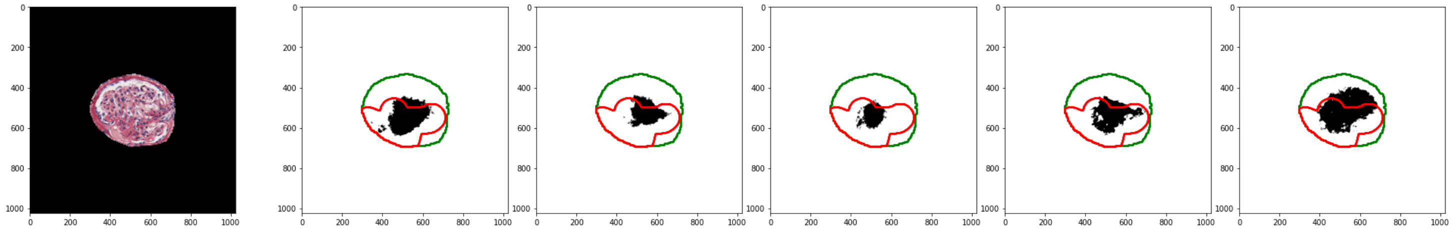

predicted area, %:	23.754	14.817	9.881	20.242	35.559
annotated area, %:	53.112	53.112	53.112	53.112	53.112
true label:	FSGS	FSGS	FSGS	FSGS	FSGS
predicted label:	FSGS	Mesangioproliferative	FSGS	FSGS	Mesangioproliferative
id: 094	CV fold 1	CV fold 2	CV fold 3	CV fold 4	CV fold 5

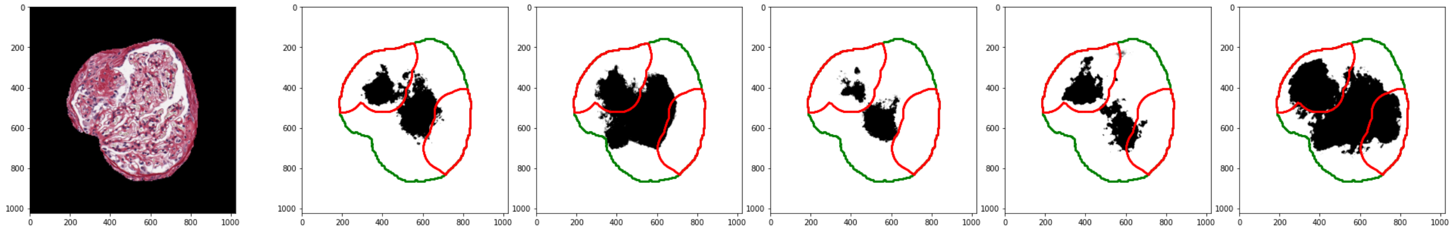

predicted area, %:	18.972	32.962	10.355	15.461	49.642
annotated area, %:	46.608	46.608	46.608	46.608	46.608
true label:	FSGS	FSGS	FSGS	FSGS	FSGS
predicted label:	FSGS	FSGS	FSGS	FSGS	FSGS
id: 095	CV fold 1	CV fold 2	CV fold 3	CV fold 4	CV fold 5

Supplementary Figure S2 (continued). Comparison of pattern quantification results per glomerular basis.

Red contour: annotated area @ 0.5 threshold **Green contour:** total area **Black fill:** predicted area @ 0.5 threshold

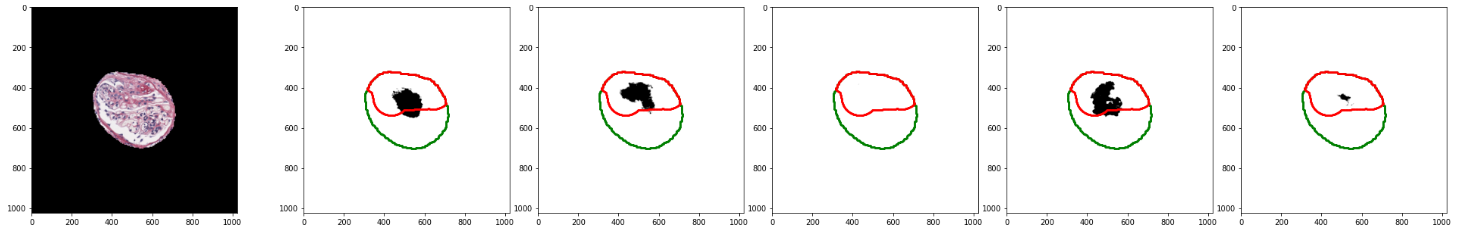

predicted area, %:	12.219	11.491	0.002	14.470	0.985
annotated area, %:	49.868	49.868	49.868	49.868	49.868
true label:	FSGS	FSGS	FSGS	FSGS	FSGS
predicted label:	FSGS	Mesangioproliferative	FSGS	FSGS	FSGS
id: 096	CV fold 1	CV fold 2	CV fold 3	CV fold 4	CV fold 5

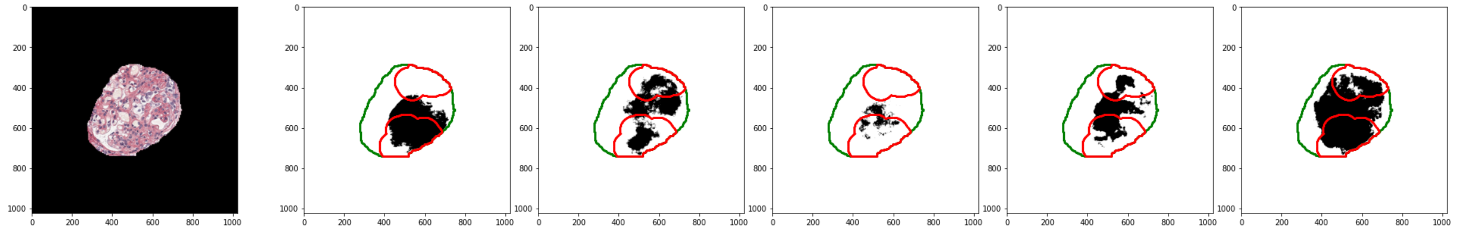

predicted area, %:	33.995	29.716	6.233	25.342	59.307
annotated area, %:	49.969	49.969	49.969	49.969	49.969
true label:	FSGS	FSGS	FSGS	FSGS	FSGS
predicted label:	FSGS	FSGS	FSGS	FSGS	FSGS
id: 097	CV fold 1	CV fold 2	CV fold 3	CV fold 4	CV fold 5

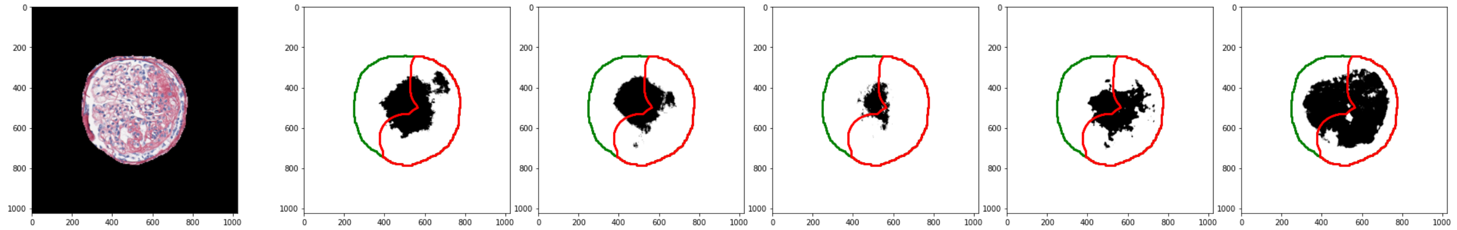

predicted area, %:	25.741	21.383	7.868	20.861	49.018
annotated area, %:	59.197	59.197	59.197	59.197	59.197
true label:	FSGS	FSGS	FSGS	FSGS	FSGS
predicted label:	FSGS	FSGS	FSGS	FSGS	FSGS
id: 098	CV fold 1	CV fold 2	CV fold 3	CV fold 4	CV fold 5

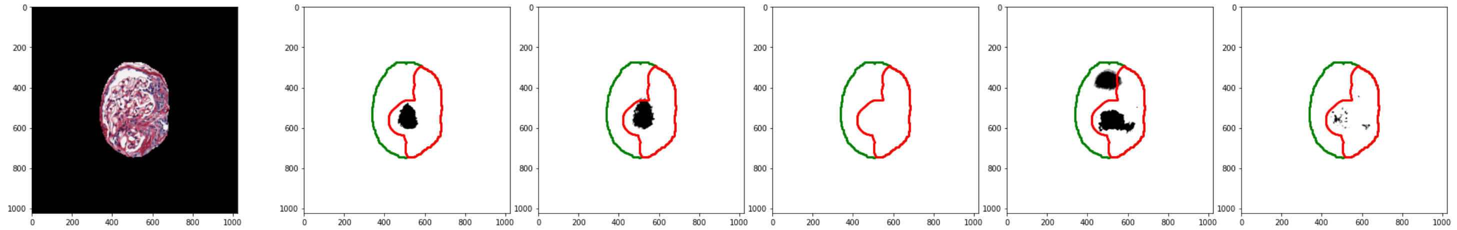

predicted area, %:	6.652	8.465	0.000	16.581	1.213
annotated area, %:	54.986	54.986	54.986	54.986	54.986
true label:	FSGS	FSGS	FSGS	FSGS	FSGS
predicted label:	Mesangioproliferative	Mesangioproliferative	Mesangioproliferative	FSGS	Mesangioproliferative
id: 099	CV fold 1	CV fold 2	CV fold 3	CV fold 4	CV fold 5

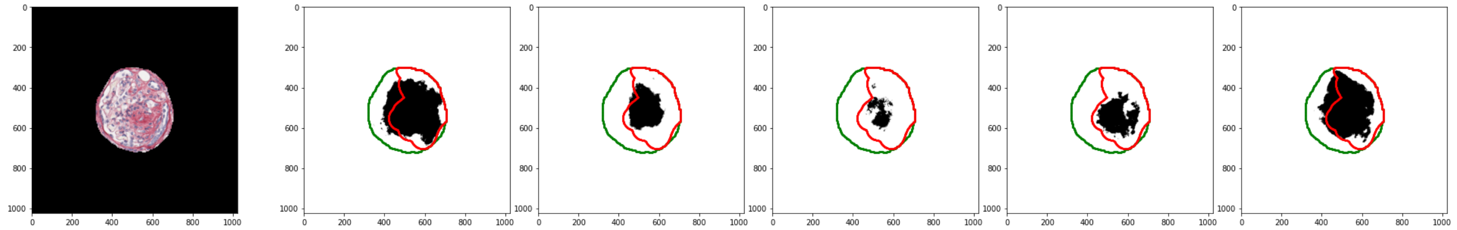

predicted area, %:	54.814	22.912	8.193	21.890	50.930
annotated area, %:	61.754	61.754	61.754	61.754	61.754
true label:	FSGS	FSGS	FSGS	FSGS	FSGS
predicted label:	FSGS	FSGS	FSGS	FSGS	FSGS
id: 100	CV fold 1	CV fold 2	CV fold 3	CV fold 4	CV fold 5

Supplementary Figure S2 (continued). Comparison of pattern quantification results per glomerular basis.

Red contour: annotated area @ 0.5 threshold **Green contour:** total area **Black fill:** predicted area @ 0.5 threshold

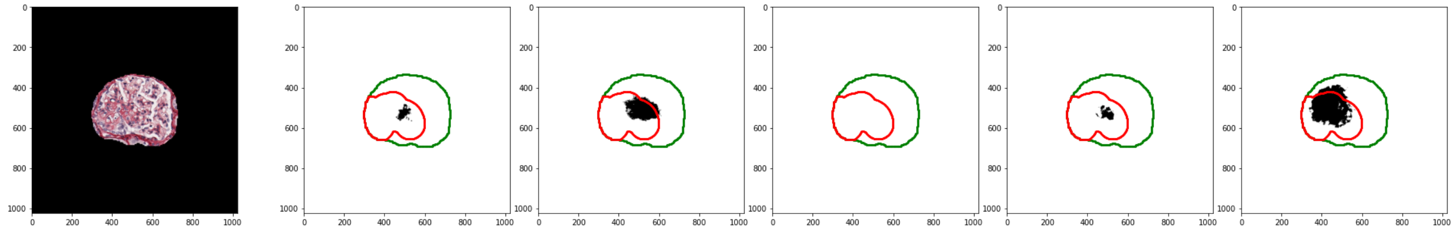

predicted area, %:	2.288	11.970	0.000	2.463	24.243
annotated area, %:	44.881	44.881	44.881	44.881	44.881
true label:	FSGS	FSGS	FSGS	FSGS	FSGS
predicted label:	Mesangioproliferative	Mesangioproliferative	Mesangioproliferative	FSGS	Mesangioproliferative
id: 101	CV fold 1	CV fold 2	CV fold 3	CV fold 4	CV fold 5

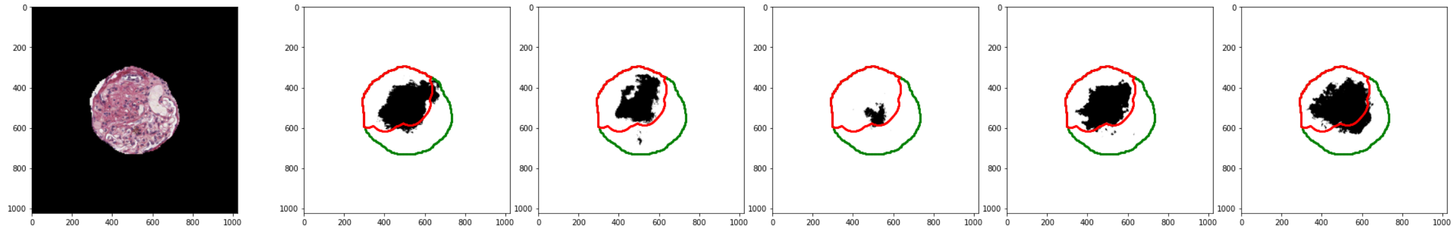

predicted area, %:	33.193	23.008	4.751	27.960	38.202
annotated area, %:	57.330	57.330	57.330	57.330	57.330
true label:	FSGS	FSGS	FSGS	FSGS	FSGS
predicted label:	FSGS	Mesangioproliferative	Endocapillary	FSGS	Endocapillary
id: 102	CV fold 1	CV fold 2	CV fold 3	CV fold 4	CV fold 5

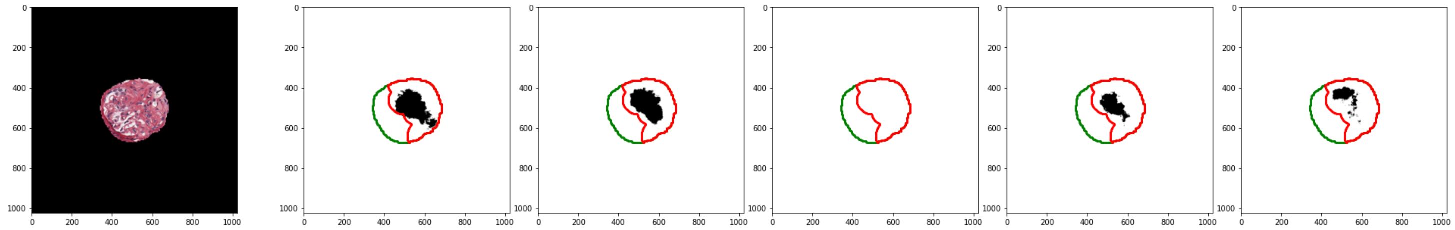

predicted area, %:	24.414	22.021	0.000	12.154	8.416
annotated area, %:	66.112	66.112	66.112	66.112	66.112
true label:	FSGS	FSGS	FSGS	FSGS	FSGS
predicted label:	FSGS	FSGS	FSGS	FSGS	FSGS
id: 103	CV fold 1	CV fold 2	CV fold 3	CV fold 4	CV fold 5

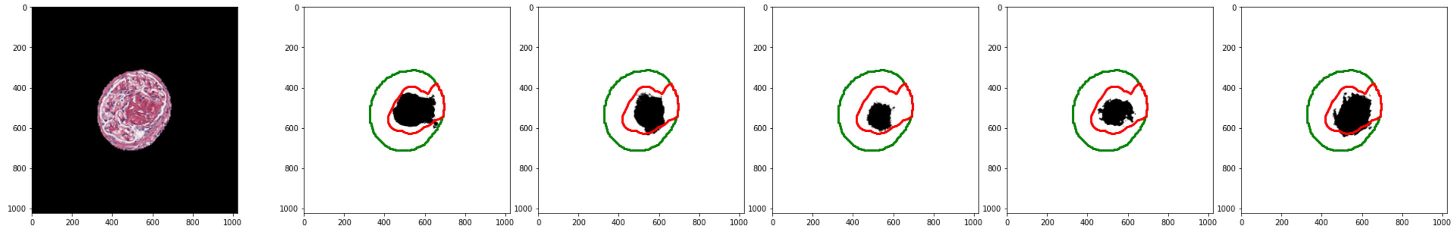

predicted area, %:	24.905	20.314	11.730	14.887	25.696
annotated area, %:	41.757	41.757	41.757	41.757	41.757
true label:	FSGS	FSGS	FSGS	FSGS	FSGS
predicted label:	FSGS	FSGS	FSGS	FSGS	FSGS
id: 104	CV fold 1	CV fold 2	CV fold 3	CV fold 4	CV fold 5

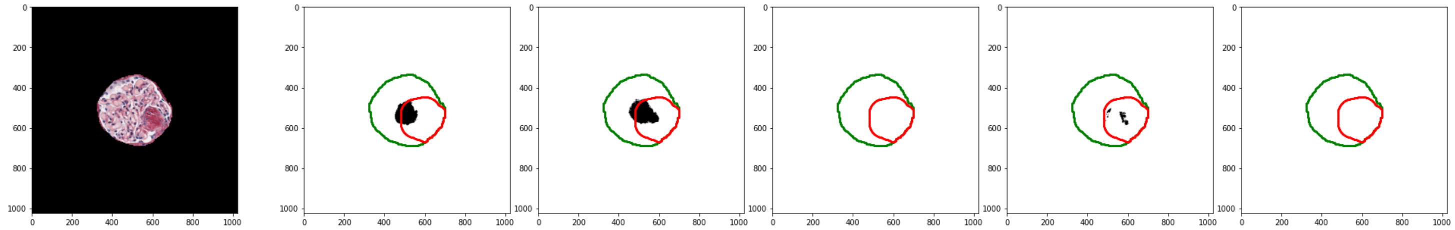

predicted area, %:	10.026	12.515	0.000	1.776	0.000
annotated area, %:	37.359	37.359	37.359	37.359	37.359
true label:	FSGS	FSGS	FSGS	FSGS	FSGS
predicted label:	Mesangioproliferative	Mesangioproliferative	Mesangioproliferative	Normal	Mesangioproliferative
id: 105	CV fold 1	CV fold 2	CV fold 3	CV fold 4	CV fold 5

Supplementary Figure S2 (continued). Comparison of pattern quantification results per glomerular basis.

Red contour: annotated area @ 0.5 threshold **Green contour:** total area **Black fill:** predicted area @ 0.5 threshold

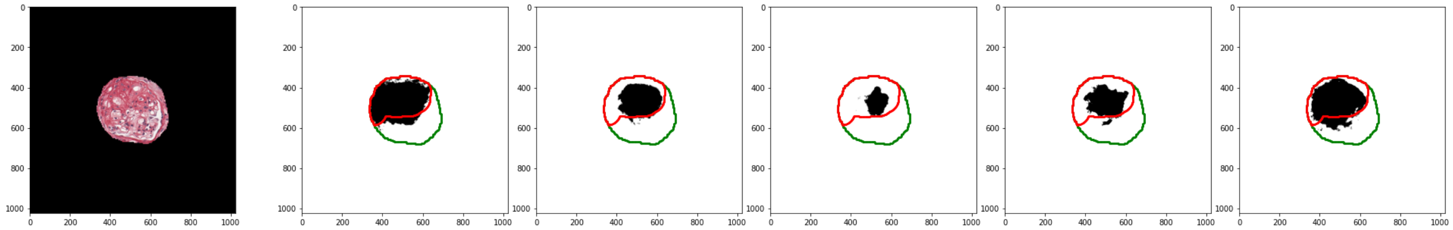

predicted area, %:	55.657	32.008	11.729	26.309	51.034
annotated area, %:	55.578	55.578	55.578	55.578	55.578
true label:	FSGS	FSGS	FSGS	FSGS	FSGS
predicted label:	Sclerosed	FSGS	FSGS	FSGS	FSGS
id: 106	CV fold 1	CV fold 2	CV fold 3	CV fold 4	CV fold 5

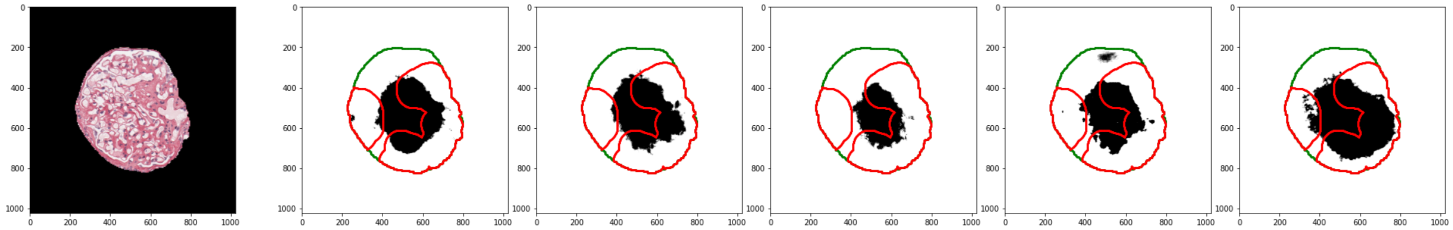

predicted area, %:	33.465	33.694	20.552	28.593	46.627
annotated area, %:	65.486	65.486	65.486	65.486	65.486
true label:	FSGS	FSGS	FSGS	FSGS	FSGS
predicted label:	Membranous	Membranous	Membranous	Membranous	Membranous
id: 107	CV fold 1	CV fold 2	CV fold 3	CV fold 4	CV fold 5

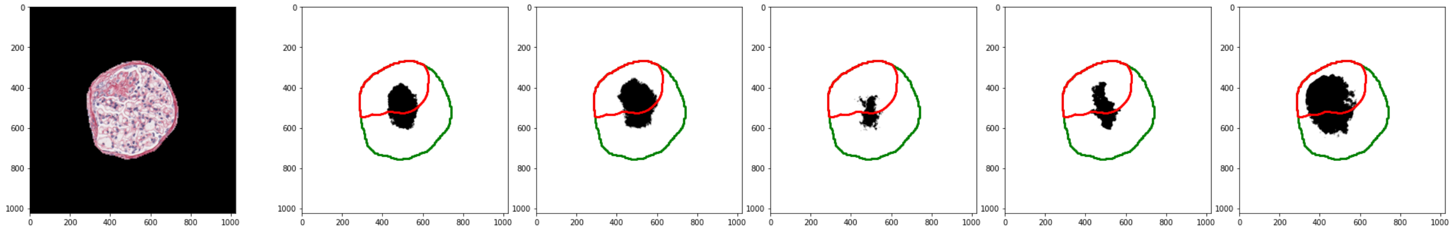

predicted area, %:	15.083	19.373	5.818	10.137	31.550
annotated area, %:	41.784	41.784	41.784	41.784	41.784
true label:	FSGS	FSGS	FSGS	FSGS	FSGS
predicted label:	Normal	Normal	Normal	Normal	Normal
id: 108	CV fold 1	CV fold 2	CV fold 3	CV fold 4	CV fold 5

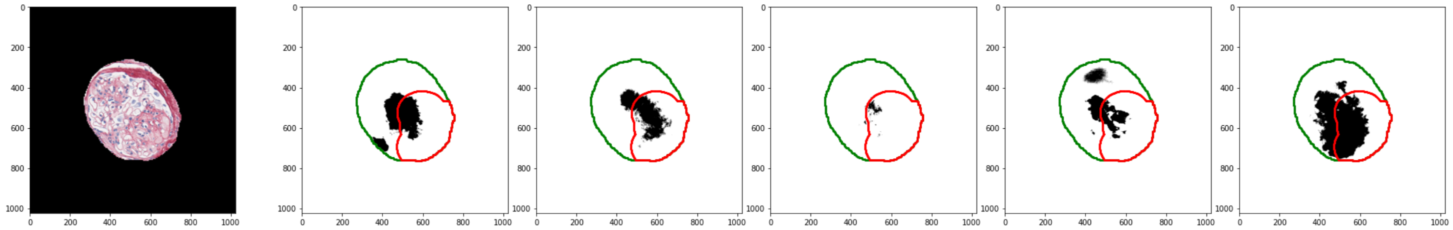

predicted area, %:	17.421	14.647	1.194	13.164	35.638
annotated area, %:	42.724	42.724	42.724	42.724	42.724
true label:	FSGS	FSGS	FSGS	FSGS	FSGS
predicted label:	FSGS	FSGS	FSGS	FSGS	FSGS
id: 109	CV fold 1	CV fold 2	CV fold 3	CV fold 4	CV fold 5

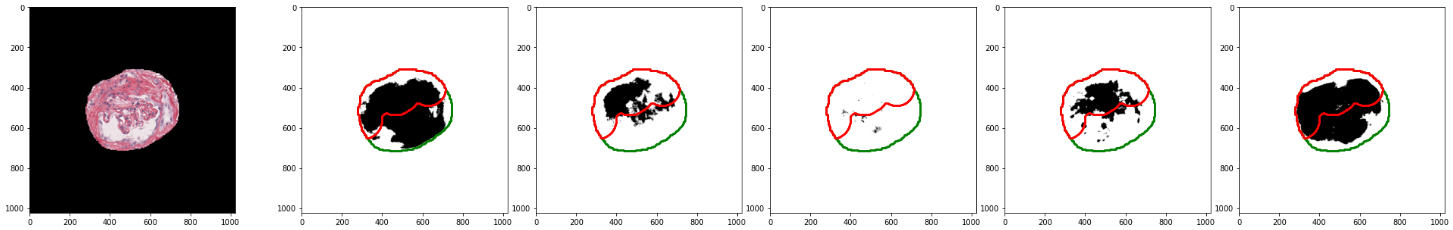

predicted area, %:	68.160	26.566	0.828	25.540	67.767
annotated area, %:	55.148	55.148	55.148	55.148	55.148
true label:	FSGS	FSGS	FSGS	FSGS	FSGS
predicted label:	FSGS	FSGS	FSGS	Sclerosed	FSGS
id: 110	CV fold 1	CV fold 2	CV fold 3	CV fold 4	CV fold 5

Supplementary Figure S2 (continued). Comparison of pattern quantification results per glomerular basis.

Red contour: annotated area @ 0.5 threshold **Green contour:** total area **Black fill:** predicted area @ 0.5 threshold

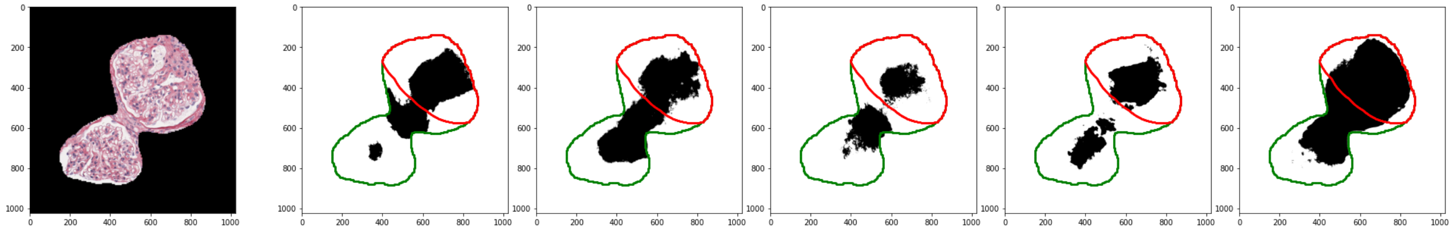

predicted area, %:	33.278	41.837	21.506	25.192	61.609
annotated area, %:	47.494	47.494	47.494	47.494	47.494
true label:	FSGS	FSGS	FSGS	FSGS	FSGS
predicted label:	Membranoproliferative	Membranoproliferative	Membranous	Membranous	Membranous
id: 111	CV fold 1	CV fold 2	CV fold 3	CV fold 4	CV fold 5

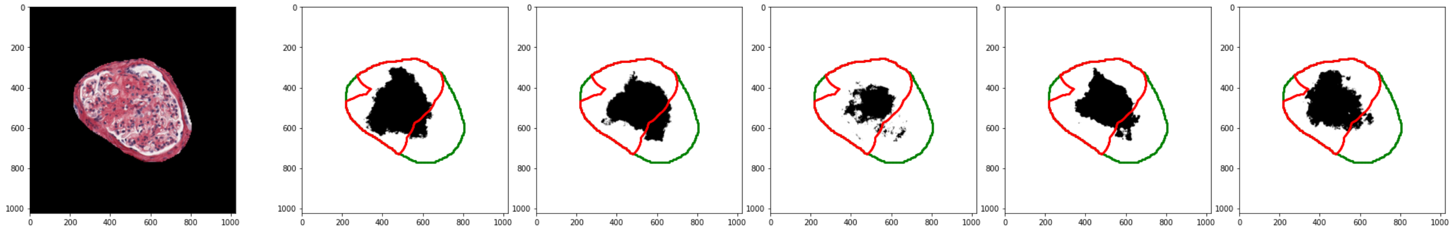

predicted area, %:	34.890	29.751	14.981	26.055	28.112
annotated area, %:	63.698	63.698	63.698	63.698	63.698
true label:	FSGS	FSGS	FSGS	FSGS	FSGS
predicted label:	Mesangioproliferative	Mesangioproliferative	Membranoproliferative	FSGS	Membranoproliferative
id: 112	CV fold 1	CV fold 2	CV fold 3	CV fold 4	CV fold 5

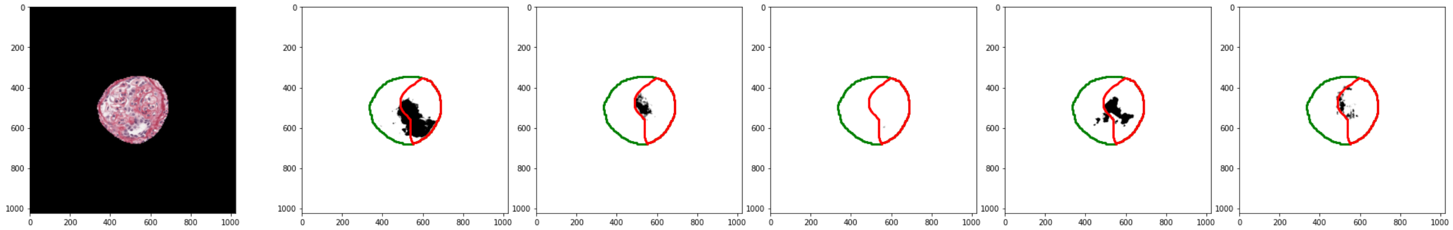

predicted area, %:	22.446	4.887	0.026	10.391	3.713
annotated area, %:	47.116	47.116	47.116	47.116	47.116
true label:	FSGS	FSGS	FSGS	FSGS	FSGS
predicted label:	FSGS	FSGS	FSGS	FSGS	Crescentic
id: 113	CV fold 1	CV fold 2	CV fold 3	CV fold 4	CV fold 5

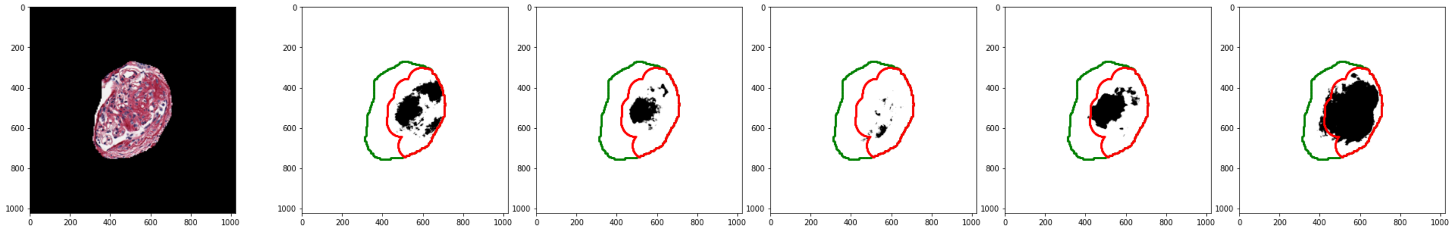

predicted area, %:	19.484	10.659	2.066	16.562	45.955
annotated area, %:	60.562	60.562	60.562	60.562	60.562
true label:	FSGS	FSGS	FSGS	FSGS	FSGS
predicted label:	FSGS	FSGS	FSGS	FSGS	FSGS
id: 114	CV fold 1	CV fold 2	CV fold 3	CV fold 4	CV fold 5

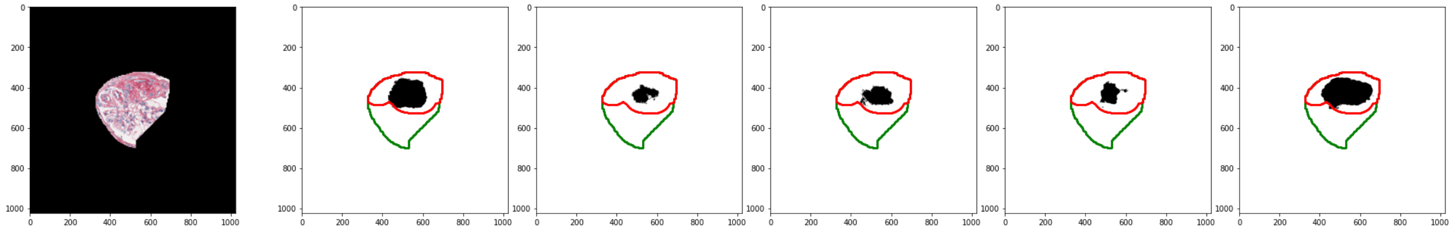

predicted area, %:	23.696	7.612	11.627	8.246	29.857
annotated area, %:	58.306	58.306	58.306	58.306	58.306
true label:	FSGS	FSGS	FSGS	FSGS	FSGS
predicted label:	FSGS	FSGS	FSGS	FSGS	FSGS
id: 115	CV fold 1	CV fold 2	CV fold 3	CV fold 4	CV fold 5

Supplementary Figure S2 (continued). Comparison of pattern quantification results per glomerular basis.

Red contour: annotated area @ 0.5 threshold **Green contour:** total area **Black fill:** predicted area @ 0.5 threshold

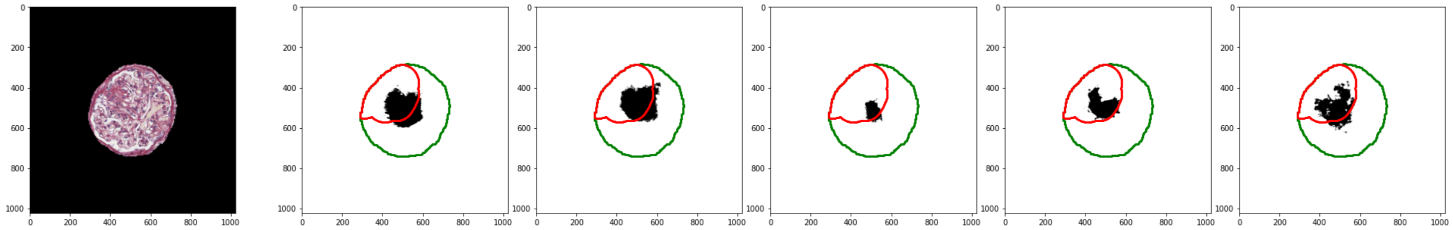

predicted area, %:	17.738	18.110	4.202	9.715	15.718
annotated area, %:	38.821	38.821	38.821	38.821	38.821
true label:	FSGS	FSGS	FSGS	FSGS	FSGS
predicted label:	Endocapillary	Mesangioproliferative	Endocapillary	FSGS	Endocapillary
id: 116	CV fold 1	CV fold 2	CV fold 3	CV fold 4	CV fold 5

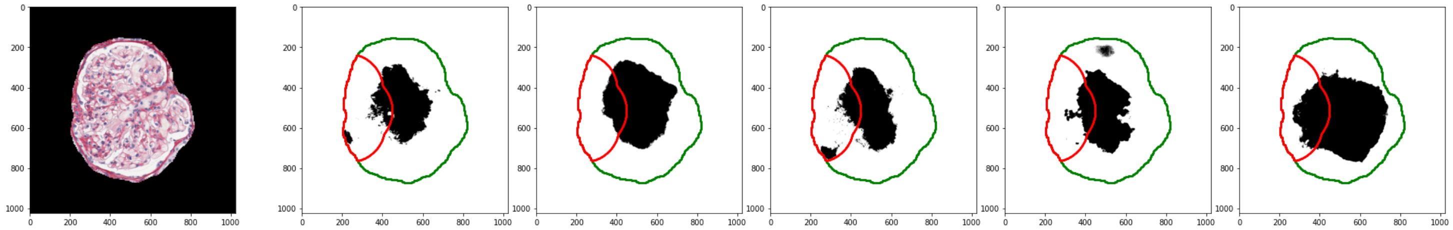

predicted area, %:	22.514	33.064	23.130	24.722	43.954
annotated area, %:	26.729	26.729	26.729	26.729	26.729
true label:	FSGS	FSGS	FSGS	FSGS	FSGS
predicted label:	Normal	Hypertrophy	Normal	Hypertrophy	Hypertrophy
id: 117	CV fold 1	CV fold 2	CV fold 3	CV fold 4	CV fold 5

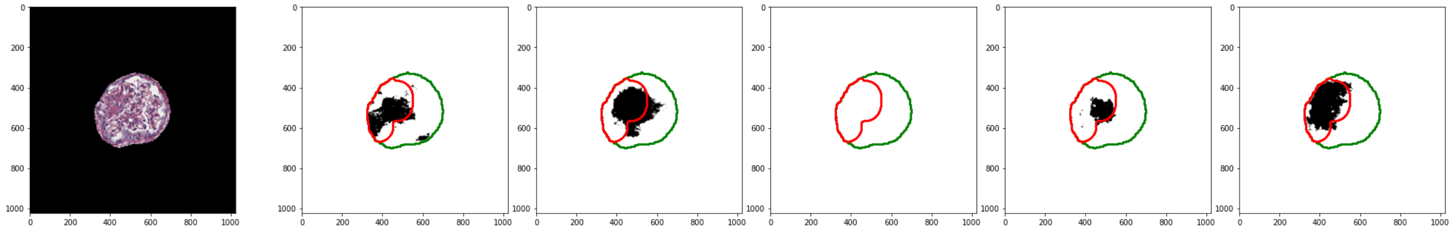

predicted area, %:	24.217	33.494	0.000	11.099	31.931
annotated area, %:	43.919	43.919	43.919	43.919	43.919
true label:	FSGS	FSGS	FSGS	FSGS	FSGS
predicted label:	Mesangioproliferative	Mesangioproliferative	Mesangioproliferative	Mesangioproliferative	Mesangioproliferative
id: 118	CV fold 1	CV fold 2	CV fold 3	CV fold 4	CV fold 5

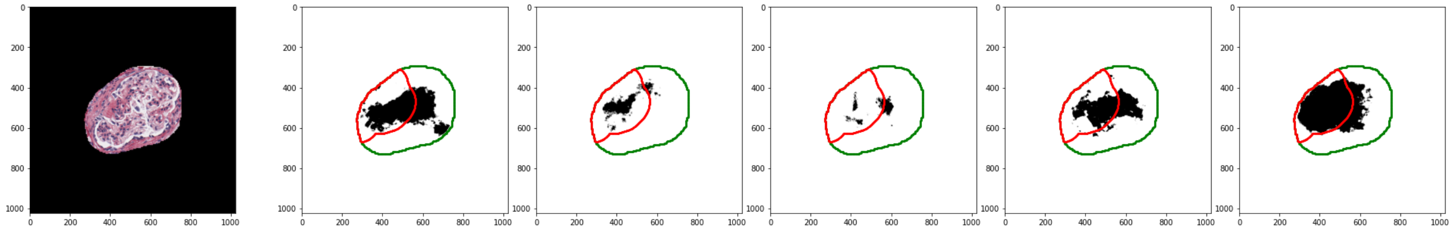

predicted area, %:	31.198	8.395	4.211	23.956	43.357
annotated area, %:	40.089	40.089	40.089	40.089	40.089
true label:	FSGS	FSGS	FSGS	FSGS	FSGS
predicted label:	FSGS	FSGS	Crescentic	Crescentic	Endocapillary
id: 119	CV fold 1	CV fold 2	CV fold 3	CV fold 4	CV fold 5

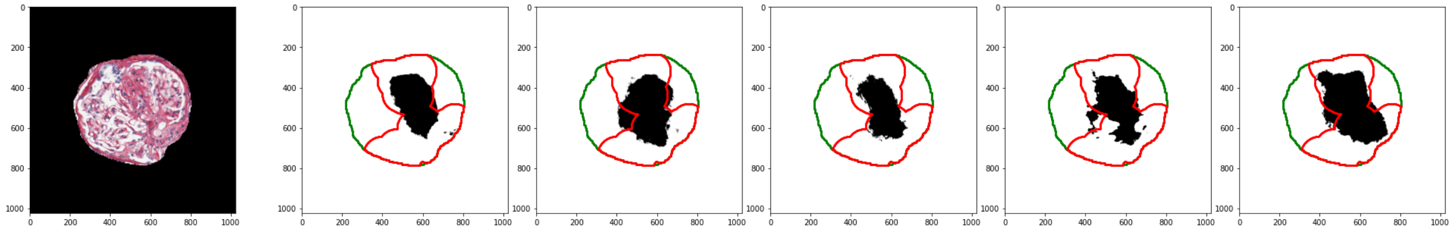

predicted area, %:	20.531	28.230	18.417	22.523	32.911
annotated area, %:	64.220	64.220	64.220	64.220	64.220
true label:	FSGS	FSGS	FSGS	FSGS	FSGS
predicted label:	FSGS	Membranoproliferative	Membranoproliferative	FSGS	Membranoproliferative
id: 120	CV fold 1	CV fold 2	CV fold 3	CV fold 4	CV fold 5

Supplementary Figure S2 (continued). Comparison of pattern quantification results per glomerular basis.

Red contour: annotated area @ 0.5 threshold **Green contour:** total area **Black fill:** predicted area @ 0.5 threshold

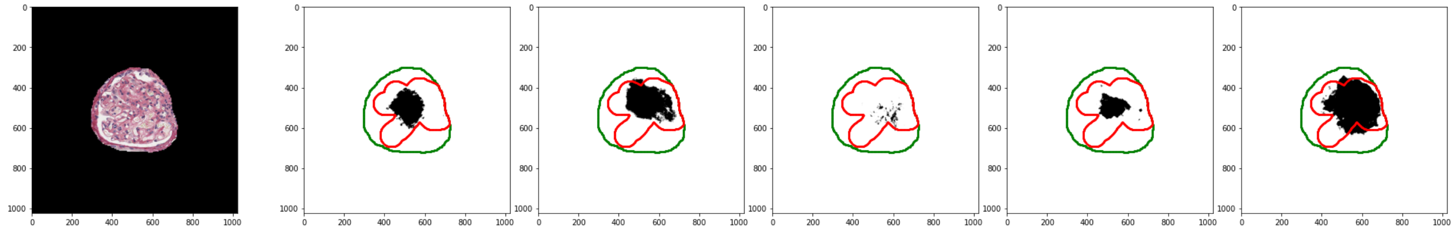

predicted area, %:	15.175	23.987	1.753	8.996	38.632
annotated area, %:	57.713	57.713	57.713	57.713	57.713
true label:	Mesangioproliferative	Mesangioproliferative	Mesangioproliferative	Mesangioproliferative	Mesangioproliferative
predicted label:	Mesangioproliferative	Mesangioproliferative	Mesangioproliferative	FSGS	Mesangioproliferative
id: 121	CV fold 1	CV fold 2	CV fold 3	CV fold 4	CV fold 5

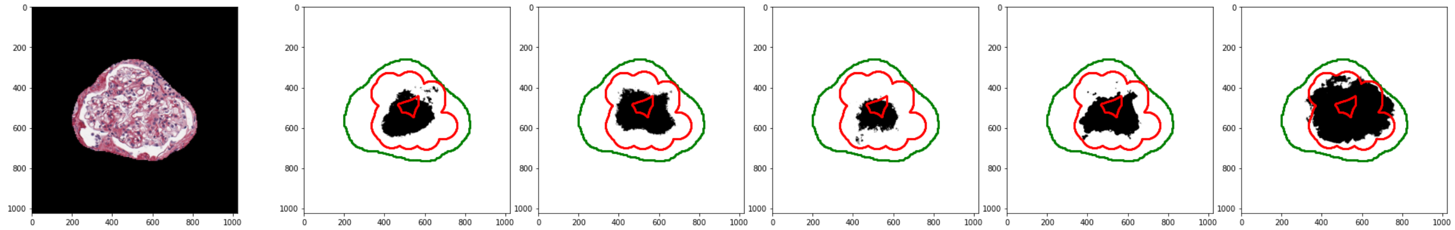

predicted area, %:	18.317	21.882	11.341	20.459	46.092
annotated area, %:	53.571	53.571	53.571	53.571	53.571
true label:	Mesangioproliferative	Mesangioproliferative	Mesangioproliferative	Mesangioproliferative	Mesangioproliferative
predicted label:	Mesangioproliferative	Mesangioproliferative	Mesangioproliferative	Mesangioproliferative	Mesangioproliferative
id: 122	CV fold 1	CV fold 2	CV fold 3	CV fold 4	CV fold 5

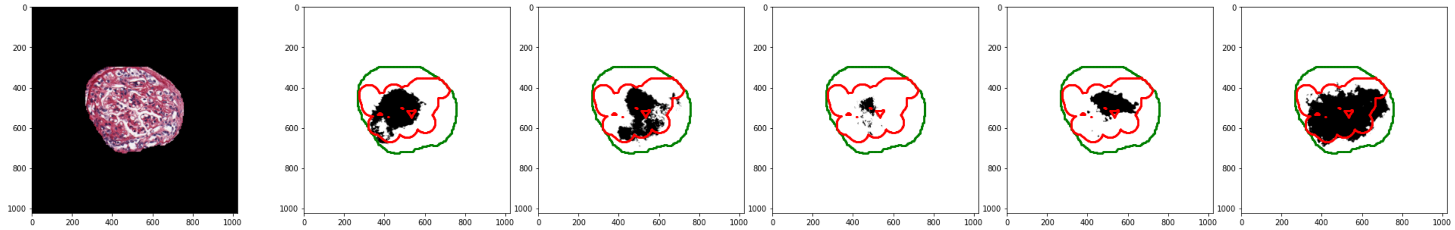

predicted area, %:	23.458	21.883	3.212	11.400	47.660
annotated area, %:	56.873	56.873	56.873	56.873	56.873
true label:	Mesangioproliferative	Mesangioproliferative	Mesangioproliferative	Mesangioproliferative	Mesangioproliferative
predicted label:	Mesangioproliferative	Mesangioproliferative	Mesangioproliferative	Mesangioproliferative	Mesangioproliferative
id: 123	CV fold 1	CV fold 2	CV fold 3	CV fold 4	CV fold 5

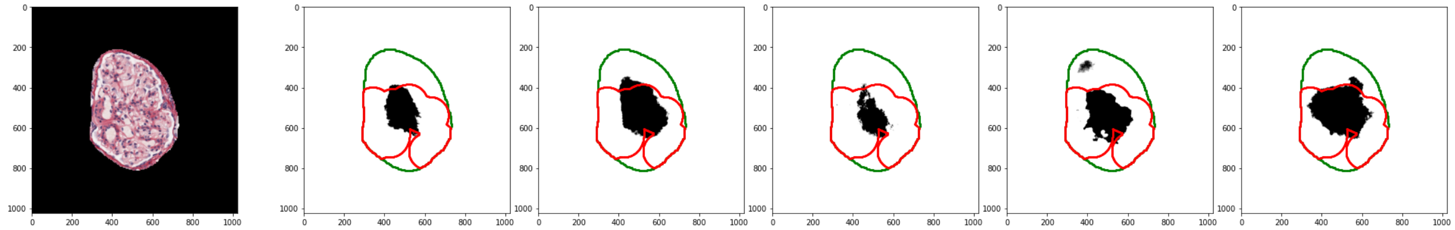

predicted area, %:	14.637	24.331	10.851	21.239	28.809
annotated area, %:	65.038	65.038	65.038	65.038	65.038
true label:	Mesangioproliferative	Mesangioproliferative	Mesangioproliferative	Mesangioproliferative	Mesangioproliferative
predicted label:	Membranoproliferative	Hypertrophy	FSGS	FSGS	Endocapillary
id: 124	CV fold 1	CV fold 2	CV fold 3	CV fold 4	CV fold 5

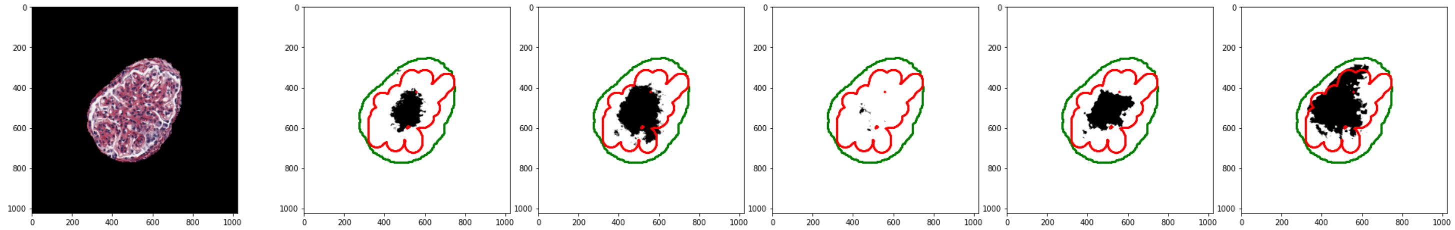

predicted area, %:	12.494	24.611	0.469	16.324	35.215
annotated area, %:	61.430	61.430	61.430	61.430	61.430
true label:	Mesangioproliferative	Mesangioproliferative	Mesangioproliferative	Mesangioproliferative	Mesangioproliferative
predicted label:	Mesangioproliferative	Mesangioproliferative	Membranoproliferative	Mesangioproliferative	Membranoproliferative
id: 125	CV fold 1	CV fold 2	CV fold 3	CV fold 4	CV fold 5

Supplementary Figure S2 (continued). Comparison of pattern quantification results per glomerular basis.

Red contour: annotated area @ 0.5 threshold **Green contour:** total area **Black fill:** predicted area @ 0.5 threshold

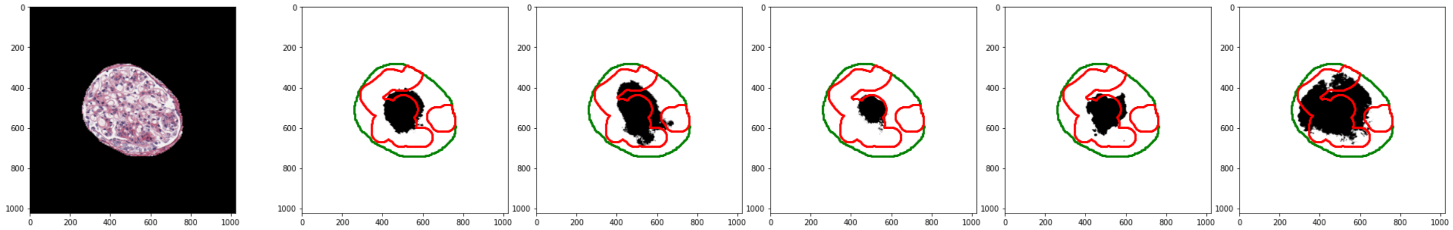

predicted area, %:	18.820	28.399	9.066	16.112	47.886
annotated area, %:	58.926	58.926	58.926	58.926	58.926
true label:	Mesangioproliferative	Mesangioproliferative	Mesangioproliferative	Mesangioproliferative	Mesangioproliferative
predicted label:	Mesangioproliferative	Mesangioproliferative	Mesangioproliferative	Mesangioproliferative	Mesangioproliferative
id: 126	CV fold 1	CV fold 2	CV fold 3	CV fold 4	CV fold 5

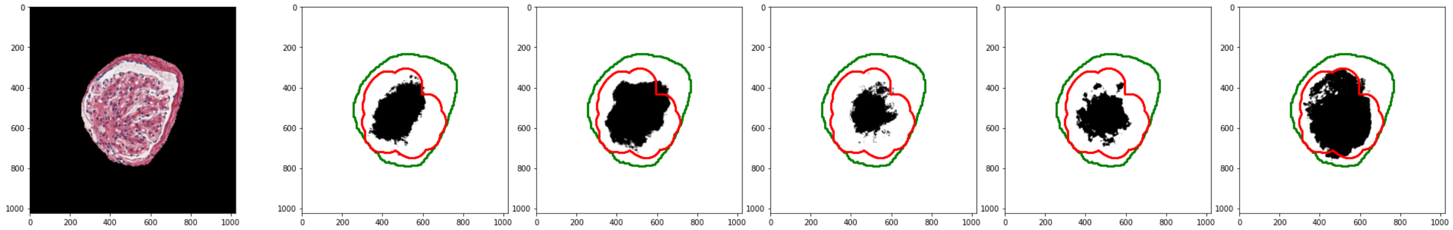

predicted area, %:	26.109	36.658	17.235	21.789	48.253
annotated area, %:	65.090	65.090	65.090	65.090	65.090
true label:	Mesangioproliferative	Mesangioproliferative	Mesangioproliferative	Mesangioproliferative	Mesangioproliferative
predicted label:	FSGS	Mesangioproliferative	Membranoproliferative	Membranoproliferative	Membranoproliferative
id: 127	CV fold 1	CV fold 2	CV fold 3	CV fold 4	CV fold 5

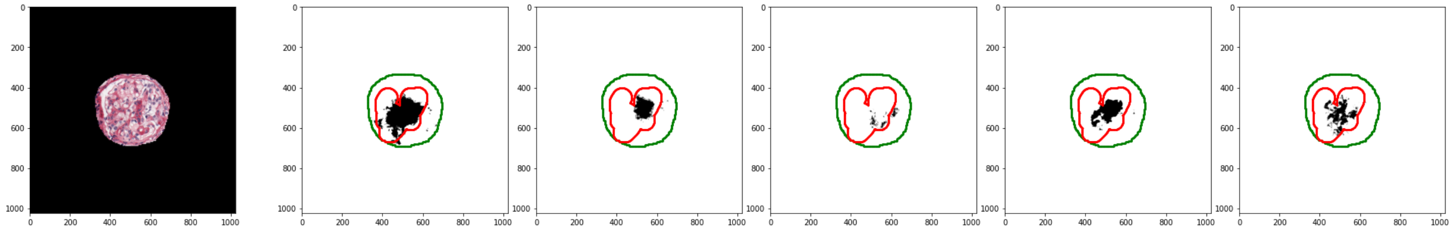

predicted area, %:	23.582	7.589	1.847	10.575	9.517
annotated area, %:	47.445	47.445	47.445	47.445	47.445
true label:	Mesangioproliferative	Mesangioproliferative	Mesangioproliferative	Mesangioproliferative	Mesangioproliferative
predicted label:	Mesangioproliferative	Mesangioproliferative	Mesangioproliferative	FSGS	Mesangioproliferative
id: 128	CV fold 1	CV fold 2	CV fold 3	CV fold 4	CV fold 5

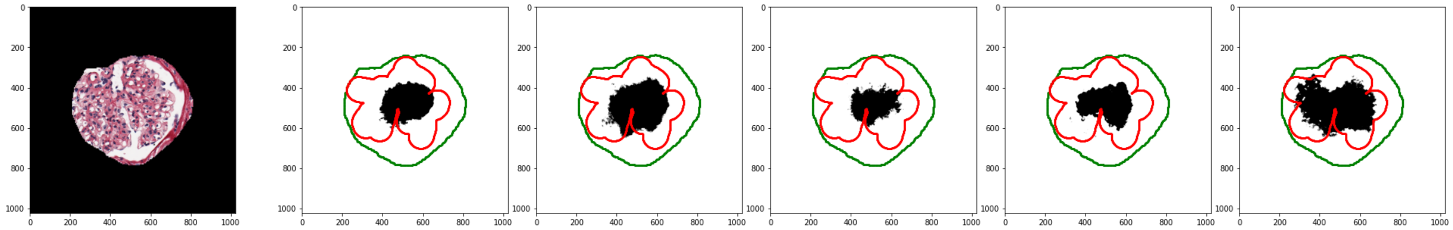

predicted area, %:	17.614	25.422	13.717	17.332	33.515
annotated area, %:	60.234	60.234	60.234	60.234	60.234
true label:	Mesangioproliferative	Mesangioproliferative	Mesangioproliferative	Mesangioproliferative	Mesangioproliferative
predicted label:	Mesangioproliferative	Mesangioproliferative	Mesangioproliferative	Membranoproliferative	Mesangioproliferative
id: 129	CV fold 1	CV fold 2	CV fold 3	CV fold 4	CV fold 5

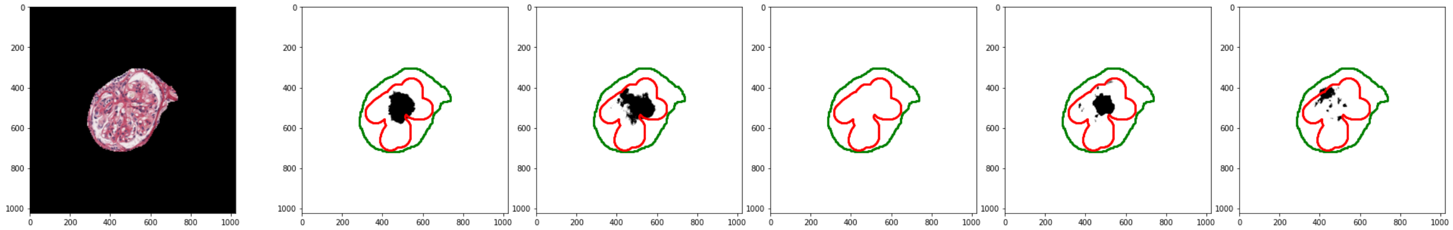

predicted area, %:	12.118	14.429	0.000	7.321	4.460
annotated area, %:	52.924	52.924	52.924	52.924	52.924
true label:	Mesangioproliferative	Mesangioproliferative	Mesangioproliferative	Mesangioproliferative	Mesangioproliferative
predicted label:	FSGS	Mesangioproliferative	FSGS	FSGS	FSGS
id: 130	CV fold 1	CV fold 2	CV fold 3	CV fold 4	CV fold 5

Supplementary Figure S2 (continued). Comparison of pattern quantification results per glomerular basis.

Red contour: annotated area @ 0.5 threshold **Green contour:** total area **Black fill:** predicted area @ 0.5 threshold

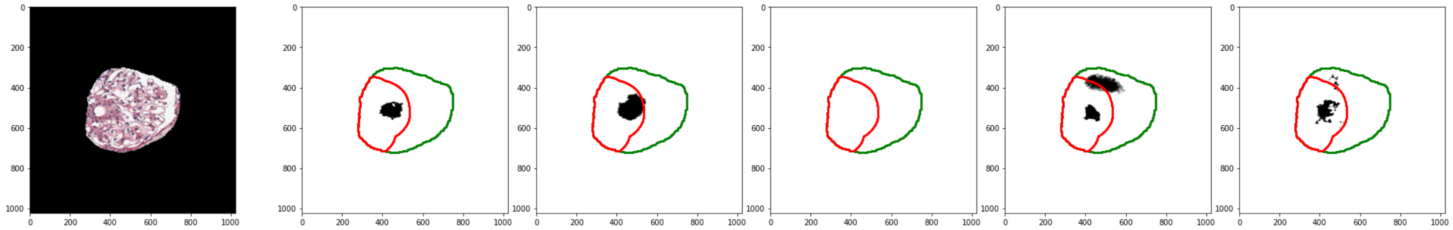

predicted area, %:	4.471	8.152	0.000	9.455	4.801
annotated area, %:	45.503	45.503	45.503	45.503	45.503
true label:	Mesangioproliferative	Mesangioproliferative	Mesangioproliferative	Mesangioproliferative	Mesangioproliferative
predicted label:	Mesangioproliferative	Mesangioproliferative	Mesangioproliferative	Membranous	Mesangioproliferative
id: 131	CV fold 1	CV fold 2	CV fold 3	CV fold 4	CV fold 5

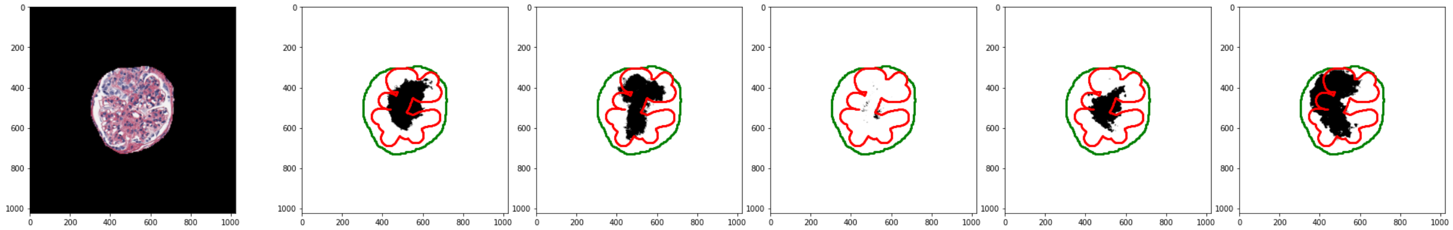

predicted area, %:	23.842	27.098	0.954	15.315	36.547
annotated area, %:	59.250	59.250	59.250	59.250	59.250
true label:	Mesangioproliferative	Mesangioproliferative	Mesangioproliferative	Mesangioproliferative	Mesangioproliferative
predicted label:	Mesangioproliferative	Mesangioproliferative	Mesangioproliferative	Mesangioproliferative	Mesangioproliferative
id: 132	CV fold 1	CV fold 2	CV fold 3	CV fold 4	CV fold 5

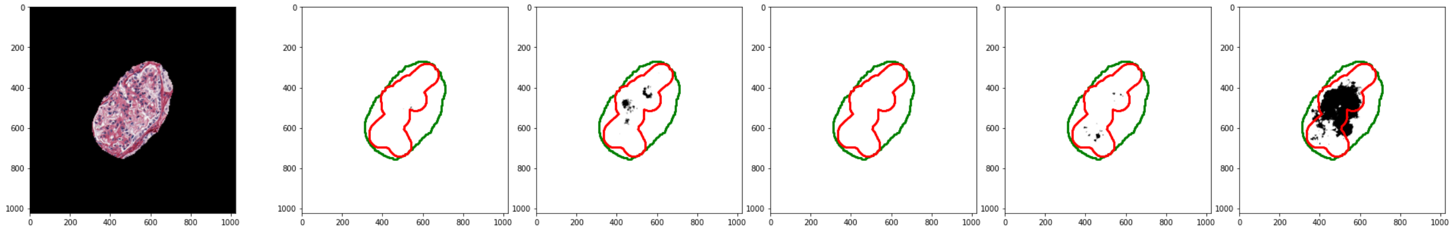

predicted area, %:	0.049	2.430	0.000	0.705	30.872
annotated area, %:	59.808	59.808	59.808	59.808	59.808
true label:	Mesangioproliferative	Mesangioproliferative	Mesangioproliferative	Mesangioproliferative	Mesangioproliferative
predicted label:	FSGS	FSGS	FSGS	FSGS	FSGS
id: 133	CV fold 1	CV fold 2	CV fold 3	CV fold 4	CV fold 5

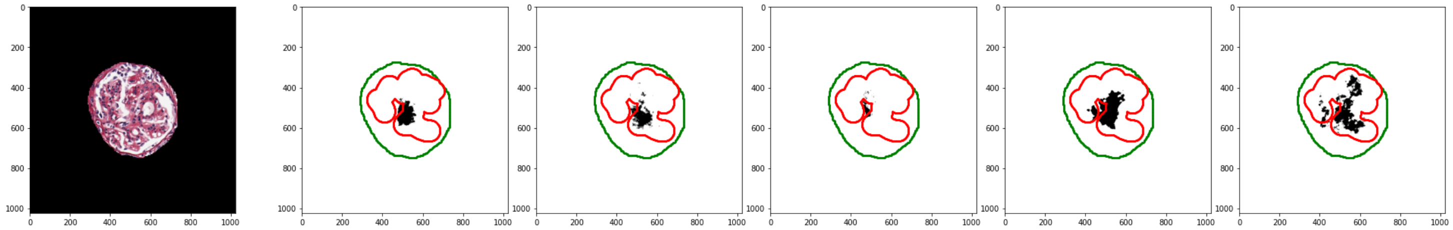

predicted area, %:	5.129	4.946	1.191	10.369	12.252
annotated area, %:	54.909	54.909	54.909	54.909	54.909
true label:	Mesangioproliferative	Mesangioproliferative	Mesangioproliferative	Mesangioproliferative	Mesangioproliferative
predicted label:	Mesangioproliferative	Mesangioproliferative	Mesangioproliferative	Mesangioproliferative	Mesangioproliferative
id: 134	CV fold 1	CV fold 2	CV fold 3	CV fold 4	CV fold 5

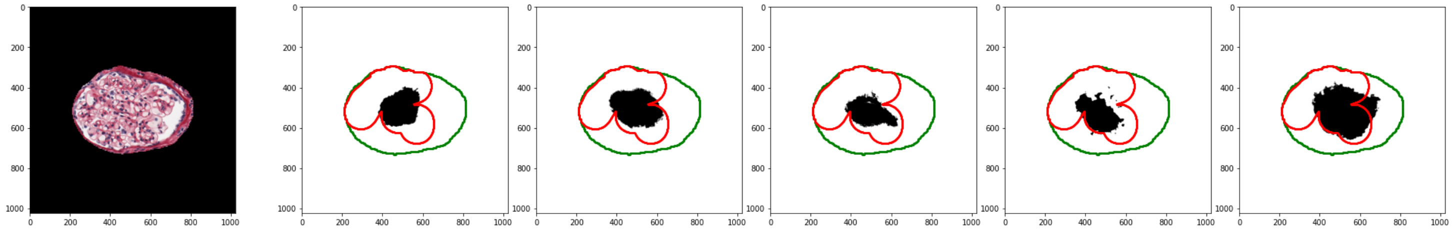

predicted area, %:	14.837	19.298	13.419	14.116	31.212
annotated area, %:	58.131	58.131	58.131	58.131	58.131
true label:	Mesangioproliferative	Mesangioproliferative	Mesangioproliferative	Mesangioproliferative	Mesangioproliferative
predicted label:	Mesangioproliferative	Mesangioproliferative	Mesangioproliferative	Normal	Mesangioproliferative
id: 135	CV fold 1	CV fold 2	CV fold 3	CV fold 4	CV fold 5

Supplementary Figure S2 (continued). Comparison of pattern quantification results per glomerular basis.

Red contour: annotated area @ 0.5 threshold **Green contour:** total area **Black fill:** predicted area @ 0.5 threshold

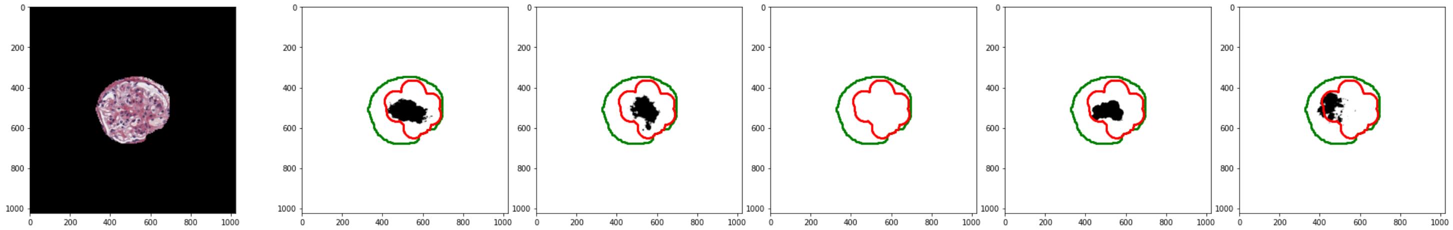

predicted area, %:	17.234	13.682	0.000	12.274	11.801
annotated area, %:	56.189	56.189	56.189	56.189	56.189
true label:	Mesangioproliferative	Mesangioproliferative	Mesangioproliferative	Mesangioproliferative	Mesangioproliferative
predicted label:	Mesangioproliferative	Mesangioproliferative	Mesangioproliferative	FSGS	Mesangioproliferative
id: 136	CV fold 1	CV fold 2	CV fold 3	CV fold 4	CV fold 5

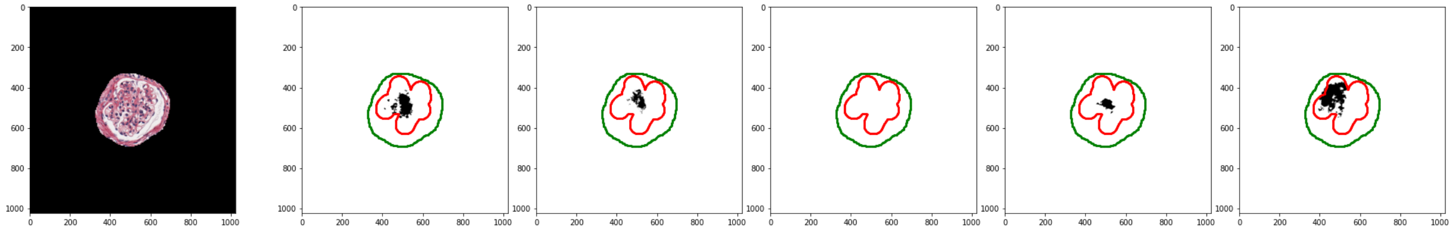

predicted area, %:	7.038	3.643	0.000	2.512	12.636
annotated area, %:	48.641	48.641	48.641	48.641	48.641
true label:	Mesangioproliferative	Mesangioproliferative	Mesangioproliferative	Mesangioproliferative	Mesangioproliferative
predicted label:	Mesangioproliferative	Mesangioproliferative	Mesangioproliferative	Mesangioproliferative	Mesangioproliferative
id: 137	CV fold 1	CV fold 2	CV fold 3	CV fold 4	CV fold 5

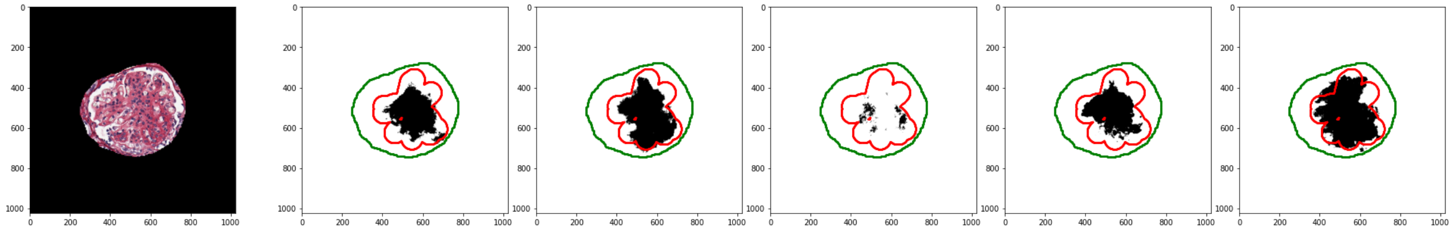

predicted area, %:	24.408	33.347	3.447	24.078	42.743
annotated area, %:	51.462	51.462	51.462	51.462	51.462
true label:	Mesangioproliferative	Mesangioproliferative	Mesangioproliferative	Mesangioproliferative	Mesangioproliferative
predicted label:	Mesangioproliferative	Mesangioproliferative	Mesangioproliferative	Mesangioproliferative	Mesangioproliferative
id: 138	CV fold 1	CV fold 2	CV fold 3	CV fold 4	CV fold 5

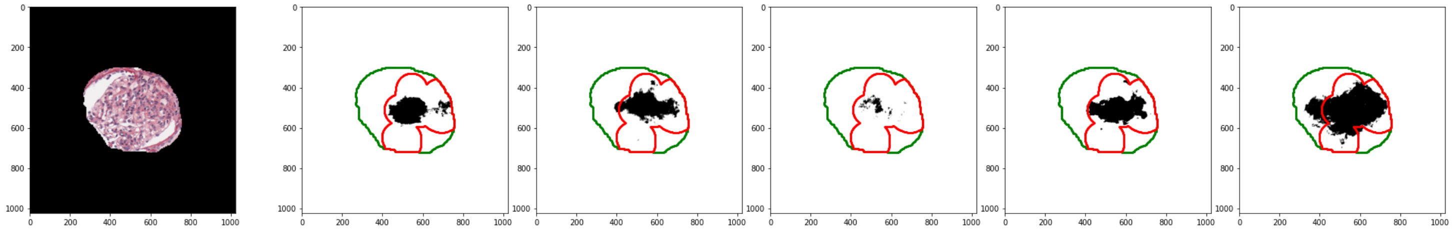

predicted area, %:	13.484	17.221	2.379	17.694	39.419
annotated area, %:	58.337	58.337	58.337	58.337	58.337
true label:	Mesangioproliferative	Mesangioproliferative	Mesangioproliferative	Mesangioproliferative	Mesangioproliferative
predicted label:	Mesangioproliferative	Mesangioproliferative	Endocapillary	Crescentic	Endocapillary
id: 139	CV fold 1	CV fold 2	CV fold 3	CV fold 4	CV fold 5

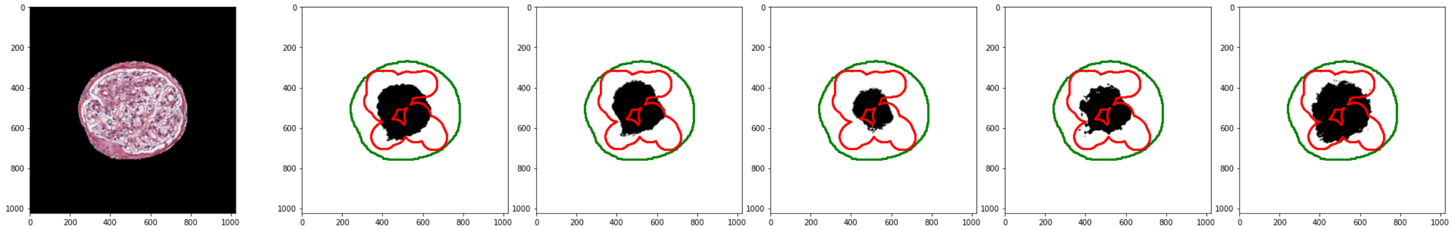

predicted area, %:	27.020	24.204	13.958	20.655	32.097
annotated area, %:	55.045	55.045	55.045	55.045	55.045
true label:	Mesangioproliferative	Mesangioproliferative	Mesangioproliferative	Mesangioproliferative	Mesangioproliferative
predicted label:	Membranous	Membranous	Membranous	Membranous	Membranous
id: 140	CV fold 1	CV fold 2	CV fold 3	CV fold 4	CV fold 5

Supplementary Figure S2 (continued). Comparison of pattern quantification results per glomerular basis.

Red contour: annotated area @ 0.5 threshold **Green contour:** total area **Black fill:** predicted area @ 0.5 threshold

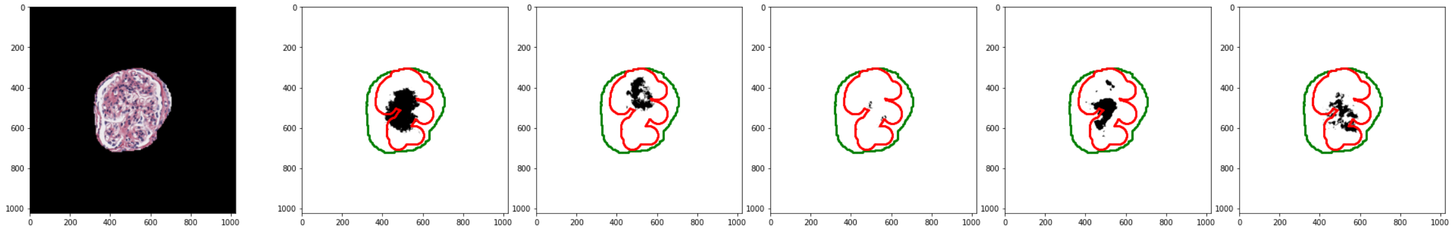

id: 141	CV fold 1	CV fold 2	CV fold 3	CV fold 4	CV fold 5
predicted area, %:	19.438	8.505	0.479	9.264	8.224
annotated area, %:	60.925	60.925	60.925	60.925	60.925
true label:	Mesangioproliferative	Mesangioproliferative	Mesangioproliferative	Mesangioproliferative	Mesangioproliferative
predicted label:	Mesangioproliferative	Mesangioproliferative	Mesangioproliferative	Mesangioproliferative	Mesangioproliferative

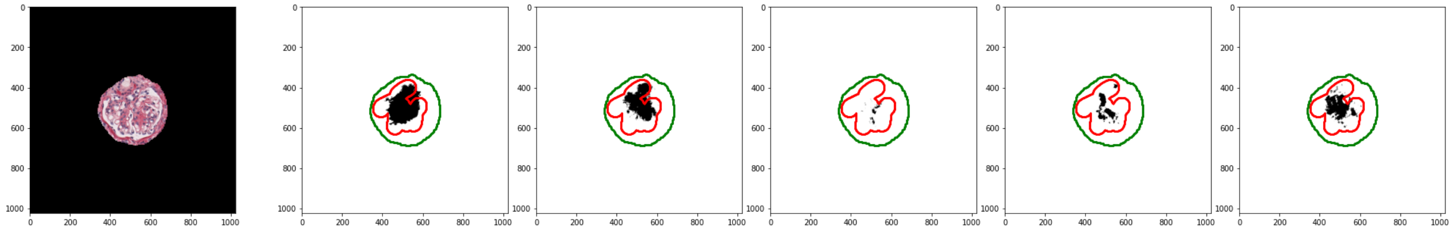

id: 142	CV fold 1	CV fold 2	CV fold 3	CV fold 4	CV fold 5
predicted area, %:	25.465	18.973	1.240	5.758	13.032
annotated area, %:	48.202	48.202	48.202	48.202	48.202
true label:	Mesangioproliferative	Mesangioproliferative	Mesangioproliferative	Mesangioproliferative	Mesangioproliferative
predicted label:	Mesangioproliferative	Mesangioproliferative	Mesangioproliferative	Mesangioproliferative	Mesangioproliferative

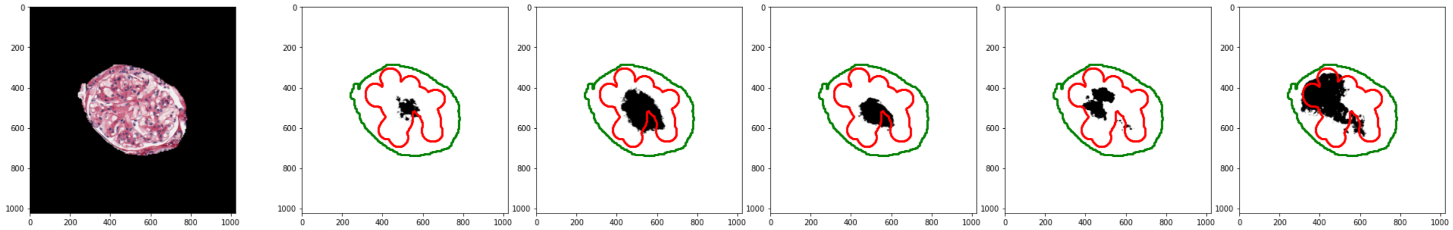

id: 143	CV fold 1	CV fold 2	CV fold 3	CV fold 4	CV fold 5
predicted area, %:	4.124	17.073	9.406	8.078	25.546
annotated area, %:	51.627	51.627	51.627	51.627	51.627
true label:	Mesangioproliferative	Mesangioproliferative	Mesangioproliferative	Mesangioproliferative	Mesangioproliferative
predicted label:	FSGS	Mesangioproliferative	FSGS	FSGS	FSGS

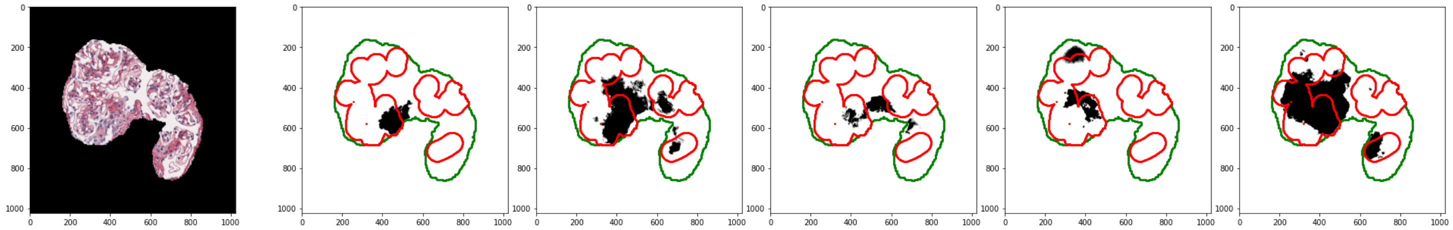

id: 144	CV fold 1	CV fold 2	CV fold 3	CV fold 4	CV fold 5
predicted area, %:	5.257	20.166	5.347	7.557	31.193
annotated area, %:	55.513	55.513	55.513	55.513	55.513
true label:	Mesangioproliferative	Mesangioproliferative	Mesangioproliferative	Mesangioproliferative	Mesangioproliferative
predicted label:	Membranoproliferative	Membranoproliferative	Membranoproliferative	Membranoproliferative	Membranoproliferative

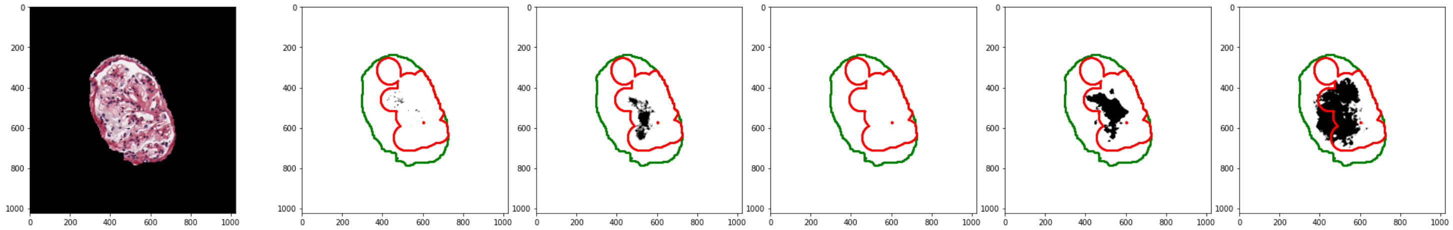

id: 145	CV fold 1	CV fold 2	CV fold 3	CV fold 4	CV fold 5
predicted area, %:	0.344	5.159	0.002	11.192	29.123
annotated area, %:	57.719	57.719	57.719	57.719	57.719
true label:	Mesangioproliferative	Mesangioproliferative	Mesangioproliferative	Mesangioproliferative	Mesangioproliferative
predicted label:	Mesangioproliferative	Mesangioproliferative	Mesangioproliferative	Mesangioproliferative	Mesangioproliferative

Supplementary Figure S2 (continued). Comparison of pattern quantification results per glomerular basis.

Red contour: annotated area @ 0.5 threshold **Green contour:** total area **Black fill:** predicted area @ 0.5 threshold

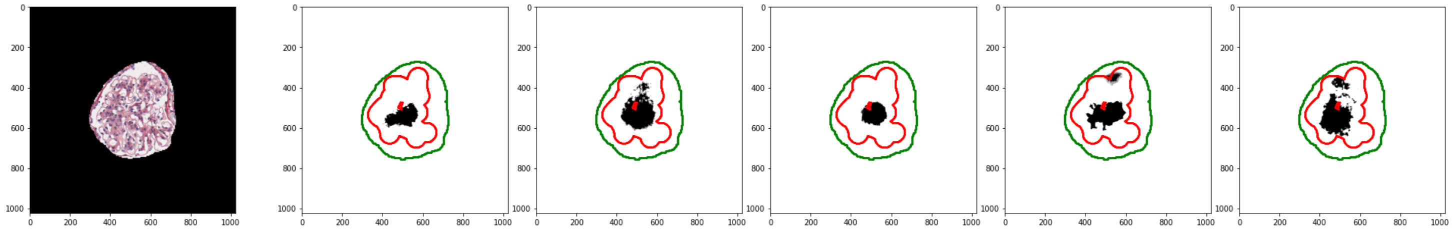

predicted area, %:	7.517	14.166	6.961	12.087	15.367
annotated area, %:	54.597	54.597	54.597	54.597	54.597
true label:	Mesangioproliferative	Mesangioproliferative	Mesangioproliferative	Mesangioproliferative	Mesangioproliferative
predicted label:	Mesangioproliferative	Mesangioproliferative	Mesangioproliferative	Mesangioproliferative	Mesangioproliferative
id: 146	CV fold 1	CV fold 2	CV fold 3	CV fold 4	CV fold 5

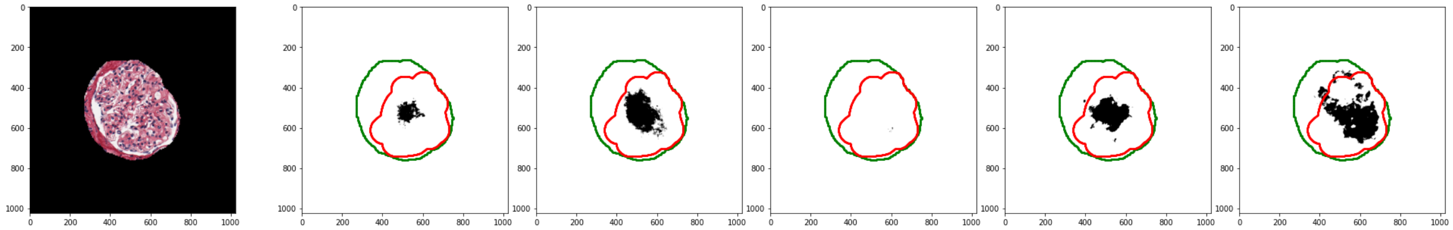

predicted area, %:	4.386	13.758	0.028	11.491	22.927
annotated area, %:	64.827	64.827	64.827	64.827	64.827
true label:	Mesangioproliferative	Mesangioproliferative	Mesangioproliferative	Mesangioproliferative	Mesangioproliferative
predicted label:	Mesangioproliferative	Mesangioproliferative	Mesangioproliferative	FSGS	Mesangioproliferative
id: 147	CV fold 1	CV fold 2	CV fold 3	CV fold 4	CV fold 5

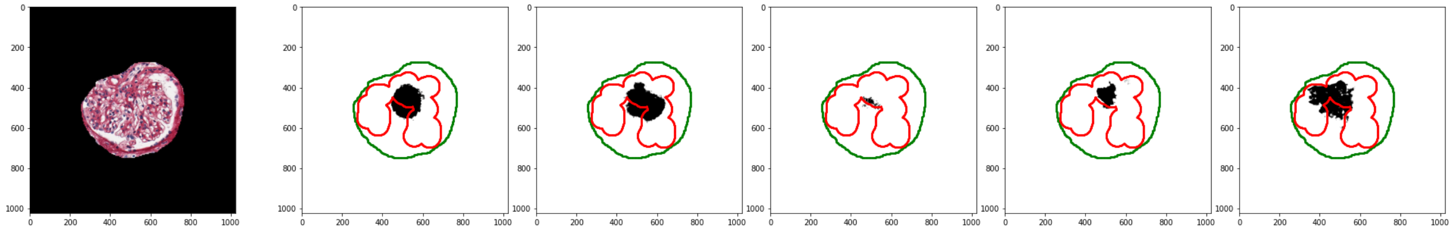

predicted area, %:	10.975	13.476	0.901	4.214	15.363
annotated area, %:	53.596	53.596	53.596	53.596	53.596
true label:	Mesangioproliferative	Mesangioproliferative	Mesangioproliferative	Mesangioproliferative	Mesangioproliferative
predicted label:	Mesangioproliferative	Mesangioproliferative	Mesangioproliferative	FSGS	Mesangioproliferative
id: 148	CV fold 1	CV fold 2	CV fold 3	CV fold 4	CV fold 5

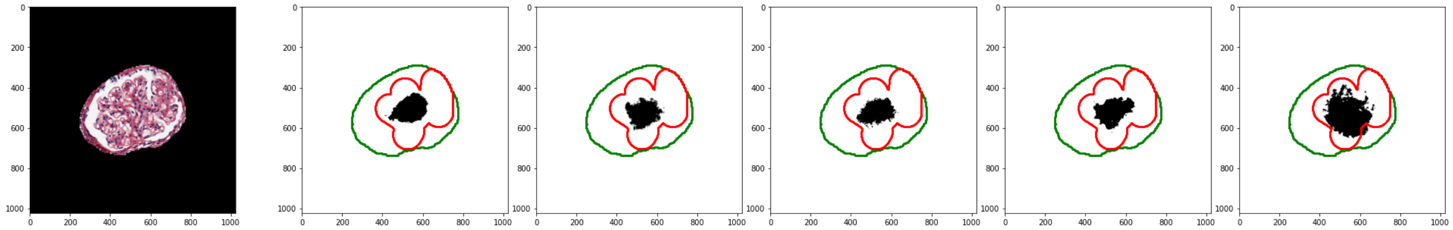

predicted area, %:	10.966	10.724	10.071	11.410	22.377
annotated area, %:	54.841	54.841	54.841	54.841	54.841
true label:	Mesangioproliferative	Mesangioproliferative	Mesangioproliferative	Mesangioproliferative	Mesangioproliferative
predicted label:	Mesangioproliferative	Mesangioproliferative	Mesangioproliferative	Mesangioproliferative	Mesangioproliferative
id: 149	CV fold 1	CV fold 2	CV fold 3	CV fold 4	CV fold 5

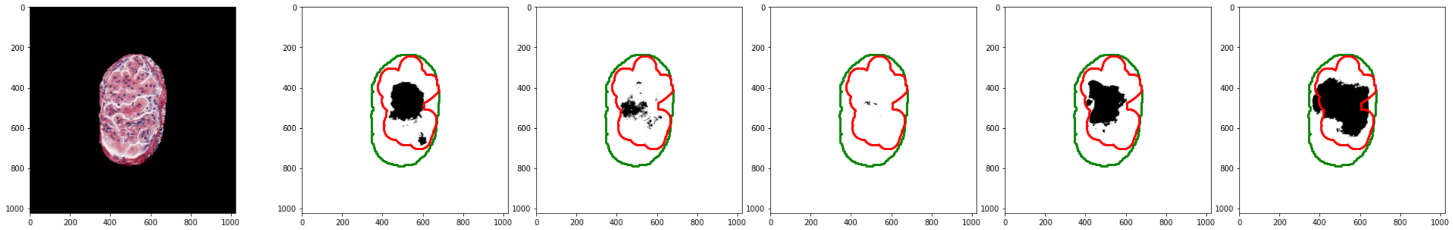

predicted area, %:	18.173	5.862	0.233	19.183	36.182
annotated area, %:	63.699	63.699	63.699	63.699	63.699
true label:	Mesangioproliferative	Mesangioproliferative	Mesangioproliferative	Mesangioproliferative	Mesangioproliferative
predicted label:	Membranoproliferative	Membranoproliferative	Membranoproliferative	Membranoproliferative	Membranoproliferative
id: 150	CV fold 1	CV fold 2	CV fold 3	CV fold 4	CV fold 5

Supplementary Figure S2 (continued). Comparison of pattern quantification results per glomerular basis.

Red contour: annotated area @ 0.5 threshold **Green contour:** total area **Black fill:** predicted area @ 0.5 threshold

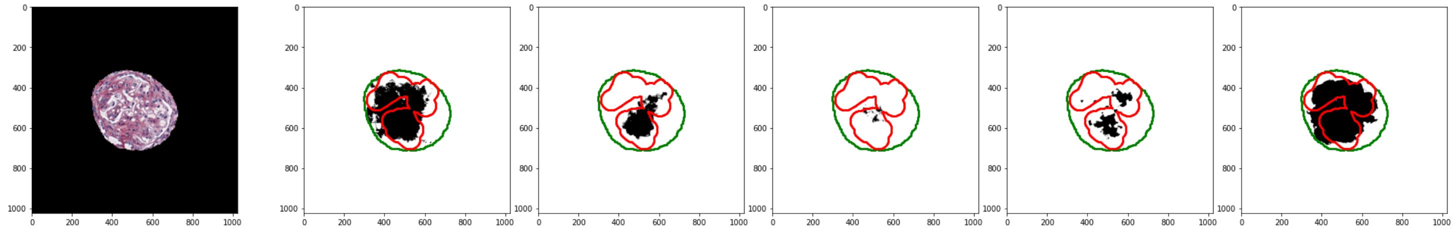

predicted area, %:	47.829	16.704	1.445	10.425	62.297
annotated area, %:	58.210	58.210	58.210	58.210	58.210
true label:	Mesangioproliferative	Mesangioproliferative	Mesangioproliferative	Mesangioproliferative	Mesangioproliferative
predicted label:	FSGS	Mesangioproliferative	FSGS	FSGS	FSGS
id: 151	CV fold 1	CV fold 2	CV fold 3	CV fold 4	CV fold 5

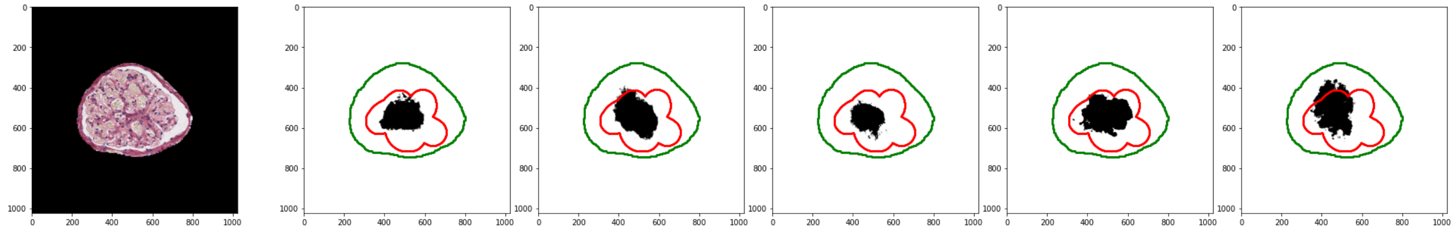

predicted area, %:	13.627	17.738	9.896	18.815	19.962
annotated area, %:	43.151	43.151	43.151	43.151	43.151
true label:	Mesangioproliferative	Mesangioproliferative	Mesangioproliferative	Mesangioproliferative	Mesangioproliferative
predicted label:	FSGS	Mesangioproliferative	Mesangioproliferative	FSGS	Mesangioproliferative
id: 152	CV fold 1	CV fold 2	CV fold 3	CV fold 4	CV fold 5

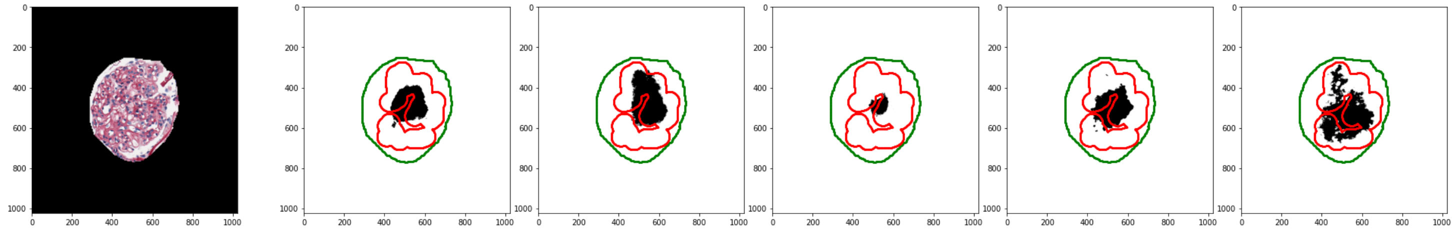

predicted area, %:	14.606	20.614	3.646	15.919	27.720
annotated area, %:	55.370	55.370	55.370	55.370	55.370
true label:	Mesangioproliferative	Mesangioproliferative	Mesangioproliferative	Mesangioproliferative	Mesangioproliferative
predicted label:	Mesangioproliferative	Mesangioproliferative	Mesangioproliferative	FSGS	Mesangioproliferative
id: 153	CV fold 1	CV fold 2	CV fold 3	CV fold 4	CV fold 5

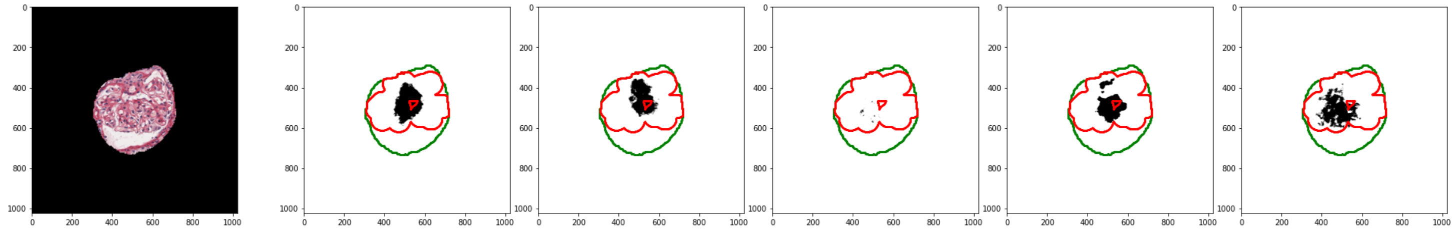

predicted area, %:	13.992	12.022	0.227	11.428	15.179
annotated area, %:	67.583	67.583	67.583	67.583	67.583
true label:	Mesangioproliferative	Mesangioproliferative	Mesangioproliferative	Mesangioproliferative	Mesangioproliferative
predicted label:	Mesangioproliferative	Mesangioproliferative	Mesangioproliferative	Mesangioproliferative	Mesangioproliferative
id: 154	CV fold 1	CV fold 2	CV fold 3	CV fold 4	CV fold 5

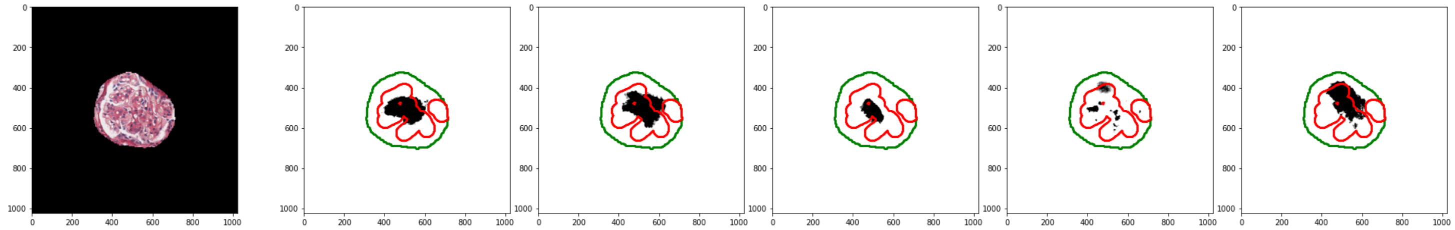

predicted area, %:	17.331	20.221	7.263	4.449	20.257
annotated area, %:	47.738	47.738	47.738	47.738	47.738
true label:	Mesangioproliferative	Mesangioproliferative	Mesangioproliferative	Mesangioproliferative	Mesangioproliferative
predicted label:	Mesangioproliferative	Mesangioproliferative	Mesangioproliferative	FSGS	Mesangioproliferative
id: 155	CV fold 1	CV fold 2	CV fold 3	CV fold 4	CV fold 5

Supplementary Figure S2 (continued). Comparison of pattern quantification results per glomerular basis.

Red contour: annotated area @ 0.5 threshold **Green contour:** total area **Black fill:** predicted area @ 0.5 threshold

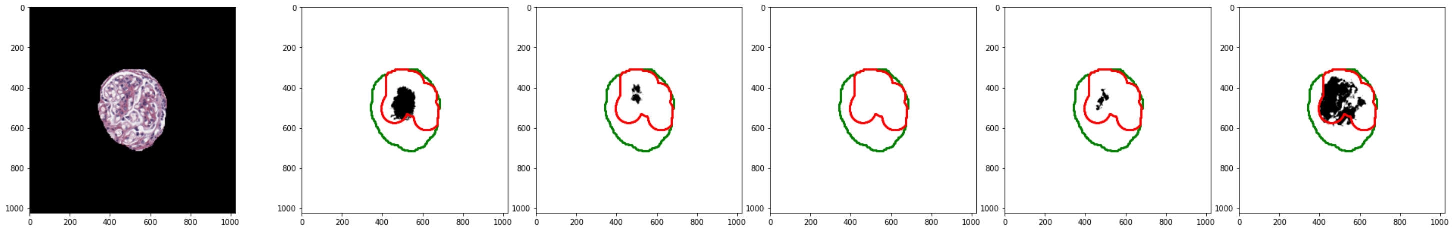

predicted area, %:	13.691	2.505	0.000	2.102	29.343
annotated area, %:	58.254	58.254	58.254	58.254	58.254
true label:	Mesangioproliferative	Mesangioproliferative	Mesangioproliferative	Mesangioproliferative	Mesangioproliferative
predicted label:	Mesangioproliferative	Mesangioproliferative	Mesangioproliferative	Mesangioproliferative	Mesangioproliferative
id: 156	CV fold 1	CV fold 2	CV fold 3	CV fold 4	CV fold 5

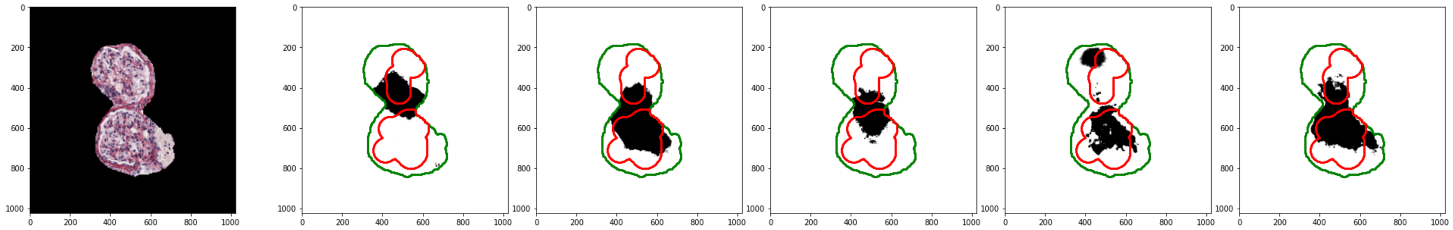

predicted area, %:	19.004	35.381	17.595	24.288	35.207
annotated area, %:	51.516	51.516	51.516	51.516	51.516
true label:	Mesangioproliferative	Mesangioproliferative	Mesangioproliferative	Mesangioproliferative	Mesangioproliferative
predicted label:	Crescentic	Crescentic	Endocapillary	Crescentic	Endocapillary
id: 157	CV fold 1	CV fold 2	CV fold 3	CV fold 4	CV fold 5

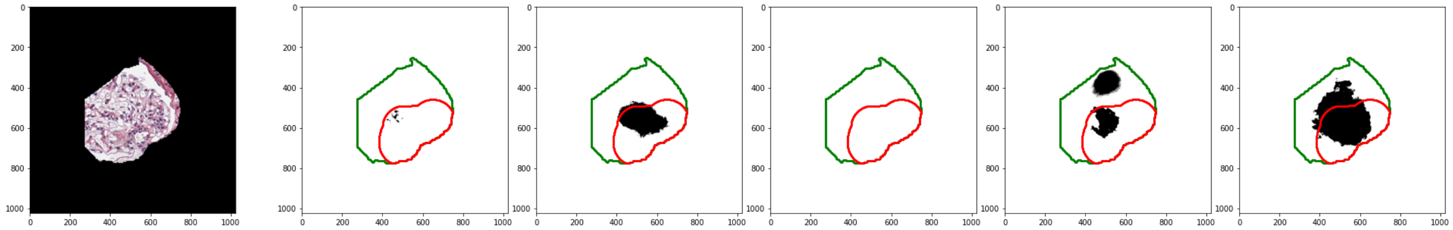

predicted area, %:	0.439	16.124	0.000	15.245	36.133
annotated area, %:	44.275	44.275	44.275	44.275	44.275
true label:	Mesangioproliferative	Mesangioproliferative	Mesangioproliferative	Mesangioproliferative	Mesangioproliferative
predicted label:	FSGS	FSGS	FSGS	FSGS	FSGS
id: 158	CV fold 1	CV fold 2	CV fold 3	CV fold 4	CV fold 5

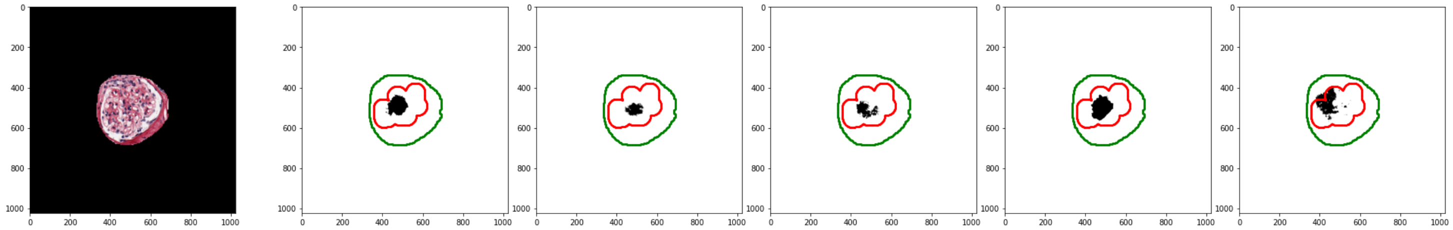

predicted area, %:	7.447	3.838	4.638	9.161	9.746
annotated area, %:	42.260	42.260	42.260	42.260	42.260
true label:	Mesangioproliferative	Mesangioproliferative	Mesangioproliferative	Mesangioproliferative	Mesangioproliferative
predicted label:	Mesangioproliferative	Normal	Mesangioproliferative	Membranous	Mesangioproliferative
id: 159	CV fold 1	CV fold 2	CV fold 3	CV fold 4	CV fold 5

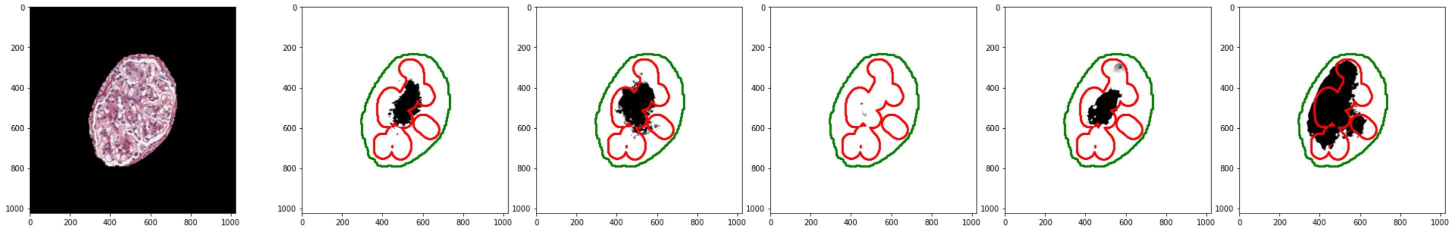

predicted area, %:	11.677	17.664	0.093	10.504	42.188
annotated area, %:	48.001	48.001	48.001	48.001	48.001
true label:	Mesangioproliferative	Mesangioproliferative	Mesangioproliferative	Mesangioproliferative	Mesangioproliferative
predicted label:	Mesangioproliferative	Mesangioproliferative	Mesangioproliferative	Mesangioproliferative	Mesangioproliferative
id: 160	CV fold 1	CV fold 2	CV fold 3	CV fold 4	CV fold 5

Supplementary Figure S2 (continued). Comparison of pattern quantification results per glomerular basis.

Red contour: annotated area @ 0.5 threshold **Green contour:** total area **Black fill:** predicted area @ 0.5 threshold

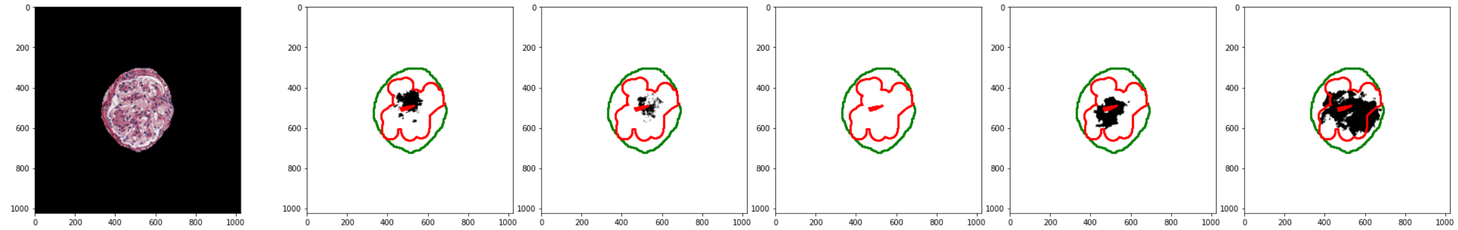

predicted area, %:	9.203	4.846	0.000	13.464	37.265
annotated area, %:	58.672	58.672	58.672	58.672	58.672
true label:	Mesangioproliferative	Mesangioproliferative	Mesangioproliferative	Mesangioproliferative	Mesangioproliferative
predicted label:	Mesangioproliferative	Mesangioproliferative	Mesangioproliferative	FSGS	Mesangioproliferative
id: 161	CV fold 1	CV fold 2	CV fold 3	CV fold 4	CV fold 5

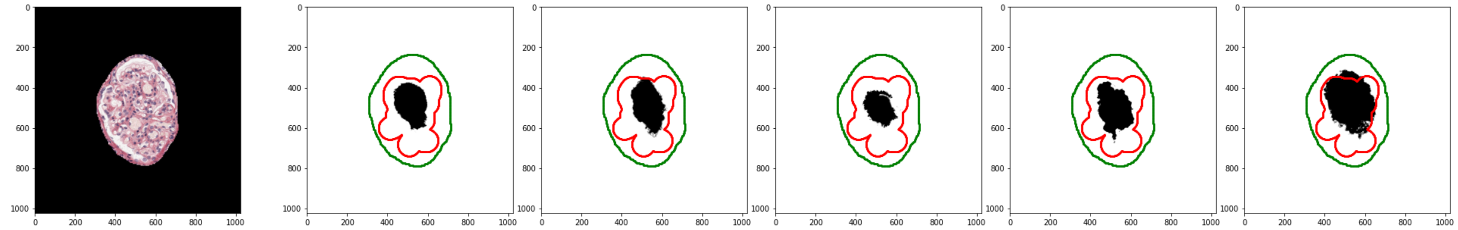

predicted area, %:	16.573	18.407	11.613	19.446	33.391
annotated area, %:	54.157	54.157	54.157	54.157	54.157
true label:	Mesangioproliferative	Mesangioproliferative	Mesangioproliferative	Mesangioproliferative	Mesangioproliferative
predicted label:	Mesangioproliferative	Membranoproliferative	Membranoproliferative	Membranoproliferative	Membranoproliferative
id: 162	CV fold 1	CV fold 2	CV fold 3	CV fold 4	CV fold 5

Supplementary Figure S2 (continued). Comparison of pattern quantification results per glomerular basis.

Red contour: annotated area @ 0.5 threshold **Green contour:** total area **Black fill:** predicted area @ 0.5 threshold
