# Supplementary material for: A Spatially Guided Machine-Learning Method to Classify and Quantify Glomerular Patterns of Injury in Histology Images
Source: J Imaging. 2023 Oct 11;9(10):220. doi: 10.3390/jimaging9100220 (PMC10607091; doi:10.3390/jimaging9100220)
Supplement: Supplementary file 1 [file jimaging-09-00220-s001.zip › Supplementary Table S1.pdf]

Supplementary table S1. Composition of One-vs-Rest training data set.

Positive class			Negative class		
	original	augmented		original	augmented
Crescentic	81	81			
			Endocapillary	10	10
			FSGS	10	10
			Hypertrophy	10	10
			Membranoproliferative	10	10
			Membranous	10	10
			Mesangioproliferative	10	10
			Normal	10	10
			Sclerosed	10	10
Total	162		Total	160	
Endocapillary	81	81	Crescentic	10	10
			FSGS	10	10
			Hypertrophy	10	10
			Membranoproliferative	10	10
			Membranous	10	10
			Mesangioproliferative	10	10
			Normal	10	10
			Sclerosed	10	10
Total	162		Total	160	
FSGS	81	81	Crescentic	10	10
			Endocapillary	10	10
			Hypertrophy	10	10
			Membranoproliferative	10	10
			Membranous	10	10
			Mesangioproliferative	10	10
			Normal	10	10
			Sclerosed	10	10
Total	162		Total	160	
Hypertrophy	81	81	Crescentic	10	10
			Endocapillary	10	10
			FSGS	10	10
			Membranoproliferative	10	10
			Membranous	10	10
			Mesangioproliferative	10	10
			Normal	10	10
			Sclerosed	10	10
Total	162		Total	160	
Membranoproliferative	81	81	Crescentic	10	10
			Endocapillary	10	10
			FSGS	10	10
			Hypertrophy	10	10
			Membranous	10	10
			Mesangioproliferative	10	10
			Normal	10	10
			Sclerosed	10	10
Total	162		Total	160	
Membranous	81	81	Crescentic	10	10
			Endocapillary	10	10
			FSGS	10	10
			Hypertrophy	10	10
			Membranoproliferative	10	10
			Mesangioproliferative	10	10
			Normal	10	10
			Sclerosed	10	10
Total	162		Total	160	

Supplementary table S1 (continued). Composition of One-vs-Rest training data set.

**Positive class**			**Negative class**		
	original	augmented		original	augmented
Mesangioproliferative	81	81	Crescentic	10	10
			Endocapillary	10	10
			FSGS	10	10
			Hypertrophy	10	10
			Membranoproliferative	10	10
			Membranous	10	10
			Normal	10	10
			Sclerosed	10	10
Total	162		Total	160	
Normal	81	81	Crescentic	10	10
			Endocapillary	10	10
			FSGS	10	10
			Hypertrophy	10	10
			Membranoproliferative	10	10
			Membranous	10	10
			Mesangioproliferative	10	10
			Sclerosed	10	10
Total	162		Total	160	
Sclerosed	81	81	Crescentic	10	10
			Endocapillary	10	10
			FSGS	10	10
			Hypertrophy	10	10
			Membranoproliferative	10	10
			Membranous	10	10
			Mesangioproliferative	10	10
			Normal	10	10
Total	162		Total	160	
