# Supplementary material for: A Spatially Guided Machine-Learning Method to Classify and Quantify Glomerular Patterns of Injury in Histology Images
Source: J Imaging. 2023 Oct 11;9(10):220. doi: 10.3390/jimaging9100220 (PMC10607091; doi:10.3390/jimaging9100220)
Supplement: Supplementary file 1 [file jimaging-09-00220-s001.zip › Supplementary Tables S2-S3.pdf]

Supplementary table S2. Confusion matrices from cross validation of all classifiers (Single-multiclass).

Single-multiclass		Predicted labels									
	True labels	Crescentic	Endocapillary	FSGS	Hypertrophy	Membranoproliferative	Membranous	Mesangioproliferative	Normal	Sclerosed	Sum
Fold1	Crescentic	25	0	1	1	0	0	2	0	0	29
	Endocapillary	6	24	2	0	0	0	4	1	0	37
	FSGS	6	1	27	5	2	3	6	4	0	54
	Hypertrophy	0	0	0	29	0	1	1	2	0	33
	Membranoproliferative	3	5	0	0	34	1	2	1	0	46
	Membranous	0	1	3	1	0	29	1	0	0	35
	Mesangioproliferative	0	2	6	2	0	2	30	0	0	42
	Normal	0	0	6	10	0	8	9	63	0	96
	Sclerosed	0	0	1	0	0	0	0	0	44	45
Fold2	Crescentic	23	3	0	0	1	0	2	0	0	29
	Endocapillary	2	31	0	0	2	0	1	1	0	37
	FSGS	4	5	21	5	3	3	9	4	0	54
	Hypertrophy	0	0	0	26	0	2	1	4	0	33
	Membranoproliferative	0	9	0	0	36	0	1	0	0	46
	Membranous	1	1	0	1	1	28	3	0	0	35
	Mesangioproliferative	0	3	2	0	8	1	28	0	0	42
	Normal	1	3	2	6	1	5	20	58	0	96
	Sclerosed	0	0	1	0	0	0	0	0	44	45
Fold3	Crescentic	26	1	1	0	0	0	1	0	0	29
	Endocapillary	2	32	2	0	0	0	0	1	0	37
	FSGS	5	3	32	5	2	1	3	3	0	54
	Hypertrophy	0	0	1	30	0	0	1	1	0	33
	Membranoproliferative	1	13	1	0	31	0	0	0	0	46
	Membranous	0	1	5	1	0	25	2	1	0	35
	Mesangioproliferative	0	5	17	0	3	1	16	0	0	42
	Normal	1	2	15	10	0	3	5	60	0	96
	Sclerosed	1	0	0	0	0	0	0	0	44	45
Fold4	Crescentic	25	0	1	1	1	0	1	0	0	29
	Endocapillary	4	26	1	0	3	1	1	1	0	37
	FSGS	7	3	25	5	4	4	1	5	0	54
	Hypertrophy	0	0	0	31	0	0	0	2	0	33
	Membranoproliferative	1	8	0	0	37	0	0	0	0	46
	Membranous	0	0	1	1	1	30	1	1	0	35
	Mesangioproliferative	1	3	12	2	2	2	20	0	0	42
	Normal	0	0	9	12	0	8	3	64	0	96
	Sclerosed	0	0	1	0	0	0	0	0	44	45
Fold5	Crescentic	23	0	1	1	1	0	3	0	0	29
	Endocapillary	3	22	2	0	5	1	2	2	0	37
	FSGS	3	1	24	6	3	4	7	4	2	54
	Hypertrophy	0	0	1	29	0	1	0	2	0	33
	Membranoproliferative	2	4	0	0	38	0	1	1	0	46
	Membranous	0	0	0	1	1	31	2	0	0	35
	Mesangioproliferative	0	1	3	1	4	3	29	1	0	42
	Normal	0	0	7	10	1	10	6	62	0	96
	Sclerosed	0	0	1	0	0	0	0	0	44	45

Supplementary table S2 (continued). Confusion matrices from cross validation of all classifiers (Multiple-binary).

Multiple-binary		Predicted labels									
	True labels	Crescentic	Endocapillary	FSGS	Hypertrophy	Membranoproliferative	Membranous	Mesangioproliferative	Normal	Sclerosed	Sum
Fold1	Crescentic	23	0	0	2	1	0	2	0	1	29
	Endocapillary	7	14	1	3	5	0	3	3	1	37
	FSGS	4	2	22	8	3	2	4	6	3	54
	Hypertrophy	1	0	0	29	0	2	1	0	0	33
	Membranoproliferative	3	4	0	0	39	0	0	0	0	46
	Membranous	0	0	0	2	2	27	4	0	0	35
	Mesangioproliferative	1	1	3	0	11	2	21	3	0	42
	Normal	0	0	7	13	1	6	8	59	2	96
	Sclerosed	0	0	1	0	0	0	0	0	44	45
Fold2	Crescentic	24	0	1	1	1	0	1	0	1	29
	Endocapillary	8	17	0	1	5	0	3	2	1	37
	FSGS	6	1	25	7	3	2	4	6	0	54
	Hypertrophy	1	0	1	25	2	1	0	3	0	33
	Membranoproliferative	2	4	1	0	37	1	1	0	0	46
	Membranous	0	0	0	2	1	29	2	1	0	35
	Mesangioproliferative	3	0	8	0	9	2	20	0	0	42
	Normal	1	0	12	10	3	3	3	63	1	96
	Sclerosed	0	0	1	0	0	0	0	0	44	45
Fold3	Crescentic	20	2	2	1	1	0	1	0	2	29
	Endocapillary	5	24	1	2	1	0	2	1	1	37
	FSGS	1	3	23	7	3	5	3	6	3	54
	Hypertrophy	1	0	1	27	0	3	0	1	0	33
	Membranoproliferative	1	8	1	0	35	1	0	0	0	46
	Membranous	0	0	2	1	1	29	2	0	0	35
	Mesangioproliferative	1	1	9	0	5	1	22	3	0	42
	Normal	1	0	12	11	0	8	2	61	1	96
	Sclerosed	0	0	1	0	0	0	0	0	44	45
Fold4	Crescentic	22	1	3	1	0	0	1	0	1	29
	Endocapillary	3	24	0	1	1	0	5	2	1	37
	FSGS	4	2	23	10	2	2	5	5	1	54
	Hypertrophy	1	0	0	27	0	3	1	1	0	33
	Membranoproliferative	2	8	1	0	34	0	1	0	0	46
	Membranous	0	0	1	2	0	31	1	0	0	35
	Mesangioproliferative	0	1	8	1	4	3	24	1	0	42
	Normal	1	0	12	10	2	8	9	54	0	96
	Sclerosed	0	0	1	0	0	0	0	0	44	45
Fold5	Crescentic	19	4	3	1	0	0	0	0	2	29
	Endocapillary	3	27	1	1	1	0	1	3	0	37
	FSGS	4	3	25	7	3	3	2	4	3	54
	Hypertrophy	0	0	1	29	1	1	0	1	0	33
	Membranoproliferative	0	13	1	0	29	2	1	0	0	46
	Membranous	0	0	1	2	0	31	1	0	0	35
	Mesangioproliferative	1	3	14	0	4	4	15	1	0	42
	Normal	1	1	8	8	1	9	5	63	0	96
	Sclerosed	0	0	1	0	0	0	0	0	44	45

Supplementary table S2 (continued). Confusion matrices from cross validation of all classifiers (Spatially-guided).

Spatially-guided		Predicted labels									Sum
	True labels	Crescentic	Endocapillary	FSGS	Hypertrophy	Membranoproliferative	Membranous	Mesangioproliferative	Normal	Sclerosed	
Fold1	Crescentic	24	2	1	1	0	0	1	0	0	29
	Endocapillary	3	25	2	0	5	1	0	1	0	37
	FSGS	4	3	26	3	3	5	6	4	0	54
	Hypertrophy	0	0	0	31	0	1	0	1	0	33
	Membranoproliferative	3	4	0	0	36	0	3	0	0	46
	Membranous	0	0	0	1	1	32	0	1	0	35
	Mesangioproliferative	0	2	6	0	5	1	28	0	0	42
	Normal	0	0	4	8	1	12	5	66	0	96
	Sclerosed	0	0	0	0	0	0	0	0	45	45
Fold2	Crescentic	26	1	0	1	0	0	1	0	0	29
	Endocapillary	5	24	2	0	3	0	2	1	0	37
	FSGS	3	2	29	3	3	2	7	4	1	54
	Hypertrophy	0	0	0	31	0	0	1	1	0	33
	Membranoproliferative	4	4	0	0	34	0	4	0	0	46
	Membranous	1	1	2	1	1	25	3	1	0	35
	Mesangioproliferative	1	0	7	0	3	1	30	0	0	42
	Normal	0	1	5	9	1	0	15	65	0	96
	Sclerosed	0	0	0	0	0	0	0	0	45	45
Fold3	Crescentic	24	0	2	1	1	0	0	0	1	29
	Endocapillary	7	21	2	0	5	0	1	1	0	37
	FSGS	3	1	33	4	1	6	1	4	1	54
	Hypertrophy	0	0	2	28	0	1	0	2	0	33
	Membranoproliferative	5	2	1	0	36	0	2	0	0	46
	Membranous	0	0	1	1	1	31	0	1	0	35
	Mesangioproliferative	2	0	15	0	5	3	16	1	0	42
	Normal	0	0	4	7	1	10	2	72	0	96
	Sclerosed	0	0	0	0	0	0	0	0	45	45
Fold4	Crescentic	24	3	0	1	0	0	1	0	0	29
	Endocapillary	1	33	2	0	1	0	0	0	0	37
	FSGS	4	5	23	6	3	4	7	2	0	54
	Hypertrophy	0	0	2	31	0	0	0	0	0	33
	Membranoproliferative	1	13	0	0	29	0	3	0	0	46
	Membranous	1	1	0	1	1	28	3	0	0	35
	Mesangioproliferative	0	3	5	0	5	1	28	0	0	42
	Normal	1	4	10	15	1	6	17	42	0	96
	Sclerosed	0	0	0	0	0	0	0	0	45	45
Fold5	Crescentic	20	2	1	2	0	0	3	0	1	29
	Endocapillary	5	22	1	0	4	0	4	1	0	37
	FSGS	2	1	25	6	4	3	11	2	0	54
	Hypertrophy	0	0	0	32	0	0	0	1	0	33
	Membranoproliferative	2	6	0	0	35	0	3	0	0	46
	Membranous	0	0	0	1	1	31	2	0	0	35
	Mesangioproliferative	1	0	2	1	3	1	33	1	0	42
	Normal	0	0	1	12	0	7	12	64	0	96
	Sclerosed	0	0	0	0	0	0	0	0	45	45

Supplementary table S3. AUC scores from cross validation of all classifiers.

	Patterns	Single-multiclass	Multiple-binary	Spatially-guided
Fold1	Crescentic	0.9758	0.9444	0.9747
	Endocapillary	0.9657	0.9253	0.9674
	FSGS	0.8318	0.7606	0.8712
	Hypertrophy	0.9729	0.9671	0.9803
	Membranoproliferative	0.9631	0.9464	0.9537
	Membranous	0.975	0.9543	0.9672
	Mesangioproliferative	0.9197	0.8889	0.9092
	Normal	0.9542	0.9407	0.9688
	Sclerosed	0.9947	0.9909	0.9949
Fold2	Crescentic	0.9709	0.9441	0.9688
	Endocapillary	0.9581	0.9177	0.969
	FSGS	0.8615	0.7685	0.8722
	Hypertrophy	0.9637	0.9708	0.9852
	Membranoproliferative	0.966	0.9506	0.9618
	Membranous	0.9652	0.9525	0.9698
	Mesangioproliferative	0.9324	0.9018	0.9242
	Normal	0.938	0.9302	0.9639
	Sclerosed	0.9947	0.9911	0.9949
Fold3	Crescentic	0.972	0.9396	0.9737
	Endocapillary	0.9584	0.9251	0.9742
	FSGS	0.8382	0.7752	0.8517
	Hypertrophy	0.9662	0.9662	0.9818
	Membranoproliferative	0.9653	0.9449	0.9596
	Membranous	0.9716	0.9508	0.9719
	Mesangioproliferative	0.9122	0.8862	0.9226
	Normal	0.938	0.9348	0.9615
	Sclerosed	0.9947	0.9916	0.9949
Fold4	Crescentic	0.9629	0.9333	0.9695
	Endocapillary	0.9671	0.9139	0.9731
	FSGS	0.8466	0.7647	0.8862
	Hypertrophy	0.9711	0.9723	0.9812
	Membranoproliferative	0.9719	0.9508	0.9537
	Membranous	0.9701	0.9537	0.9756
	Mesangioproliferative	0.9263	0.8679	0.9162
	Normal	0.9392	0.9263	0.9639
	Sclerosed	0.9947	0.9907	0.9949
Fold5	Crescentic	0.9734	0.9131	0.9685
	Endocapillary	0.9728	0.9136	0.9712
	FSGS	0.8238	0.7639	0.8346
	Hypertrophy	0.976	0.9717	0.9781
	Membranoproliferative	0.9585	0.9466	0.9495
	Membranous	0.9693	0.9519	0.9747
	Mesangioproliferative	0.9081	0.8862	0.9007
	Normal	0.947	0.9411	0.9617
	Sclerosed	0.9947	0.9907	0.9949

Supplementary table S4. IoU scores (segmental injury patterns only) from cross validation of all classifiers.

	Patterns	Single-multiclass	Multiple-binary	Spatially-guided
Fold1	Crescentic	0.053	0.056	0.532
	Endocapillary	0.046	0.047	0.452
	FSGS	0.043	0.024	0.327
	Mesangioproliferative	0.040	0.035	0.233
Fold2	Crescentic	0.068	0.058	0.436
	Endocapillary	0.042	0.058	0.449
	FSGS	0.044	0.053	0.264
	Mesangioproliferative	0.036	0.068	0.265
Fold3	Crescentic	0.070	0.070	0.144
	Endocapillary	0.056	0.052	0.157
	FSGS	0.046	0.037	0.092
	Mesangioproliferative	0.045	0.032	0.075
Fold4	Crescentic	0.071	0.054	0.329
	Endocapillary	0.050	0.060	0.338
	FSGS	0.037	0.026	0.232
	Mesangioproliferative	0.041	0.044	0.208
Fold5	Crescentic	0.045	0.061	0.579
	Endocapillary	0.058	0.043	0.501
	FSGS	0.043	0.031	0.401
	Mesangioproliferative	0.041	0.063	0.393
